# Temporary carbon storage can mitigate slow-responding climate changes

**DOI:** 10.1038/s41467-026-78620-8

**Published:** 2026-10-16

**Authors:** Mitchell Dickau, H. Damon Matthews

**Affiliations:** Department of Geography, Planning and Environment, Concordia University, Montréal, Quebec Canada

**Keywords:** Climate and Earth system modelling, Climate-change impacts, Climate-change mitigation

## Abstract

Carbon removal and storage is central to many net-zero commitments, yet its durability, particularly for nature-based methods, is uncertain due to disturbance risks. Previous work shows that carbon storage-years (the time-integrated quantity of carbon storage) are proportional to time-integrated avoided warming, which in turn causes avoided changes in slow-responding climate variables. Here, we show that temporary carbon storage results in avoided changes in inertial climate variables. Avoided changes in 2100 are proportional to carbon storage-years across a range of storage amounts and emissions scenarios for permafrost carbon, ocean carbon, Atlantic meridional overturning circulation, ocean temperature, and thermosteric sea-level rise. By 2300, proportionality persists in some variables, notably sea-level rise, ocean temperature, and ocean carbon. Temporary storage therefore has climate benefits that extend beyond the duration of the storage, with some persistence of benefits even on multi-century timescales. We find that these climate benefits are greatest when temporary storage is paired with ambitious mitigation.

## Abstract (web summary)

Temporary carbon removal and storage can lead to avoided changes in slow responding climate variables that extend beyond the duration of storage itself. These climate benefits are greater when storage is paired with ambitious climate mitigation.

## Introduction

Carbon removal and storage has emerged as a prominent element in climate mitigation portfolios for both nations^1^ and corporations^2^. Much of this proposed carbon removal is nature-based, making it vulnerable to disturbances such as fire, disease, or land-use change^3,4^. Because the durability of carbon storage is uncertain due to these threats, and because the durability of carbon storage dictates the persistence of the temperature effects of carbon removal and storage^5,6^, the extent to which temporary carbon storage can reduce climate risk is difficult to quantify. In contrast, the climate impacts of fossil CO_2_ emissions remain for thousands of years^7,8^. This asymmetry creates a fundamental challenge: carbon removal achieved through methods that may only provide temporary storage is unlikely to provide a lasting offset to the warming and subsequent climate changes driven by fossil CO_2_ emissions.

Nevertheless, even temporary carbon storage, either from avoided emissions or from non-permanent carbon removal, yields measurable and meaningful climate benefits by reducing peak global temperature in ambitious climate mitigation scenarios and by delaying global temperature changes in less ambitious scenarios^6^. These outcomes can be significant, as the magnitude and timing of peak warming influence both the severity of climate impacts and the time available for adaptation and mitigation responses. The potential that some carbon storage may only be temporary should therefore not lead to the conclusion that methods of carbon storage with a high possibility of being temporary, such as nature-based solutions, are ineffectual. Rather, temporary carbon storage should be recognized as an important contribution to climate risk reduction, while acknowledging the fundamental asymmetry between temporary carbon storage and the long-lived effects of fossil CO_2_ emissions^5,9,10^.

Efforts to formalize the effect of temporary carbon storage on temperature date back more than two decades, when storage-year (a.k.a. tonne-year) and equivalence-time metrics were first proposed to compare permanent and non-permanent carbon storage^11–13^. These concepts were subsequently adopted within industrial ecology and life-cycle assessment methodologies to develop dynamic carbon accounting frameworks^14–18^. More recent work has demonstrated a consistent relationship between the magnitude and duration of temporary carbon storage and the effect of this storage on temperature, and has reinforced that time-integrated measures of storage capture this relationship more effectively than simple stock measures^19–24^. Existing evaluations have quantified these relationships using analytical accounting formulations, localized ecosystem carbon-cycle models, and an intermediate-complexity climate model^19–24^. Matthews et al.^23^ showed that temporary storage-years of carbon, which represent the time-integrated amount of annual additional carbon storage, are approximately proportional to degree-years of avoided warming, which represent the time-integrated amount of avoided warming. This proportionality provides a framework for quantifying the climate value of non-permanent sequestration. Extending this work, Dickau et al.^25^ establish that degree-years of avoided warming are proportional to avoided changes in a suite of inertial climate system variables in scenarios with temperature overshoot. Together, these findings imply that carbon storage-years provide a metric for linking temporary sequestration to avoided changes in inertial climate variables. This framework provides a rigorous basis for evaluating the role of nature-based carbon storage in climate mitigation and for comparing the climate benefits of temporary and permanent carbon storage.

**Fig. 1:**
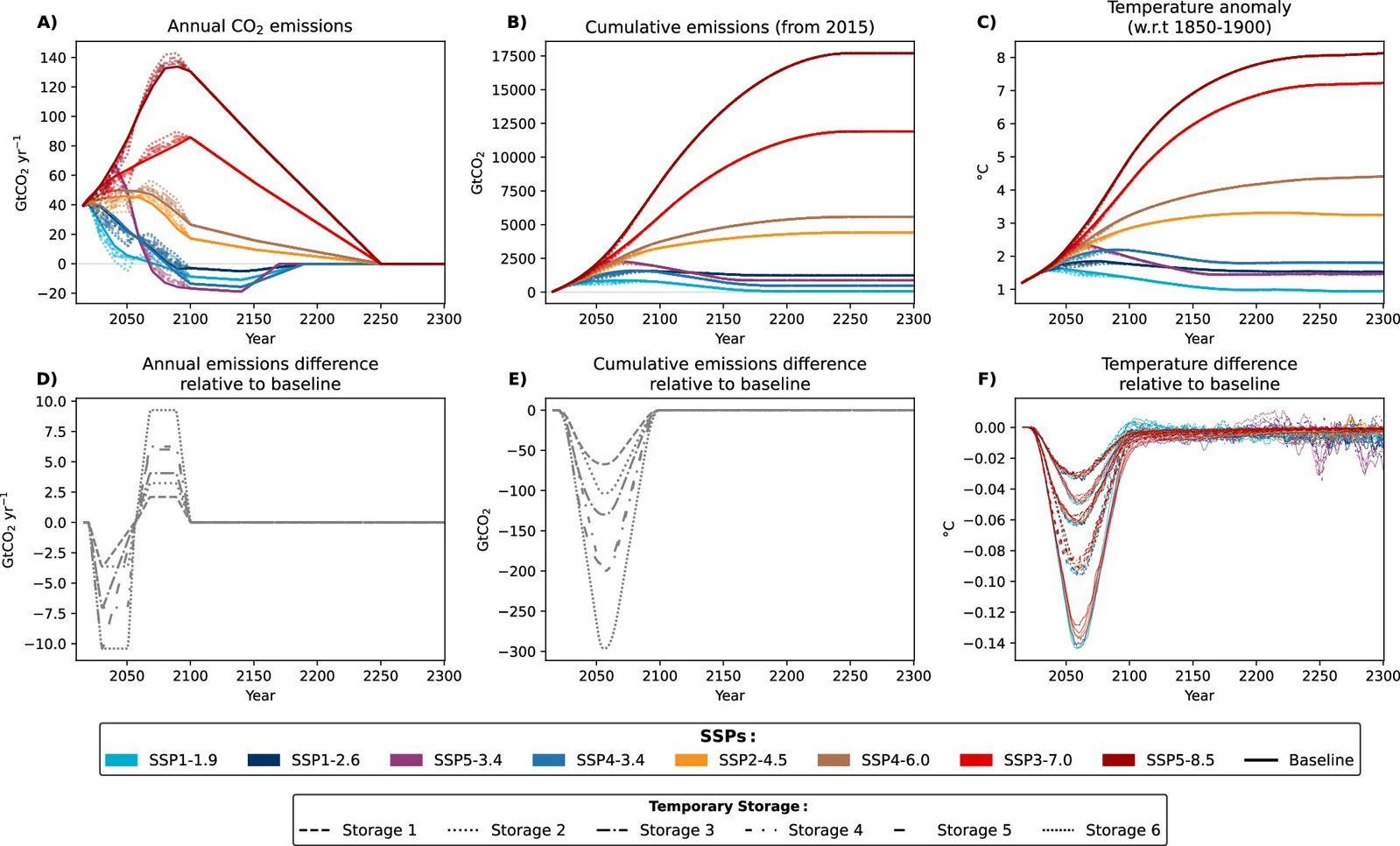
Anthropogenic CO₂ emissions and temperature responses in reference and storage SSPs. The first row shows the evolution of **A** annual CO_2_ emissions from fossil fuels and land-use change, **B** cumulative CO_2_ emissions, and **C** global mean surface air temperature anomaly relative to an 1850–1900 preindustrial baseline from 2015 to 2300. The second row shows differences between storage SSPs and their corresponding reference SSPs for **D** annual CO_2_ emissions, **E** cumulative CO_2_ emissions, and **F** temperature. Colors indicate SSP ensembles. In subplots A–C, solid lines represent reference simulations and dashed/dotted lines represent the six time-limited carbon storage pathways applied to each reference SSP. In **D** and **E**, gray lines are used because storage pathways are identical across all reference SSPs. Subplots **D**–**F** represent only the difference between reference and temporary storage SSPs, there are no solid lines representing reference SSPs.

Here, we use an intermediate-complexity global climate model – the University of Victoria Earth System Climate Model (UVic-ESCM)^26,27^ – to investigate the proportionality between temporary carbon storage, expressed in storage-years, and changes in highly inertial components of the climate system. We simulate a suite of idealized temporary storage pathways (with decreased emissions followed by re-emission of stored carbon) applied to reference Shared Socioeconomic Pathway (SSP) scenarios to approximate the biogeochemical effect of time-limited carbon storage interventions. Our simulations include eight reference simulations following the SSP-RCP scenarios established in CMIP6 and six temporary storage pathways applied to each SSP-RCP reference simulation to generate an ensemble of 48 temporary storage simulations (hereinafter storage SSPs) and eight reference simulations (hereinafter reference SSPs) (Fig. 1). See Methods for more details. We then quantify the effect of this temporary carbon storage up to 2300 on changes in key slow-responding climate system variables – permafrost carbon, ocean temperature, total ocean carbon, thermosteric sea level rise, and maximum Atlantic meridional overturning circulation (AMOC). Our results show a clear proportionality between storage-years of temporary carbon storage and degree-years of avoided warming, with measurable benefits for both global temperature and the avoidance of changes in slow-responding climate variables. For temperature, the effect is most apparent in ambitious mitigation scenarios, where temporary removal reduces peak warming. For slow-responding globally averaged ocean variables, avoided changes generally scale proportionally with storage-years in both 2100 and 2300 across SSPs. For permafrost carbon, mitigated changes are generally proportional to storage-years within individual SSP ensembles in both 2100 and 2300, demonstrating persistent scenario-dependent proportionality. Conversely, while the AMOC exhibits a similar scenario-dependent relationship in 2100, this proportionality breaks down completely by 2300 due to decadal-scale ocean dynamics that affect the AMOC behavior after the year 2100. We also find that the relative climate benefits of temporary storage are greater when paired with ambitious mitigation. When compared to pathways where equal amounts of carbon are permanently removed, temporary storage SSPs exhibit significantly less avoided change, which shows that the false assumption of permanence could lead to the overestimation of the benefit of temporary carbon storage.

## Results

### Temperature effect of temporary carbon storage

**Fig. 2:**
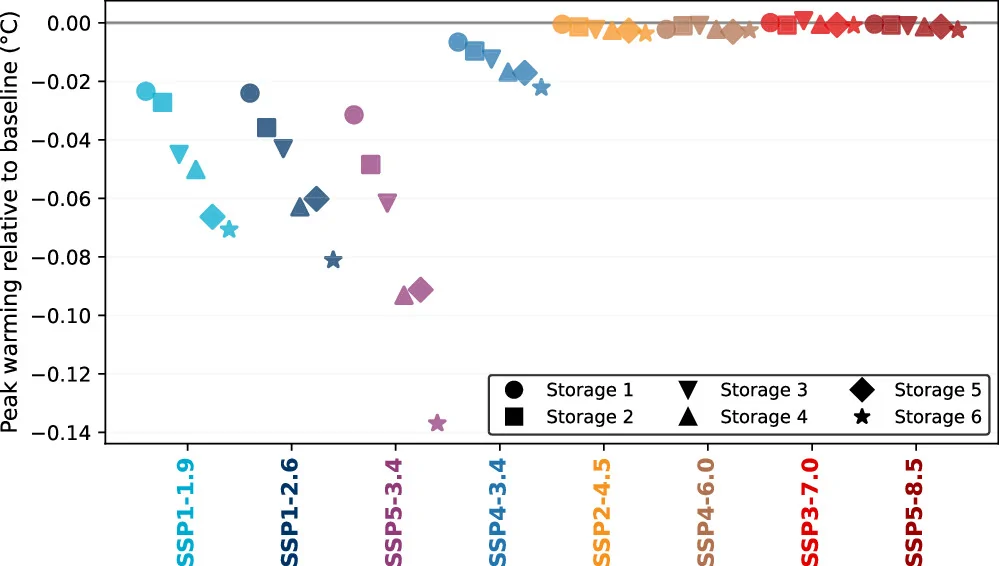
Effect of temporary carbon storage on peak warming across temporary storage scenarios. The difference in peak global mean temperature between temporary carbon storage SSPs and their corresponding reference SSPs. Negative values indicate a lower peak in storage SSPs relative to their reference SSP as a result of temporary storage. Colors indicate the reference SSP ensemble, and marker shapes differentiate the six time-limited carbon storage pathways that are applied to each reference SSP.

Our simulations confirm that temporary carbon storage occurring in the first half of the 21^st^ century decreases peak temperature in scenarios with a peak-and-decline temperature trajectory (i.e., SSP1-1.9, SSP1-2.6, SSP4-3.4, SSP5-3.4; all of which are scenarios that reach net zero followed by net negative CO_2_ emissions), but only delays warming in high-emissions scenarios with continued temperature increase past 2100 (i.e., SSP2-4.5, SSP4-6.0, SSP3-7.0, SSP5-8.5) (Fig. 2). In 2100, nearly all the temperature difference between storage and reference SSPs is reversed, leaving only a small residual cooling effect in some storage SSPs (Fig. 1F). This residual temperature difference between storage SSPs and reference SSPs in 2100 is larger in scenarios with higher end-of-century warming and scales with the magnitude of temporary storage (e.g., Fig. 1F; SSP5-8.5 Storage 6 has the largest residual temperature difference in 2100). After 2100, the temperature differences and increased variability caused by temporary storage are greater in SSPs with peak-and-decline temperature trajectories than in high-emissions SSPs that do not reach net-negative CO_2_ emissions throughout the simulations (Fig. 1F).

Regional temperature difference following 2100 between storage and reference SSPs is most pronounced at the poles, where slight cooling is evident, while the low and middle latitudes experience little change or slight warming (Supplementary Fig. 1). This regional pattern of temperature difference is consistent with several studies, which show that the poles are generally warmer following temperature overshoot^25,28,29^. Here, however, reference SSPs act as overshoot scenarios relative to the storage SSPs, which is why the poles in storage SSPs are cooler following temporary carbon storage. As with global mean temperature, these regional patterns become more pronounced with larger magnitudes of temporary carbon storage and higher end-of-century warming (e.g., Supplementary Fig. 1; SSP5-8.5 Storage pathway 6 has the largest high latitude cooling). By 2300, most of the regional differences in temperature between storage and reference SSPs are erased for high-emissions SSPs that do not reach net-negative CO_2_ (Supplementary Fig. 2). However, in peak-and-decline SSPs, temporary storage continues to cause regional temperature differences relative to reference SSPs (Supplementary Fig. 2). These regional temperature differences of both cooling and warming in 2300 are concentrated in the northern high latitudes, particularly the North Atlantic, and are likely driven by increased AMOC variability seen in peak-and-decline SSPs (discussed below).

**Fig. 3:**
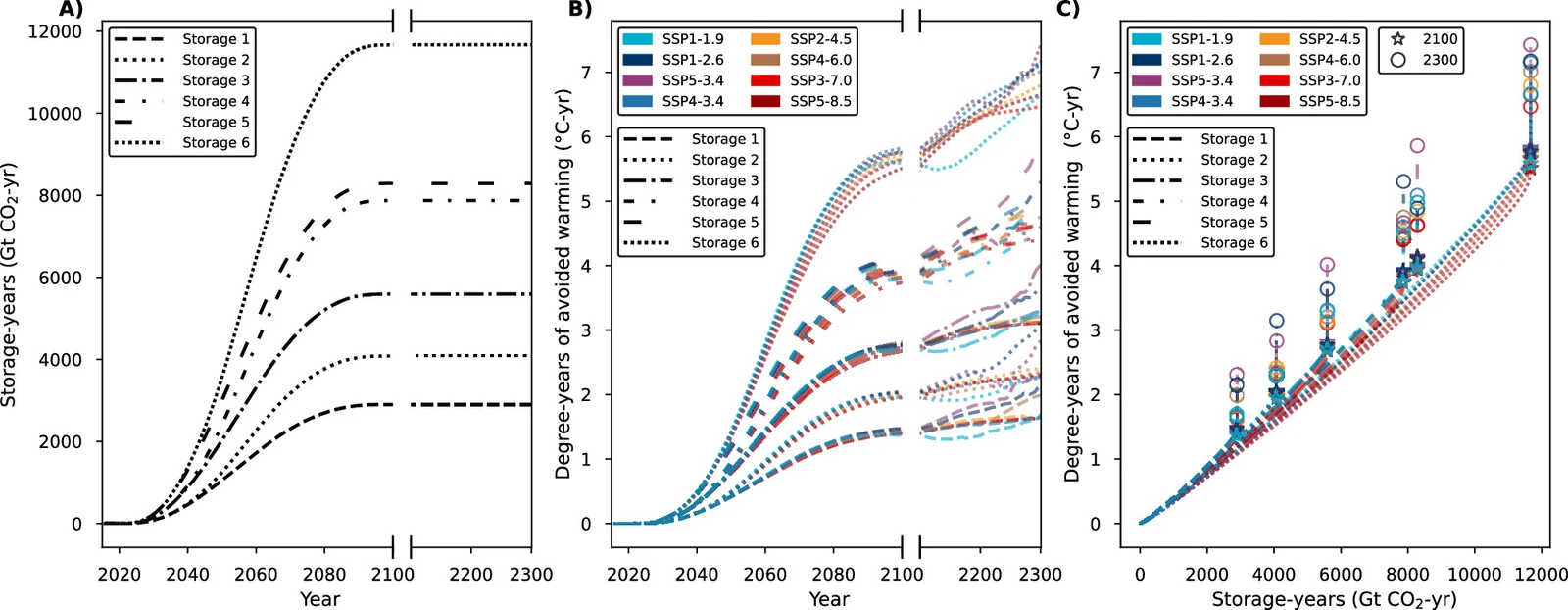
Storage-years of temporary carbon storage and degree-years of avoided warming in storage SSPs. This figure shows **A** the evolution of storage-years of the six storage pathways, **B** the evolution of degree-years of avoided warming across the storage SSPs, and **C** the quasi-linear relationship between degree-years of avoided warming and storage-years of temporary carbon storage across storage SSPs. In all three panels, line types represent the six time-limited storage pathways applied to reference SSPs. In panels **B** and **C**, colors represent reference SSPs. In panel C, stars represent values at 2100, and open circles represent values at 2300.

We calculate the storage-years of temporary carbon storage and the degree-years of avoided warming of the storage SSPs relative to their reference SSPs. Consistent with previous work^23^, we find a linear relationship between storage-years of temporary carbon removal and degree-years of avoided warming in our simulations in 2100 (R^2^ 0.998) (Fig. 3C, Supplementary Fig. 3A), across a wider range of SSPs and temporary storage pathways as compared to previous work. This proportionality indicates that the duration and magnitude of carbon storage directly determine the duration and magnitude of avoided warming. This proportionality is grounded in the linear relationship between global mean temperature increase and cumulative CO₂ emissions, as described by the transient climate response to cumulative carbon emissions (TCRE)^30–34^. The linearity in the TCRE arises because each tonne of CO₂ produces a similar amount of warming regardless of when it is emitted or the background climate state^35^, and because warming from CO_2_ persists for thousands of years^7,8,36^. Consequently, a CO_2_ removal (or additional avoided emission) and an additional emission exert nearly equal but opposite effects on global temperature regardless of the timing of emissions or the background concentration of CO_2_^37,38^. When time-integrated, this TCRE relationship extends to a proportionality between carbon storage-years and degree-years of avoided warming in 2100 (Fig. 3C).

Because storage SSPs remain slightly cooler than their corresponding reference scenarios after 2100 (Fig. 1F), degree-years of avoided warming continue to accumulate between 2100 and 2300 (Fig. 3B). This accumulation of degree-years is driven in part by the zero emissions commitment (ZEC). The simulated ZEC of our model is close to zero during the first century following the cessation of positive emissions, but increases to cause slow warming over multi-century timescales^39^. For pathways with reduced or negative emissions relative to a reference scenario, we would expect that the model’s positive multi-century ZEC value would cause sustained cooling and the accumulation of degree-years observed here in the storage SSPs. This multi-century accumulation of degree-years is likely caused by reduced permafrost carbon release in the storage SSPs, which is also the mechanism behind the model’s positive multi-century ZEC^39^. Evidence for this permafrost mechanism can be seen in the accumulation of degree-years of avoided warming being generally more pronounced in peak-and-decline SSPs (Fig. 3B, C), which also have more long-term avoided permafrost carbon loss compared to the higher-emission SSP scenarios (discussed below). As a result of sustained lower temperature in storage SSPs, especially those with peak-and-decline temperature trajectories, each unit of storage-years yields progressively more degree-years of avoided warming as 2300 is approached, even though storage-years remain constant after 2100 (Fig. 3). Consequently, while a clear linear pattern between storage-years and degree-years persists through 2300 across ensembles (R^2^ 0.968), it is accompanied by stronger scenario dependence and has a different slope relative to that in 2100 (Fig. 3C, Supplementary Fig. 3).

### Effect of temporary carbon storage on slow-responding climate variables

**Fig. 4:**
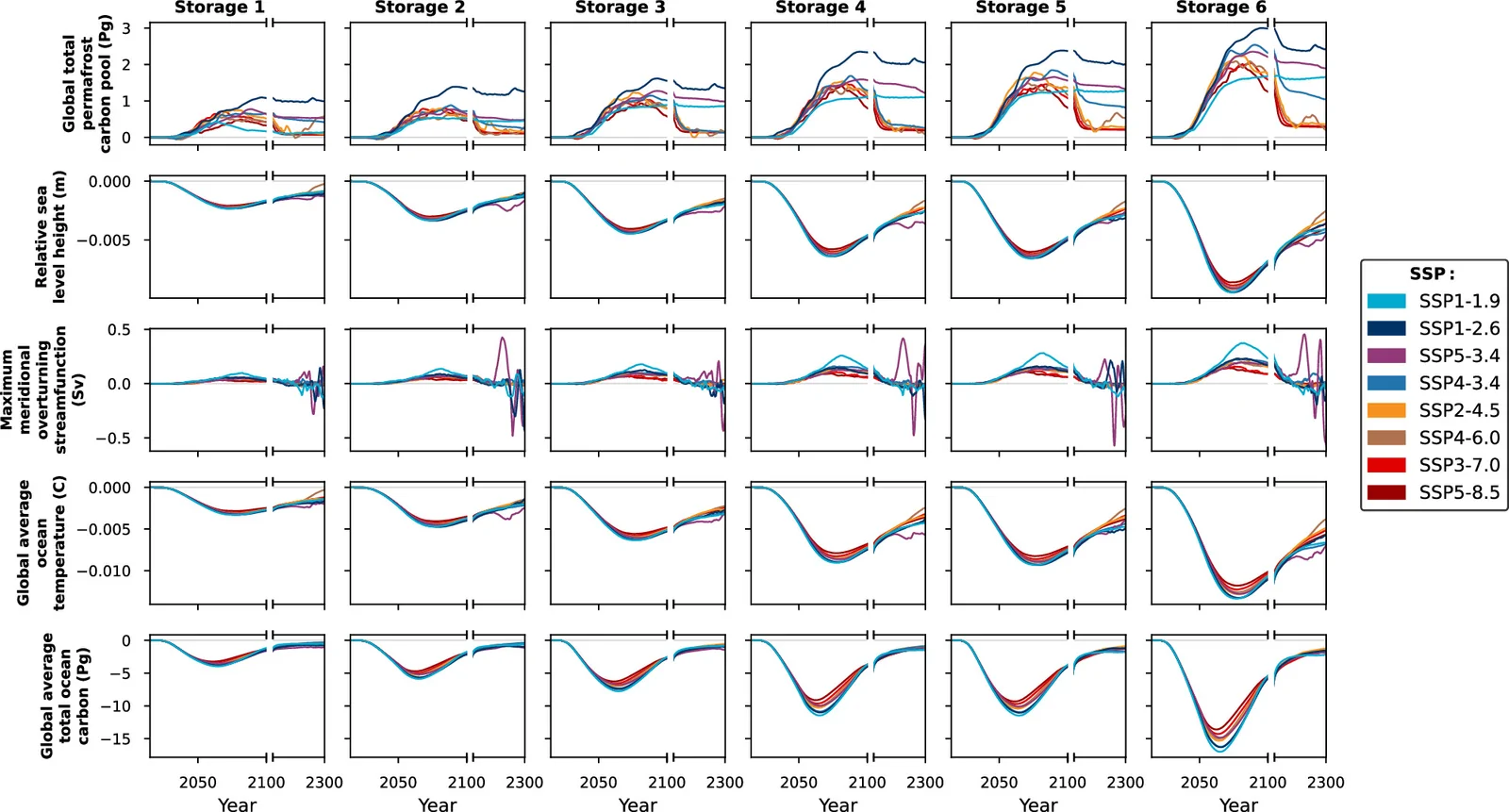
Differences in slow-responding climate variables between storage and reference SSPs to 2300. Each subplot shows the difference between storage and reference SSPs for a single slow-responding variable within a single storage pathway. Rows correspond to climate variables (listed on the left), and columns correspond to storage pathways (listed above). Storage pathways 1 to 6 represent time-limited carbon storage applied to reference SSPs. All variables show avoided change from temporary carbon storage up to 2100, with the magnitude of avoided change increasing under storage pathways with greater storage. From 2100 to 2300, variables generally show that the effect of temporary carbon storage diminishes except for maximum Atlantic meridional overturning, which sees a wide range of responses. Colored lines in each subplot indicate the underlying SSP for each of the storage simulations.

Unlike the effects of temporary carbon storage on temperature, slow-responding climate variables show continued but declining avoided changes in 2100 despite temporary carbon storage having already been re-emitted by 2100 (Fig. 4). Figure 4 shows the difference between slow-responding variables in the storage SSPs relative to their reference SSPs. Across all variables, the magnitude of avoided change relative to reference SSPs increases with the magnitude of temporary carbon storage in 2100 (Fig. 4). All variables see some reversal of avoided change in the second half of the 21^st^ century when storage SSPs have higher emissions relative to reference SSPs (Fig. 4). For thermosteric sea level rise, total ocean carbon, and global ocean temperature, the end-of-century impacts are determined primarily by the magnitude of storage and show little dependence on the reference SSP (Fig. 4). In contrast, the responses of the AMOC and permafrost carbon are sensitive to the reference SSP. In these cases, particularly for the AMOC response and less so for the permafrost carbon response, the general effect of temporary storage is negatively correlated with end-of-century warming, such that larger climate changes diminish the relative influence of temporary carbon storage (Fig. 4), which demonstrates that the permafrost carbon and AMOC responses to temporary carbon storage are scenario dependent.

The effect of temporary carbon storage on slow-responding variables evolves from 2100 to 2300. By 2300, variables exhibit greater variability in their responses across storage SSPs than in 2100, indicating that their sensitivity to temporary storage becomes increasingly scenario dependent (Fig. 4). This scenario dependence is most pronounced in peak-and-decline scenarios, particularly for permafrost and AMOC responses to temporary carbon storage (Fig. 4). In contrast, a given amount of temporary carbon storage produces similar changes across high-emissions SSPs, which are defined here as SSPs where CO_2_ emissions never reach net-negative and where temperatures rise and then eventually stabilize before 2300 (i.e., SSP2-4.5, SSP4-6.0, SSP3-7.0, and SSP5-8.5; SSPs) (Fig. 4). Among the peak-and-decline SSPs, the largest effects occur in the SSP5-3.4 ensemble, whose reference scenario features a pronounced temperature overshoot, characterized by the most rapid increase, steepest subsequent decline, and highest rate of net-negative CO_2_ emissions (Fig. 1A).

A corresponding version of Fig. 4 that is normalized to the maximum difference between storage SSPs and their respective references in the 21st century is provided in the Supplementary Information (Supplementary Fig. 4). This normalization demonstrates that the climate benefit of temporary storage by 2300 is generally lower than its peak benefit in the 21st century. Ocean temperature and sea-level height retain ~50% of their benefits, whereas total ocean carbon retains only ~10-25%. For permafrost carbon, peak-and-decline SSPs generally retain most of the benefit of temporary storage, while high-emissions SSPs generally see decreased benefit over time. AMOC changes after 2100 are highly variable, with no consistent relationship with storage-years at the year 2300.

### Proportionality between storage-years and climate effects of temporary storage

**Fig. 5:**
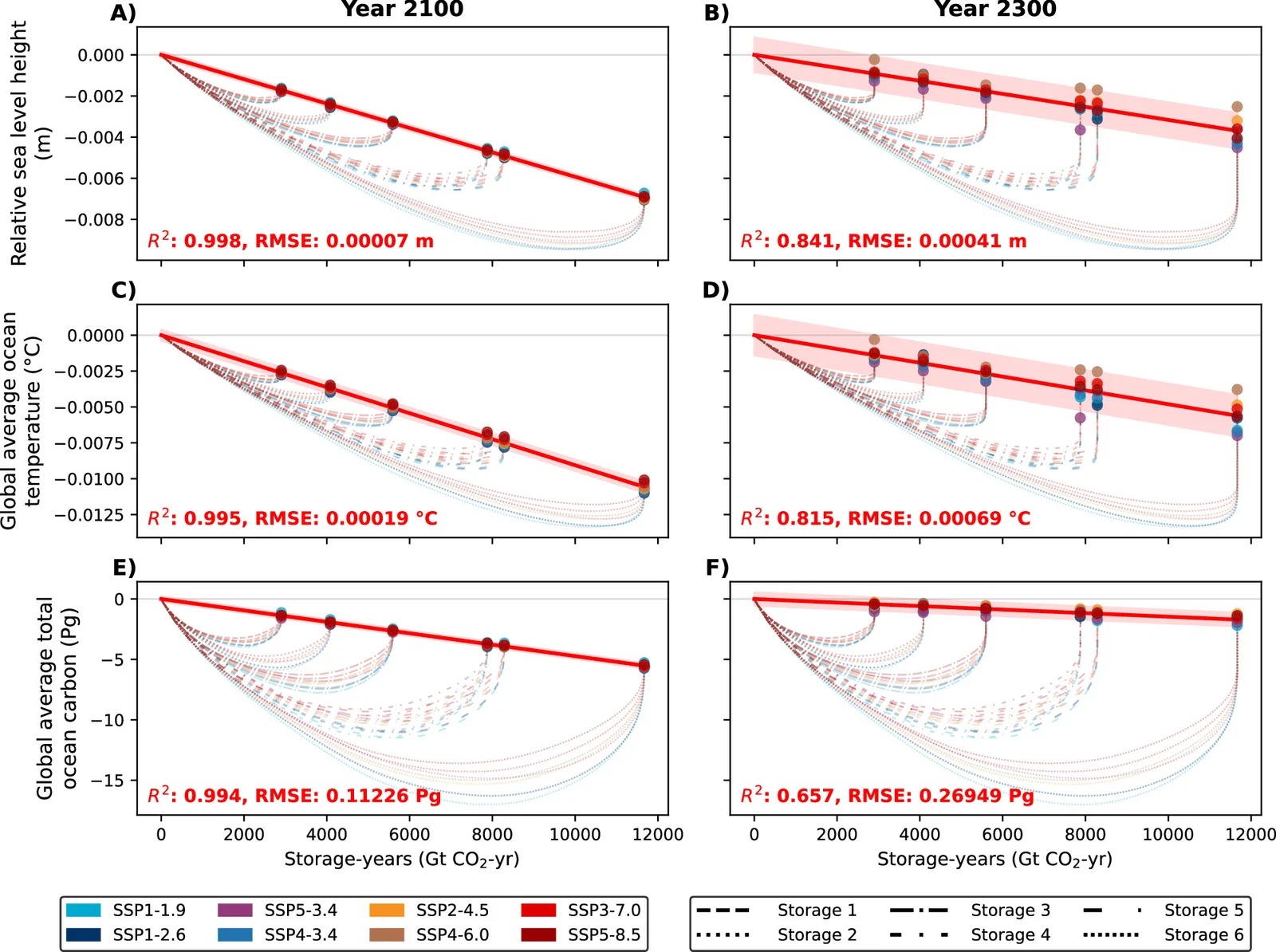
Relationship between storage-years and avoided change in globally averaged ocean variables in storage SSPs relative to their reference SSPs in 2100 and 2300. This figure shows the relationship between storage-years of temporary carbon storage and avoided change to 2100 in the first column, and to 2300 in column two. Rows represent globally averaged ocean variables: (**A**, **B)** thermosteric sea level rise, (**C**, **D)** average ocean temperature, and (**E**, **F)** total ocean carbon. Non-linear dashed/dotted lines represent SSP ensembles (colors) and the six time-limited carbon storage pathways applied to each reference SSP (line types). Colored dots marking 2100 (left column) or 2300 (right column) values for each storage SSP. Solid lines indicate linear relationships between storage-years and avoided change, with regressions forced through the origin to reflect the physical constraint that zero storage yields zero avoided change. Linear fits across all storage SSPs are shown in red, with centered R² values displayed on the left. Red-shaded regions represent 95% confidence intervals for the linear fit. Avoided change per storage-year generally decreases by 2300 as storage SSPs equilibrate with their reference scenarios following carbon re-emission. The R^2^ values decline, and the RMSE and uncertainty increase in 2300, reflecting increased scenario dependence on multi-century timescales. Values for the slope, R^2^, confidence intervals and RMSE can be found in Supplementary Information Table 1.

**Fig. 6:**
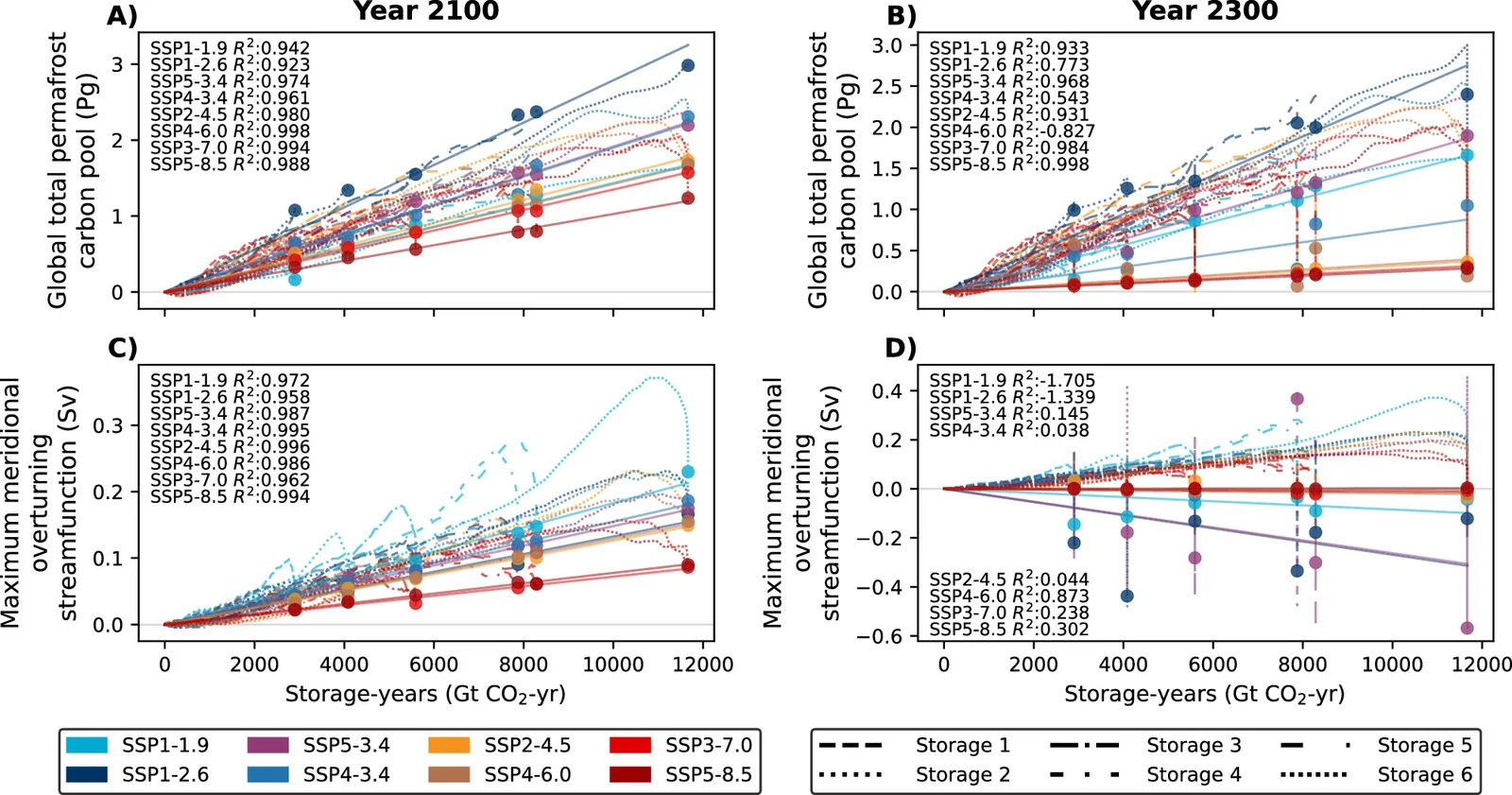
Relationship between storage-years and avoided change of permafrost carbon and AMOC strength in storage SSPs relative to their reference SSPs in 2100 and 2300. This figure shows the relationship between storage-years of temporary carbon storage and avoided change to 2100 in the first column, and to 2300 in column two. Rows represent (**A**, **B**) permafrost carbon response, and (**C**, **D**) the maximum Atlantic meridional overturning (AMOC) response. Non-linear dashed/dotted lines represent SSP ensembles (colors) and the six time-limited carbon storage pathways applied to each reference SSP (line types). Colored dots mark 2100 (right column) or 2300 (left column) values for each storage SSP. Solid lines indicate linear relationships between storage-years and avoided change within each SSP ensemble, with regressions forced through the origin to reflect the physical constraint that zero storage yields zero avoided change. Linear correlations are calculated within each SSP ensemble, with solid lines corresponding to each SSP ensemble and R^2^ values listed within each panel. Both AMOC and permafrost carbon show strong linear correlations within each SSP ensemble in 2100. By 2300, most SSPs retain proportionality between avoided change and storage-years for permafrost carbon, while change in the AMOC loses proportionality with storage-years and becomes highly variable.

Across scenarios, avoided changes in slow-responding variables in 2100 are proportional to temporary carbon storage-years (Figs. 5 & 6). For total ocean carbon, thermosteric sea-level rise, and average ocean temperature, responses in 2100 are proportional to storage-years and are also independent of the reference SSP (Fig. 5A, C, & E). This suggests that, across the durations of storage explored here, avoided end-of-century changes in these variables can be inferred directly from the magnitude of carbon storage measured using storage-years, regardless of the background emissions scenario. By 2300, the magnitude of avoided change per storage-year is reduced as storage SSPs equilibrate with reference SSPs over time. The strength of the linear relationships between storage-years and avoided change is also weaker in 2300 than in 2100 (Fig. 5), indicating that scenario dependence increases as more time elapses following temporary storage for these globally averaged ocean variables. For instance, peak-and-decline SSPs tend to show greater avoided change per storage-year, which sit slightly below the regression line, compared to high-emissions SSPs, which often sit just above the regression line (Fig. 5). For globally averaged ocean variables examined here, the quasi-linear scaling relationship between storage-years and mitigated climate changes holds across the full range of temporary storage magnitudes simulated here, apart from total ocean carbon in 2300, where there is less proportionality with storage-years (see Supplementary Table 1).

Permafrost carbon and the AMOC also scale proportionally with storage-years in 2100, but only within each SSP ensemble, with a different proportionality relationship for different reference SSP scenarios (Fig. 6A & C). This indicates that the relationship between storage-years and variable response is scenario dependent. For the AMOC, this scenario dependence is closely linked to end-of-century warming: while the AMOC remains stronger relative to SSP references in all storage scenarios in 2100, the strength of the storage-year response declines in SSP ensembles with higher end-of-century warming (Fig. 6C). For permafrost carbon, SSPs with greater warming generally show less benefit per storage-year of temporary storage, although this dependence is not explained by end-of-century warming alone. For example, SSP4-3.4 and SSP5-3.4 yield more benefit per storage-year than SSP1-1.9 despite having higher end-of-century warming (Fig. 6A). Instead, the scenario dependence of the permafrost carbon response is likely also influenced by the shape of the temperature pathway, such as differences in peak warming or the rate of warming leading up to 2100.

By 2300, AMOC and permafrost carbon responses to temporary storage diverge. For permafrost carbon, proportionality between avoided change and storage-years remains within most SSP ensembles (Fig. 6B). In high-emissions SSPs, most of the avoided permafrost carbon loss accumulated during the 21st century is negated, leaving only a small residual effect (Fig. 6B). Conversely, in peak-and-decline SSPs, much of the avoided permafrost carbon loss achieved by 2100 persists in 2300 (Fig. 6B). For the AMOC, multi-century changes caused by temporary carbon storage behave differently from the other variables, with proportionality between avoided change and storage-years eroding by 2300 (Fig. 6D). However, there is still a notable difference between the responses to temporary carbon storage in peak-and-decline SSPs relative to high-emissions SSPs. In high-emissions SSPs, there is practically no legacy effect of temporary carbon storage, whereas in peak-and-decline SSPs the AMOC response is highly variable, exhibiting both positive and negative responses across peak-and-decline SSPs and storage pathways (Fig. 6D). As a dynamic flux variable, the AMOC is sensitive to internal variability, which likely drives this scenario divergence and results in greater long-term uncertainty relative to the stock variables examined here.

### Contextualizing the climate benefits of temporary carbon storage

**Fig. 7:**
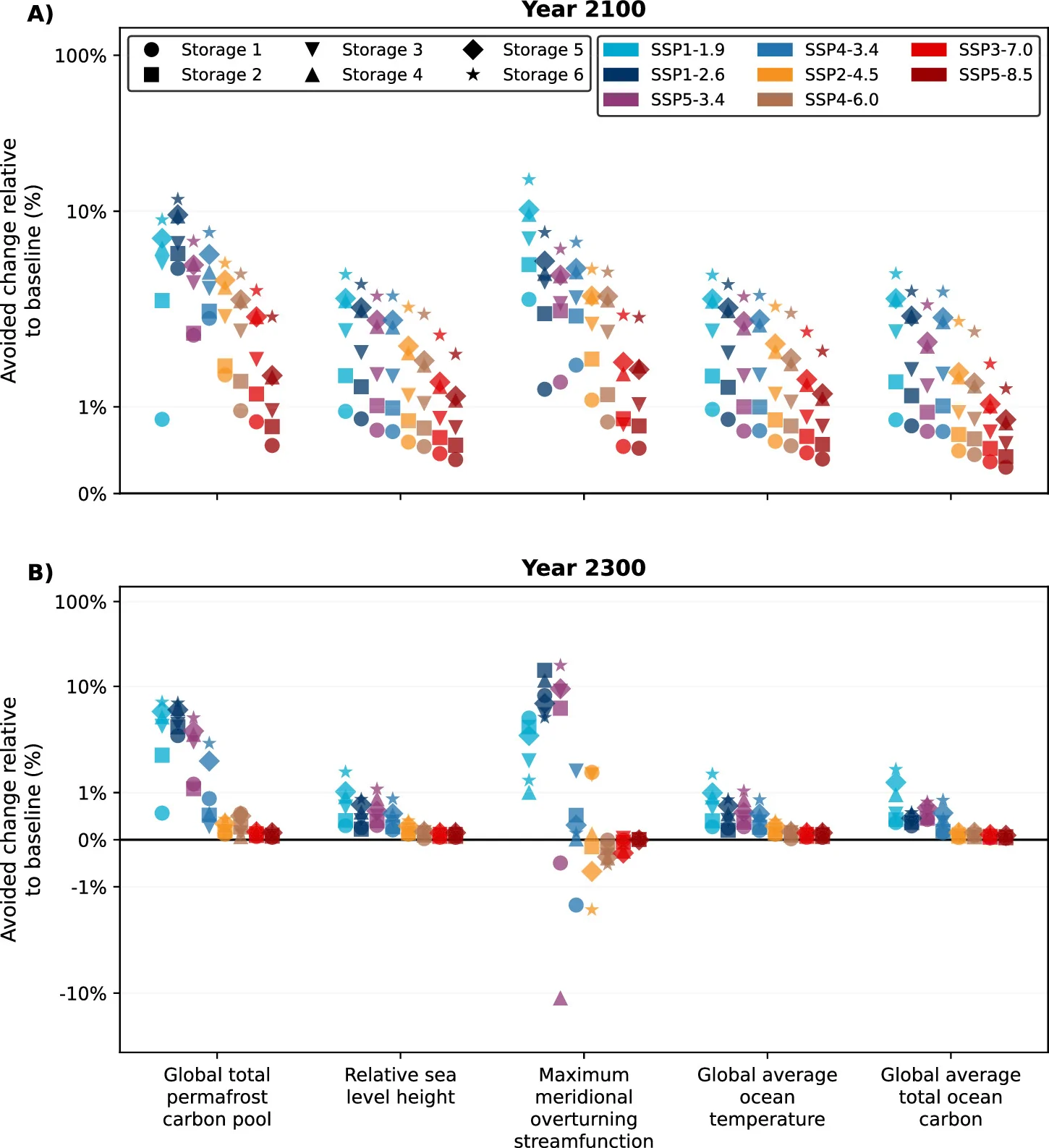
Relative avoided change from temporary carbon storage in 2100 and 2300. This figure shows how avoided changes in storage SSPs compare to total changes in reference SSPs from 2015 to 2100 **A** and from 2015 to 2300 **B** for slow-responding variables. Colors denote SSP ensembles, and marker styles denote the six time-limited carbon storage pathways applied to each reference SSP. By 2100, relative avoided change increases with the magnitude of temporary carbon storage and decreases under reference SSPs with higher end-of-century forcing. Accordingly, the largest relative avoided changes occur in high-storage pathways paired with ambitious mitigation SSPs. This pattern largely persists to 2300 for peak-and-decline SSPs that reach net-negative emissions (SSP1-1.9, SSP1-2.6, SSP4-3.4, SSP5-3.4). In contrast, for high-emissions SSPs that do not reach net-negative CO₂ emissions before 2300 (SSP2-4.5, SSP4-6.0, SSP3-7.0, SSP5-8.5), relative avoided change is close to zero. Maximum Atlantic meridional overturning (AMOC) and permafrost carbon exhibit the highest relative avoided changes in both 2100 and 2300. However, some reference SSP and storage pathway combinations show a negative avoided change in AMOC by 2300, underscoring that the AMOC response to temporary carbon storage is highly variable as a flux variable and can reverse over time, producing more change than in the corresponding reference scenarios.

To contextualize the impact of temporary carbon storage on slow-responding variables, we compare avoided changes in storage SSPs in 2100 (2300) to the total changes in the reference SSPs between 2015, the year when reference scenarios diverge from the historical simulation, and 2100 (2300). Since the impact of temporary carbon storage on slow-responding variables in 2100 scales with storage-years, and since the same six storage pathways are applied to each reference SSP, the relative avoided change in 2100 from temporary carbon storage in storage SSPs generally declines as end-of-century temperature increases. As a result, the relative mitigating effect of temporary carbon storage in 2100 is strongest in ambitious mitigation scenarios (e.g., SSP1-1.9, SSP1-2.6) and weakest in high-emissions scenarios (e.g., SSP5-8.5) (Fig. 7A). Permafrost carbon and the AMOC see the most benefit from temporary carbon storage in 2100, particularly in more ambitious mitigation SSPs where temperatures peak and decline. In SSP ensembles with the highest end-of-century warming, there is less variability in the relative avoided change across variables in 2100 – e.g., SSP5-8.5 storage pathways avoided 0.3% to 2.1% across all variables (Fig. 7A). It is notable that the least ambitious storage pathways (e.g., Storage 1) provide almost no relative benefit by 2100 when paired with the least ambitious SSPs (e.g., SSP5-8.5)(Fig. 7A).

Comparing avoided changes in slow-responding variables to total reference scenario changes from 2015 to 2300 shows that relative avoided change declines by 2300 as storage SSPs gradually equilibrate with their respective reference SSPs (Fig. 7B). In high-emissions SSPs, relative avoided change for all variables in 2300 is close to zero (Fig. 7B). In peak-and-decline SSPs, the relative avoided change in thermosteric sea-level rise, ocean temperature, and total ocean carbon, decreases from a maximum of ~4% in 2100 to a maximum of ~1.5% in 2300 (SSP1-1.9 Storage pathway 6 in Fig. 7A & B). For permafrost carbon, relative avoided change sees only a slight decrease between 2100 and 2300 in peak-and-decline storage SSPs (e.g., SSP1-1.9 Storage 6 sees a decline from 8.8% to 6.5% avoided change). In contrast, high-emissions SSPs see most of the relative avoided change of permafrost carbon loss in 2100 disappear by 2300, indicating that the effect of temporary carbon storage on permafrost carbon persists on multi-century timescales primarily in peak-and-decline scenarios (Fig. 7). For the AMOC, the relative avoided change in peak-and-decline SSPs by 2300 is highly variable, exhibiting both positive and negative responses across scenarios (Fig. 7B). This divergence reflects the breakdown of proportionality between storage-years and the multi-century AMOC response, driven by high flux variability by 2300.

To estimate the difference in climate benefit between non-permanent sequestration and permanent carbon removal, we compare three of our temporary storage pathways against three removal pathways in which the removed carbon is stored permanently until 2300. These removal pathways follow storage pathways two, four, and six in the storage phase, but sequestered carbon remains sequestered until 2300 rather than being re-emitted in the second half of the 21st century (see Methods). By taking the difference of slow-responding variables between permanent removal and temporary storage pathways, we can quantify the potential benefit overestimation that occurs when carbon storage is incorrectly assumed to be permanent.

**Fig. 8:**
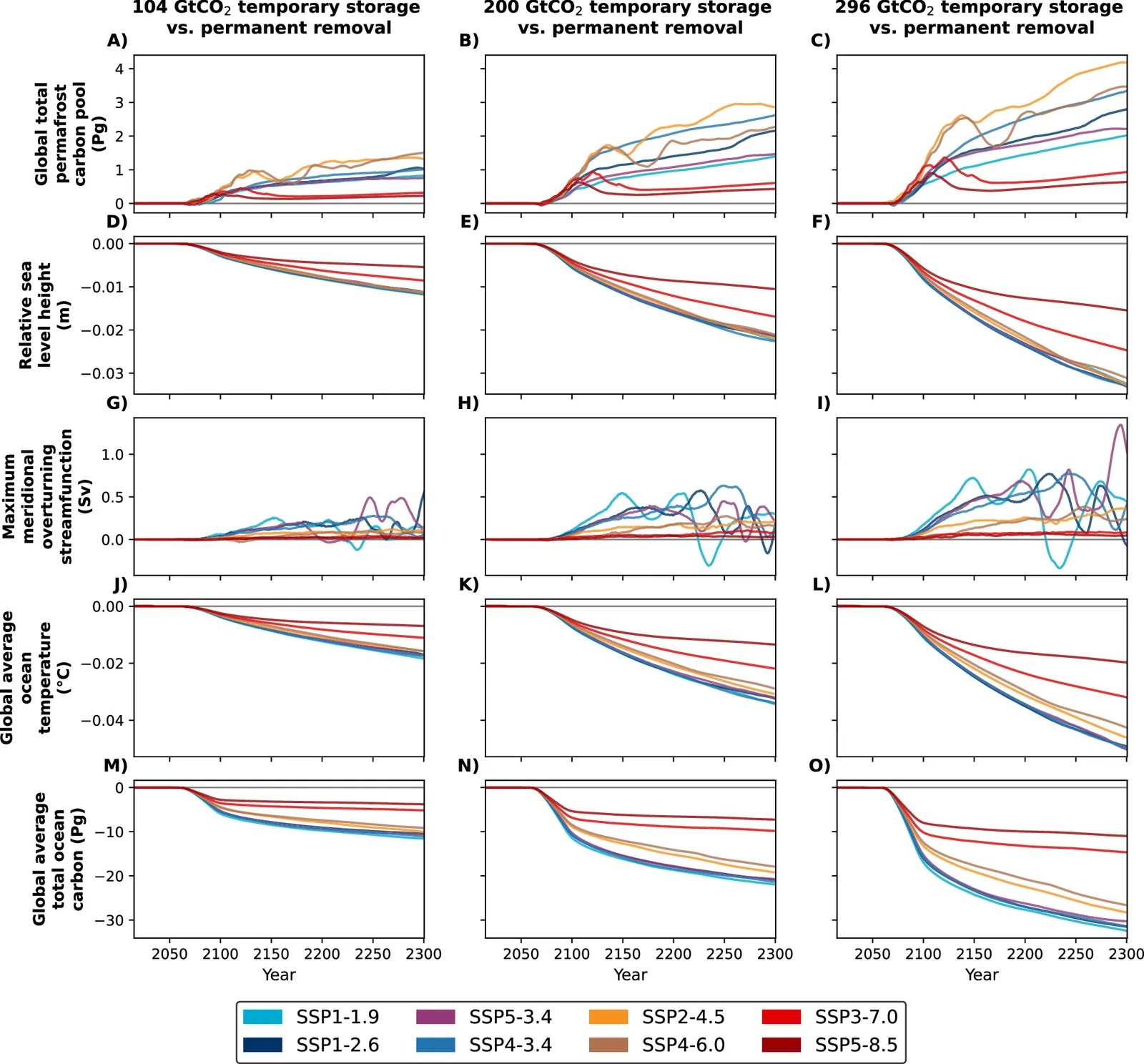
Benefit overestimation of temporary storage relative to permanent removal. Subplots show the difference in climate variables between temporary carbon storage scenarios and corresponding counterfactual scenarios where the same magnitude of carbon is permanently removed, isolating the long-term benefit overestimation of non-permanent sequestration. Rows correspond to individual climate variables: global total permafrost carbon pool (**A**, **B**, **C**), maximum Atlantic meridional overturning (AMOC) (**D**, **E**, **F**), relative sea-level height (**G**, **H**, **I**), global average ocean temperature (**J**, **K**, **L**), and global average total ocean carbon (**M**, **N**, **O**). Columns represent the storage and removal pathways arranged by increasing storage/removal magnitude, listed at the top of each column. Colored lines track the evolution of the difference between temporary storage and permanent removal for each reference SSP. Deviations from zero illustrate the added benefit of permanent removal relative to an equal amount of temporary storage, demonstrating how the overestimation of long-term climate benefits scales with the volume of removal.

Unsurprisingly, permanent removal pathways result in more avoided change than the temporary storage pathways for all SSPs and all variables. For thermosteric sea-level rise, ocean temperature, and total ocean carbon (Fig. 8), permanent removal has an increasing benefit over time relative to non-permanent storage. The maximum added benefits from permanent removal by 2300 are 3.3 cm of avoided thermosteric sea-level rise, 0.05 °C of avoided ocean heating, and 32 Pg C of avoided ocean carbon uptake (Fig. 8I, L, O). The added benefit of permanent removal has a clear scenario dependence – the SSPs with the highest end-of-century warming (i.e., SSP5-8.5, SSP3-7.0) see the climate benefit of permanent removal accumulate more slowly. For permafrost carbon, permanent removal avoids more permafrost carbon loss relative to temporary storage. However, peak-and-decline SSPs see avoided permafrost carbon loss accumulate throughout the simulations, while high-emissions SSPs see the benefit of permanent removal decrease briefly as temperatures stabilize before continuing to accumulate (Fig. 8A–C). By 2300, permanent carbon removal can avoid up to 4.2 Pg C permafrost carbon loss relative to temporary carbon removal (Fig. 8C). Finally, the AMOC response to permanent removal pathways sees brief periods (years to decades) of less climate benefit than temporary pathways due to high AMOC variability in SSP1-1.9, SSP1-2.6, SSP5-3.4, and SSP4-6.0 (Fig. 8D–F), but otherwise permanent removal pathways lead to less AMOC slowdown relative to temporary carbon storage pathways.

## Discussion

The potential for carbon storage to only be temporary makes it increasingly important to understand the climate impacts of temporary carbon storage so that different means of removal, particularly those that are nature-based, and their associated risks can be fairly weighed against other mitigation options. Our results show that the biogeochemical effect of temporary carbon storage can deliver measurable climate benefits that persist beyond the lifetime of the stored carbon. For temperature, temporary storage can lower peak warming when combined with ambitious mitigation, which previous work shows would reduce the risk of triggering climate tipping points^40^. For the slow-responding variables examined here, temporary carbon storage reduces impacts by the end of the century, with avoided changes by 2100 scaling proportionally with the magnitude and duration of storage, expressed in storage-years. For thermosteric sea level rise, ocean temperature, and total ocean carbon, this proportionality is independent of the reference SSP. In contrast, the proportionality in 2100 between storage-years and avoided change in the AMOC and permafrost carbon depends strongly on the reference scenario, with more ambitious mitigation SSPs generally showing the largest avoided changes per storage-year. Because these scenario-dependent proportionalities are calculated within each SSP ensemble, each regression relies on only six data points. The proportionality relationships for AMOC and permafrost carbon are therefore inherently less robust than the scenario-independent proportionality derived from the fully aggregated multi-scenario dataset.

Our results also demonstrate that by 2300, the benefits of temporary carbon storage on slow-responding variables decrease, particularly in high-emissions SSPs, where temperature continues to increase after 2100. Furthermore, the proportionality between storage-years and the climate response weakens by 2300, exhibiting clear divergence among slow-responding variables. Thermosteric sea-level rise, ocean temperature, and total ocean carbon retain scenario-independent proportionality to storage-years, whereas permafrost carbon only retains proportionality across temporary storage amounts within individual SSP groups (scenario-dependent proportionality). AMOC changes show high multi-century flux variability and therefore do not retain their proportionality with storage-year amounts on multi-centennial timescales.

The proportionality between storage-years and the impact of temporary carbon storage observed here depends on the physical characteristics of the variables examined. Here, this framework generally holds for slow-responding globally averaged stock variables because these components possess high physical inertia. Aside from the AMOC, which is the only flux variable in our analysis, these variables act as low-pass filters that integrate the cumulative radiative forcing anomaly over time, smoothing out variations in the precise timing of carbon sequestration and release. Interdependencies among these variables can also shape long-term outcomes, as demonstrated by recent evidence that AMOC weakening can partially offset permafrost carbon loss in overshoot scenarios via AMOC driven regional cooling in the North Atlantic^41^. While our analysis focuses on global-scale trends across a broad scenario space, these AMOC-permafrost interdependencies underscore the importance of future spatial analyses, which may help explain the increasing scenario dependence observed toward 2300.

This proportionality may also be subject to the specific characteristics of the UVic-ESCM. A key structural characteristic of the UVic-ESCM is its single-layer atmosphere and prescribed atmospheric circulation, which prevent the model from generating significant atmospheric variability. The response of a full-complexity model would be additionally affected by unforced decadal atmospheric variability, which could introduce greater scenario dependence. Another consideration is that the proportionality found here is a function of the UVic-ESCM’s TCRE given that the fundamental proportionality between carbon storage-years and degree-years of avoided warming arises directly from TCRE linearity; a model with a different TCRE value should simulate a different magnitude of proportionality during the period of active storage-years (i.e., up to 2100). Additionally, differences in ZEC values among models would change the relationship between storage-years and changes occurring after storage-years stop accumulating (i.e., from 2100 to 2300). We expect that our conclusion of proportionality at the year 2100 would hold, given the consistent linearity of the TCRE relationship among models. Beyond 2100, however, climate responses would become more sensitive to model-dependent ZEC values and to the possibility of model-dependent TCRE asymmetry between emissions and removals, the latter being relevant primarily in scenarios reaching net-negative CO_2_. Despite these potential inter-model differences, elements of the proportionality found here are consistent with existing studies. For instance, a proportional relationship between permafrost carbon loss and time-integrated warming has been shown in a full Earth system model, intermediate-complexity models, and a land surface model^25,41^. Future multi-model intercomparisons are an important next step to verify whether this storage-year proportionality remains robust for other variables across different climate model structures and complexities. Additionally, exploring the spatial patterns of these responses in other models is an important priority, especially since UVic-ESCM’s single-layer atmosphere and prescribed circulation limit its ability to draw detailed, spatially explicit conclusions.

Any real-world interpretation of these findings must also consider the design of our study. Most notably, our simulations isolate the biogeochemical response to temporary storage and do not account for the additional biogeophysical, non-local, or non-CO₂ effects that can be associated with carbon removal^42^. For instance, if temporary storage is achieved via high-latitude reforestation or afforestation, localized biophysical feedbacks, such as reduced surface albedo, could counteract biogeochemical cooling and lower the slope of the storage-years relationship with avoided inertial climate changes^6,43^. While these findings are particularly relevant to the biogeochemical effects of nature-based carbon removal and storage, given its uncertain durability, it is important to highlight that our results reflect the biogeochemical effects of any temporary carbon removal and storage. Furthermore, the storage rates applied here are highly idealized, which is an important consideration given that the efficacy of temporary storage appears to be partially contingent on the timing of sequestration and re-emission relative to peak warming. For instance, if temporary storage is re-emitted prior to net-zero CO_2_, as opposed to after net-zero CO_2_ as in the peak-and-decline SSPs in this study, storage may merely shift the peak’s timing without reducing its magnitude. Therefore, while this work confirms that the proportionality between storage-years and avoided inertial climate changes holds across a wide range of storage magnitudes, a wider variety of temporal profiles of temporary carbon storage, in addition to the two temporal profiles simulated here, would have to be used to explore how well this proportionality holds across varying durations of temporary carbon storage.

Despite these complexities, the robust relationship we identify between carbon storage-years and avoided changes in some inertial variables provides a potential mechanism to value non-permanent sequestration across the storage magnitudes we explore here. Conservative estimates of countries’ Nationally Determined Contributions under the Paris Agreement indicate that approximately one billion hectares of land have been pledged to be used for carbon dioxide removal or storage projects by 2060^44^. Our results indicate that if these pledges are fulfilled, but the carbon is only temporarily stored, there would still be measurable twenty-first-century climate benefits from the biogeochemical impacts of carbon storage. However, the actual net benefit of these real-world pledges, particularly those involving nature-based means, remains highly contingent on biogeophysical effects which may counteract this biogeochemical cooling effect. By providing a measure of avoided biogeochemically driven change in slow-responding variables, the metric of storage-years offers a framework to weigh these benefits of temporary storage against the benefits of more permanent mitigation options, such as sustained reductions of fossil CO_2_ emissions or durable geologic storage.

Our results also demonstrate that the climate benefits of temporary carbon storage increase when paired with ambitious emissions reductions but diminish substantially in scenarios where emissions continue to rise. For example, we show that the relative avoided change caused by temporary carbon storage depends strongly on the magnitude and duration of storage – in SSP1-1.9, the relative avoided change of permafrost carbon in 2100 (2300) for Storage pathway 1 is 0.9% (0.6%), while it is 8.8% (6.5%) in Storage pathway 6 (Fig. 7). This implies that in a world where global CO₂ emissions do not decline or decline slowly and where the highest estimated rates of carbon storage are not achieved, the relative avoided change caused by temporary storage will be limited. Our results also demonstrate that the false assumption of permanence can lead to an overestimation of the benefit of any temporarily stored carbon for inertial climate variables. Given these findings, we echo the conclusions of other studies^5,9,10^ and argue that any carbon storage that may only provide temporary storage, such as nature-based means, should be regarded as a complement to, rather than a substitute for, sustained fossil CO₂ emissions reductions and the deployment of more durable carbon removal approaches.

## Methods

### Scenario design

We developed six temporary carbon storage pathways for each reference SSP. These are based on three hypothetical maximum CO_2_ storage rates and two distinct pathways of storage and re-emission. Maximum rates of carbon removal and re-emission ranged from ±3.64 Gt CO_2_ yr^−1^, which is consistent with a conservative estimate of afforestation and reforestation potential^45^, to ±10.4 Gt CO_2_ yr^−1^, which is consistent with an optimistic estimate of cost-effective nature-based avoided emissions and removal from all sources^46^. In both storage and re-emission pathways, CO₂ is removed, or emissions are avoided, and stored until 2056 and then re-emitted in equal cumulative amounts between 2057 and 2100, so that the total negative emissions are balanced by positive emissions. The first pathway consists of five phases: (1) a linear decrease in emissions from zero in 2020 to the maximum removal rate in 2030; (2) a linear increase back to zero by 2056; (3) a 12-year linear increase to the maximum rate of positive emissions; (4) 20 years of constant positive emissions at that maximum rate; and (5) a 12-year linear decline back to zero. The second pathway is identical except that, instead of emissions linearly increasing to zero from 2030 to 2056 (step 2), the maximum storage rate is sustained until 2050 before increasing rapidly to zero between 2050 and 2056. The two distinct storage and re-emission pathways represent varying temporal patterns of storage, which result in different cumulative removals and re-emissions. Using two distinct temporal trajectories – one with a linear ramp and one with sustained maximum removal – allows us to test the robustness of the storage-year proportionality framework across pathways with different timing of sequestration and re-emission. Each of the three storage rates is paired with both pathways, yielding six storage pathways in total. When applied to the eight reference SSPs, these scenarios generate 48 temporary carbon storage pathways. Given that cumulative CO_2_ emissions reductions occurring in the first half of the 21^st^ century are balanced by additions of CO_2_ emissions in the second half of the century, each of the storage pathways applied to the SSPs have identical cumulative CO_2_ emissions relative to their reference SSP from 2100 until the end of the simulations in 2300.

In addition to the temporary carbon storage pathways, we also applied three permanent carbon removal pathways to each reference SSP. The permanent removal pathways follow storage and re-emission pathways two, four, and six – that is, the second temporal pattern of storage at the three different emission rates – except that the carbon removed up until 2056 is not re-emitted in the second half of the 21^st^ century.

To ensure carbon storage pathways were applied to a consistent baseline, we standardized land-use CO_2_ emissions across all SSP scenarios. Ambitious mitigation SSPs (e.g., SSP1-1.9 or SSP1-2.6) already include significant land-based carbon removal. Therefore, adding large additional storage perturbations to these SSPs would likely produce carbon removal pathways that are unrealistic from technical, social, and/or economic perspectives. In recognition of this, we replaced the land-use CO_2_ emissions of every SSP with values from the SSP3-7.0 scenario because it is the only SSP where land-use CO_2_ emissions remain positive throughout the 21st century. This provides a standardized reference scenario free of large amounts of pre-existing carbon dioxide removal, ensuring that experimental storage pathways are not stacked onto already ambitious land-sink assumptions. Because our simulations are emissions-driven and keep land cover fixed rather than modeling spatially explicit land-use change, this standardization serves purely to explore global emissions pathways against an established positive land-use emissions background. Aside from SSP3-7.0, this standardization alters the total cumulative CO_2_ emissions and resulting 2100 temperatures for all reference SSPs. All other forcings (fossil fuel emissions, non-CO_2_ greenhouse gases, and aerosols) remain specific to their original SSP definitions.

### Model

For our simulations, we used the UVic-ESCM version 2.10. The UVic-ESCM is an intermediate-complexity coupled climate-carbon cycle model designed to simulate interactions between the atmosphere, ocean, terrestrial, and sea ice systems at a resolution of 3.8° of longitude and 1.8° of latitude^26,27^. The atmospheric component is a simplified two-dimensional, single-layer energy–moisture balance model that captures large-scale heat and moisture fluxes without simulating detailed atmospheric circulation, thereby improving computational efficiency. The ocean is represented by a three-dimensional ocean general circulation model with 19 vertical layers, a marine sediment model, as well as a marine biogeochemical model which represents the cycling of inorganic and organic carbon, nitrogen, phosphorus, and oxygen. The terrestrial system includes a land surface model simulating the physical exchanges of energy, heat, and moisture with the atmosphere, a dynamic vegetation model representing five plant functional types, a multi-layer soil model that captures freeze–thaw processes and carbon cycling, and a permafrost module. The relatively low computational cost of the UVic-ESCM enables the simulation of large ensembles and of multi-century experiments while including key processes governing global temperature, ocean circulation, and carbon fluxes, which makes it well-suited for our study. The model has been widely applied to investigate scenarios of anthropogenic emissions, carbon removal, and long-term climate response^6,37,47–50^.

### Simulation design

The eight reference SSP simulations were emissions-driven. Forcings included fossil and land-use change CO₂ emissions, aggregated non-CO₂ greenhouse gas radiative forcing, and aerosol emissions from Mathesius et al.^51^. The 48 storage SSP simulations are identical to their reference counterparts, except that temporary storage (and subsequent re-emissions) were subtracted from (and added to) prescribed land-use CO₂ emissions. By modulating prescribed land-use CO_2_ emissions rather than explicitly including the land-use and land-cover changes that would accompany many forms of nature-based carbon removal, our results reflect the biogeochemical effect of temporary carbon storage but not biogeophysical effects such as altered surface albedo or cloud cover^43,52,53^. Solar and volcanic forcing varied transiently in the historical simulations but were held constant in both reference and storage SSP experiments. Land cover was prescribed and held fixed throughout all historical and future simulations.

## Supplementary information

SUPPLEMENTARY INFO

Transparent Peer Review file

## Data Availability

The data that support the findings of this study have been deposited in a Zenodo repository^54^ and can be found at https://doi.org/10.5281/zenodo.22691534.

## Code availability

The UVic-ESCM 2.10 model code can be found at http://terra.seos.uvic.ca/model/2.10. The analysis code used in this study has been published in a Zenodo repository^54^ and can be found at https://doi.org/10.5281/zenodo.22691534.

## References

[1] Government of Canada. 2 Billion Trees Commitment. (2025).

[2] Smith, B. Microsoft will be carbon negative by 2030. Official Microsoft Blog (2020).

[3] Anderegg, W. R. L. et al. Climate-driven risks to the climate mitigation potential of forests. *Science* **368**, 1979 (2020). 10.1126/science.aaz7005 32554569

[4] McDowell, N. G. et al. Pervasive shifts in forest dynamics in a changing world. *Science* **368**, 1979 (2020). 10.1126/science.aaz9463 32467364

[5] Brunner, C., Hausfather, Z. & Knutti, R. Durability of carbon dioxide removal is critical for Paris climate goals. *Commun. Earth Environ.* **5**, 645 (2024).

[6] Matthews, H. D. et al. Temporary nature-based carbon removal can lower peak warming in a well-below 2 °C scenario. *Commun. Earth Environ.* **3**, 65 (2022).

[7] Eby, M. et al. Lifetime of anthropogenic climate change: Millennial time scales of potential CO2 and surface temperature perturbations. *J. Clim.* **22**, 2501–2511 (2009).

[8] Solomon, S., Plattner, G.-K., Knutti, R. & Friedlingstein, P. Irreversible climate change due to carbon dioxide emissions. *Proc. Natl. Acad. Sci. USA* **106**, 1704–1709 (2009). 10.1073/pnas.0812721106 PMC2632717 19179281

[9] Allen, M. R. et al. Geological Net Zero and the need for disaggregated accounting for carbon sinks. *Nature* **638**, 343–350 (2025). 10.1038/s41586-024-08326-8 39557072

[10] Seddon, N. Harnessing the potential of nature-based solutions for mitigating and adapting to climate change. *Science* **376**, 1410–1416 (2022). 10.1126/science.abn9668 35737796

[11] Moura Costa, P. & Wilson, C. An equivalence factor between CO2 avoided emissions and sequestration – description and applications in forestry. *Mitig. Adapt. Strateg. Glob. Chang.* **5**, 51–60 (2000).

[12] IPCC. IPCC Special Report: Land Use, Land-Use Change, and Forestry. Summary for Policymakers (2000).

[13] Lashof, D. & Hare, B. The role of biotic carbon stocks in stabilizing greenhouse gas concentrations at safe levels. *Environ. Sci. Policy* **2**, 101–109 (1999).

[14] British Standards Institution. *Publicly Available Specification PAS 2050*:*2008*. *Specification for the Assessment of the Life Cycle Greenhouse Gas Emissions of Goods and Services*. October (2008).

[15] Brandão, M. et al. Key issues and options in accounting for carbon sequestration and temporary storage in life cycle assessment and carbon footprinting. *Int. J. Life Cycle Assess.* **18**, 230–240 (2013).

[16] Levasseur, A., Lesage, P., Margni, M., Brandão, M. & Samson, R. Assessing temporary carbon sequestration and storage projects through land use, land-use change and forestry: comparison of dynamic life cycle assessment with ton-year approaches. *Clim. Change* **115**, 759–776 (2012).

[17] Müller-Wenk, R. & Brandão, M. Climatic impact of land use in LCA—carbon transfers between vegetation/soil and air. *Int. J. Life Cycle Assess.* **15**, 172–182 (2010).

[18] Brandão, M., Kirschbaum, M. U. F., Cowie, A. L. & Hjuler, S. V. Quantifying the climate change effects of bioenergy systems: Comparison of 15 impact assessment methods. *GCB Bioenergy* **11**, 727–743 (2019).

[19] Sierra, C. A., Crow, S. E., Heimann, M., Metzler, H. & Schulze, E.-D. The climate benefit of carbon sequestration. *Biogeosciences* **18**, 1029–1048 (2021).

[20] Metzler, H., Launiainen, S. & Vico, G. Amount of carbon fixed, transit time and fate of harvested wood products define the climate change mitigation potential of boreal forest management—A model analysis. *Ecol. Modell.* **491**, 110694 (2024).

[21] Minasny, B. & McBratney, A. B. Soil carbon tonne-year accounting: Crediting the additional time-integrated amount of carbon captured in soil. *Soil Security* **15**, 100153 (2024).

[22] Alexander, J. R., Gamble, J. D. & Venterea, R. T. Stock change accounting overestimates the potential climate benefit of soil carbon storage. *Soil Sci. Soc. Am. J.* **88**, 745–752 (2024).

[23] Matthews, H. D., Zickfeld, K., Koch, A. & Luers, A. Accounting for the climate benefit of temporary carbon storage in nature. *Nat. Commun.* **14**, 5485 (2023). 10.1038/s41467-023-41242-5 PMC10485027 37679349

[24] Sierra, C. Integrating time in definitions of carbon sequestration and greenhouse gas removals and reversals. *R. Soc. Open Sci*. **12**, 10.1098/rsos.242095 (2025). PMC12133336 40469661

[25] Dickau, M., Zickfeld, K. & Matthews, H. D. Irreversible climate changes driven by degree-years of temperature overshoot. *Commun. Earth Environ*. 10.1038/s43247-026-03761-z (2026). PMC13630551 42827589

[26] Weaver, A. J. et al. The UVic earth system climate model: Model description, climatology, and applications to past, present and future climates. *Atmosphere-Ocean* **39**, 361–428 (2001).

[27] Mengis, N. et al. Evaluation of the University of Victoria Earth System Climate Model version 2.10 (UVic ESCM 2.10). *Geosci. Model Dev.* **13**, 4183–4204 (2020).

[28] Kim, S.-K. et al. Widespread irreversible changes in surface temperature and precipitation in response to CO2 forcing. *Nat. Clim. Chang.* **12**, 834–840 (2022).

[29] Pfleiderer, P., Schleussner, C.-F. & Sillmann, J. Limited reversal of regional climate signals in overshoot scenarios. *Environ. Res.: Clim.* **3**, 015005 (2024).

[30] Canadell, J. G. et al. Global Carbon and other Biogeochemical Cycles and Feedbacks. in *Climate Change 2021: The Physical Science Basis. Contribution of Working Group I to the Sixth**Assessment Report of the Intergovernmental Panel on Climate Change* (eds. Masson-Delmotte, V. et al.) (Cambridge University Press, 2021).

[31] Allen, M. R. Warming caused by cumulative carbon emissions towards the trillionth tonne. *Nature* **458**, 1163–1166 (2009). 10.1038/nature08019 19407800

[32] Meinshausen, M. et al. Greenhouse-gas emission targets for limiting global warming to 2 °C. *Nature* **458**, 1158–1162 (2009). 10.1038/nature08017 19407799

[33] Zickfeld, K., Eby, M., Damon Matthews, H. & Weaver, A. J. Setting cumulative emissions targets to reduce the risk of dangerous climate change. *Proc. Natl. Acad. Sci. Usa.* **106**, 16129–16134 (2009). 10.1073/pnas.0805800106 PMC2752604 19706489

[34] Matthews, H. D., Gillett, N. P., Stott, P. A. & Zickfeld, K. The proportionality of global warming to cumulative carbon emissions. *Nature* **459**, 829–832 (2009). 10.1038/nature08047 19516338

[35] Caldeira, K. & Kasting, J. F. Insensitivity of global warming potentials to carbon dioxide emission scenarios. *Nature* **366**, 251–253 (1993).

[36] Matthews, H. D. & Caldeira, K. Stabilizing climate requires near-zero emissions. *Geophys. Res. Lett*. **35** 10.1029/2007GL032388 (2008).

[37] Zickfeld, K., MacDougall, A. H. & Damon Matthews, H. On the proportionality between global temperature change and cumulative CO2 emissions during periods of net negative CO2 emissions. *Environ. Res. Lett.* **11**, 055006 (2016).

[38] Koven, C. D. et al. Multi-century dynamics of the climate and carbon cycle under both high and net negative emissions scenarios. *Earth Syst. Dyn.* **13**, 885–909 (2022).

[39] MacDougall, A. H. et al. Is there warming in the pipeline? A multi-model analysis of the Zero Emissions Commitment from CO2. *Biogeosciences* **17**, 2987–3016 (2020).

[40] Ritchie, P. D. L., Huntingford, C. & Cox, P. M. ESD Ideas: Climate tipping is not instantaneous – the duration of an overshoot matters. *Earth Syst. Dyn.* **16**, 1523–1526 (2025).

[41] Steinert, N. J. et al. Permafrost carbon release scales linearly with overshoot warming mediated by AMOC tipping. *Nat. Commun.* **17**, 6395 (2026). 10.1038/s41467-026-73612-0 PMC13376509 42463497

[42] Zickfeld, K. et al. Net-zero approaches must consider Earth system impacts to achieve climate goals. *Nat. Clim. Chang.* **13**, 1298–1305 (2023).

[43] Betts, R. A. Offset of the potential carbon sink from boreal forestation by decreases in surface albedo. *Nature* **408**, 187–190 (2000). 10.1038/35041545 11089969

[44] Dooley, K., Christiansen, K. L., Lund, J. F., Carton, W. & Self, A. Over-reliance on land for carbon dioxide removal in net-zero climate pledges. *Nat. Commun.* **15**, 9118 (2024). 10.1038/s41467-024-53466-0 PMC11496795 39438491

[45] Roe, S. et al. Contribution of the land sector to a 1.5 °C world. *Nat. Clim. Change* **9**, 817–828 (2019).

[46] Griscom, B. W. et al. Natural climate solutions. *Proc. Natl. Acad. Sci. USA* **114**, 11645–11650 (2017). 10.1073/pnas.1710465114 PMC5676916 29078344

[47] MacDougall, A. H. Reversing climate warming by artificial atmospheric carbon-dioxide removal: Can a Holocene-like climate be restored? *Geophys. Res. Lett.* **40**, 5480–5485 (2013).

[48] MacDougall, A. H., Zickfeld, K., Knutti, R. & Matthews, H. D. Sensitivity of carbon budgets to permafrost carbon feedbacks and non-CO2 forcings. *Environ. Res. Lett.* **10**, 125003 (2015).

[49] Matthews, H. D. & Zickfeld, K. Climate response to zero emissions of greenhouse gases and aerosols. *Nat. Clim. Change* **2**, 338–341 (2012).

[50] Zickfeld, K., Azevedo, D., Mathesius, S. & Matthews, H. D. Asymmetry in the climate–carbon cycle response to positive and negative CO2 emissions. *Nat. Clim. Chang.* **11**, 613–617 (2021).

[51] Mathesius, S. et al. CMIP6 scenarios’ radiative forcing of non-CO2 greenhouse gases and aerosols for UVic ESCM simulations (1850-2500). *Zenodo*. 10.5281/zenodo.11061151 (2024).

[52] Jayakrishnan, K. U. & Bala, G. A comparison of the climate and carbon cycle effects of carbon removal by afforestation and an equivalent reduction in fossil fuel emissions. *Biogeosciences* **20**, 1863–1877 (2023).

[53] Bala, G. et al. Combined climate and carbon-cycle effects of large-scale deforestation. *Proc. Natl. Acad. Sci. USA* **104**, 6550–6555 (2007). 10.1073/pnas.0608998104 PMC1871823 17420463

[54] Dickau, M. Temporary carbon storage can mitigate slow-responding climate changes. *Zenodo*. 10.5281/zenodo.22691534 (2026). PMC13759493 43020786

