# Supplementary material for: Temporary carbon storage can mitigate slow-responding climate changes
Source: Nat Commun. 2026 Oct 16;17:11671. doi: 10.1038/s41467-026-78620-8 (PMC13759493; doi:10.1038/s41467-026-78620-8)
Supplement: Supplementary file 1 [file 41467_2026_78620_MOESM1_ESM.pdf]

Supplementary Information For:

Temporary carbon storage can mitigate slow-responding climate changes

Mitchell Dickau[1*] and H. Damon Matthews[1]

[1]Department of Geography, Planning and Environment, Concordia University, Montréal, Quebec, Canada

* Corresponding author at: Mitchell.dickau@mail.concordia.ca

This file includes:

- Supplementary Table 1
- Supplementary Figures 1 to 4

time	variable	R2	m	ci	RMSE
2100.5	Relative sea level height	0.998	-5.93E-07	[-5.96e-07, -5.9e-07]	7.31E-05
2100.5	Global average ocean temperature	0.995	-9.06E-07	[-9.14e-07, -8.99e-07]	0.00019
2100.5	Global average total ocean carbon	0.994	-0.000473	[-0.000477, -0.000468]	0.112
2300.5	Relative sea level height	0.841	-3.17E-07	[-3.33e-07, -3e-07]	0.000412
2300.5	Global average ocean temperature	0.815	-4.81E-07	[-5.09e-07, -4.53e-07]	0.000692
2300.5	Global average total ocean carbon	0.657	-0.000146	[-0.000157, -0.000135]	0.269

Supplementary Table 1 – Regression summary table. *This table shows the R2, regression slope (m), 95% confidence intervals (ci), and RMSE for the regressions of variables with scenario independent proportionality with storage- years found in Figure 5 of the main manuscript. AMOC and permafrost carbon are not included because their scenario-dependent proportionalities are calculated within each SSP ensemble, thus each regression relies on only six data points. These relationships are therefore inherently less robust than the scenario-independent proportionality derived from the fully aggregated multi-scenario dataset.*

SUPPLEMENTARY FIGURES 1-4:

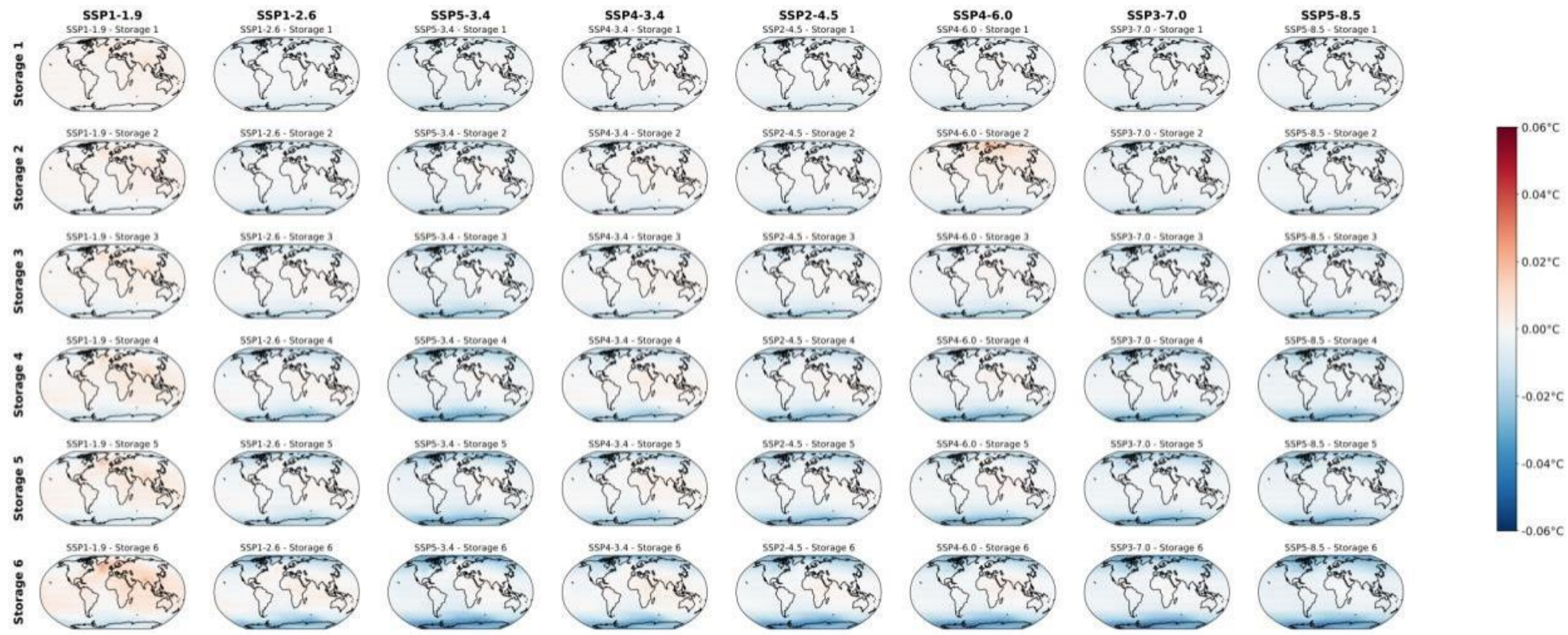

Supplementary Figure 1 – Average difference in surface air temperature between storage SSPs and reference SSPs between 2100 to 2120. *Shown is the 20-year mean difference in surface air temperature (SAT) between storage and reference SSPs (i.e., storage SAT subtracted by reference SAT) for the period 2100–2120, corresponding to the two decades following the re-emission of all temporarily stored carbon. Columns indicate reference SSPs (listed above), and rows indicate the six time-limited carbon storage pathways applied to each reference SSP (listed to the left). In general, storage SSPs remain cooler at high latitudes and exhibit equal or slightly warmer temperature in equatorial and mid-latitude regions relative to their reference SSPs. This regional pattern generally strengthens with increasing magnitude of temporary carbon storage across all reference SSPs.*

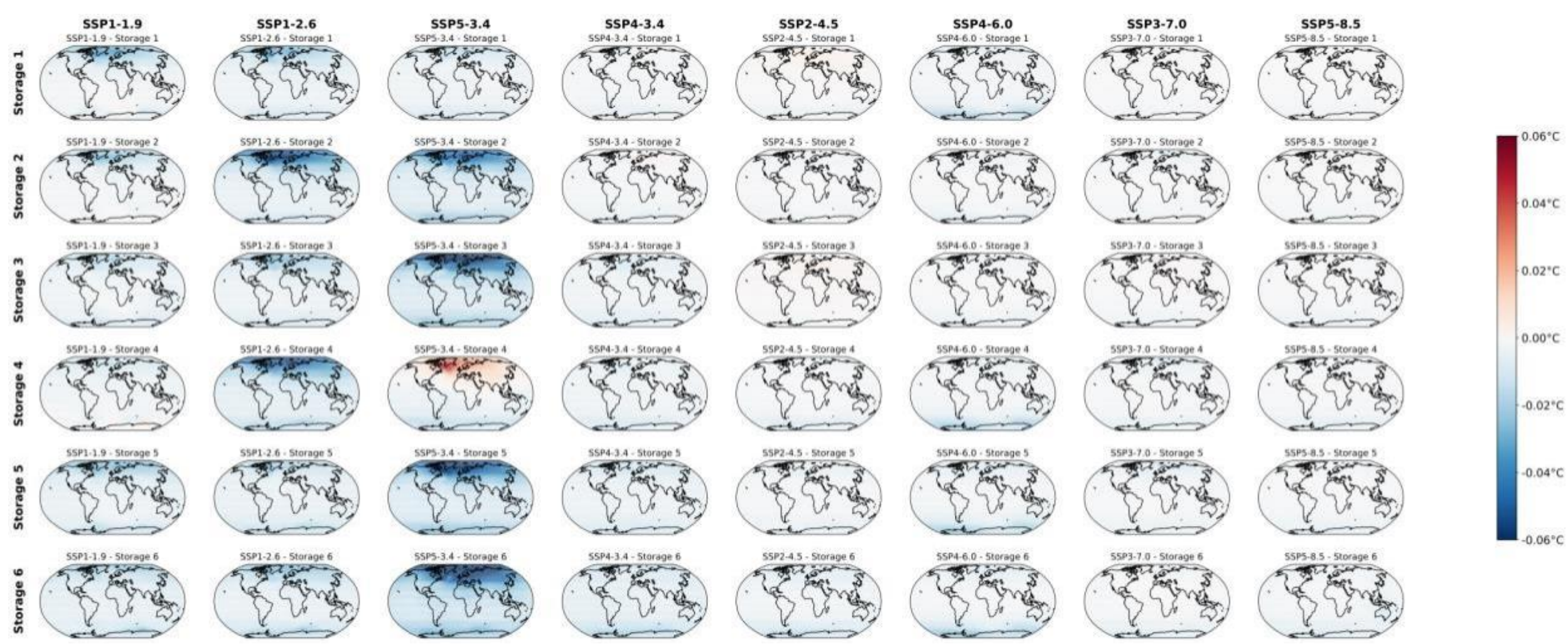

*S**upplementary Figure 2 – Average difference in surface air temperature between storage SSPs and reference SSPs between 2280 to 2300.** Shown is the 20-year mean difference in surface air temperature (SAT) between storage and reference SSPs (i.e., storage SAT subtracted by reference SAT) for the period 2280–2300. Columns indicate reference SSPs (listed above), and rows indicate the six time-limited carbon storage pathways applied to each reference SSP (listed to the left). In general, when temporary storage is applied to SSPs with continued warming followed by stabilized temperature (SSPs 4-3.4, 2-4.5, 4-6.0, 3-7.0, and 5-8.5), there is almost no difference in the multi-century regional temperature response between storage and reference SSPs. However, SSPs with net-negative emissions and peak-and-decline temperature pathways (SSPs 1-1.9, 1-2.6, and 5-3.4) generally exhibit more reginal temperature differences with reference SSPs, particularly in the Northern high latitudes and in the North Atlantic.*

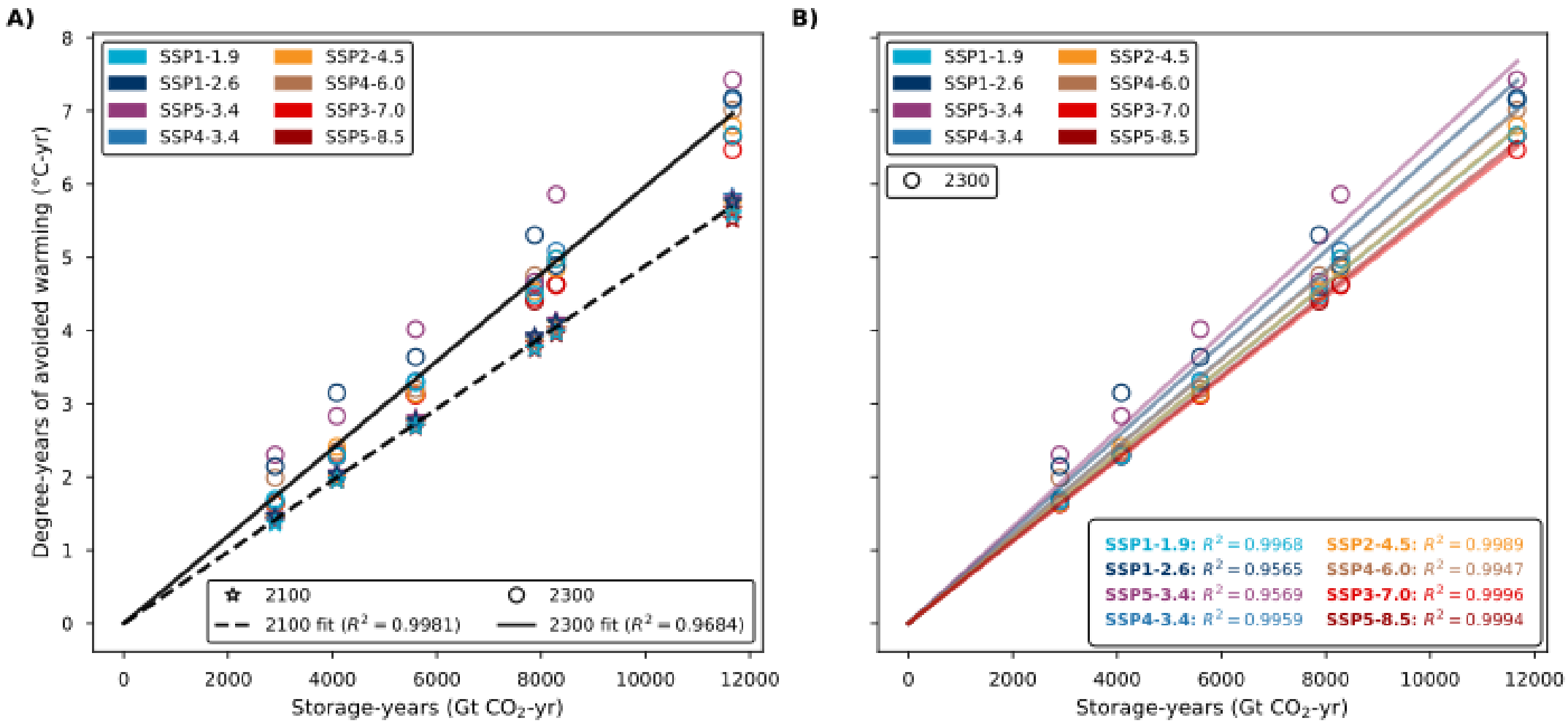

Supplementary Figure 3 – Linear relationships between storage-years of and degree-years storage SSPs*. This figure shows the relationship between storage-years of temporary carbon storage and degree-years of avoided warming across the six time-limited carbon storage pathways applied to each reference SSP. Panel A) shows the linear relationships across all storage SSPs at 2100 (dashed line) and 2300 (solid line). Panel B) shows the linear relationships within each SSP ensemble in 2300 with the colours of the lines representing the SSP ensemble.*

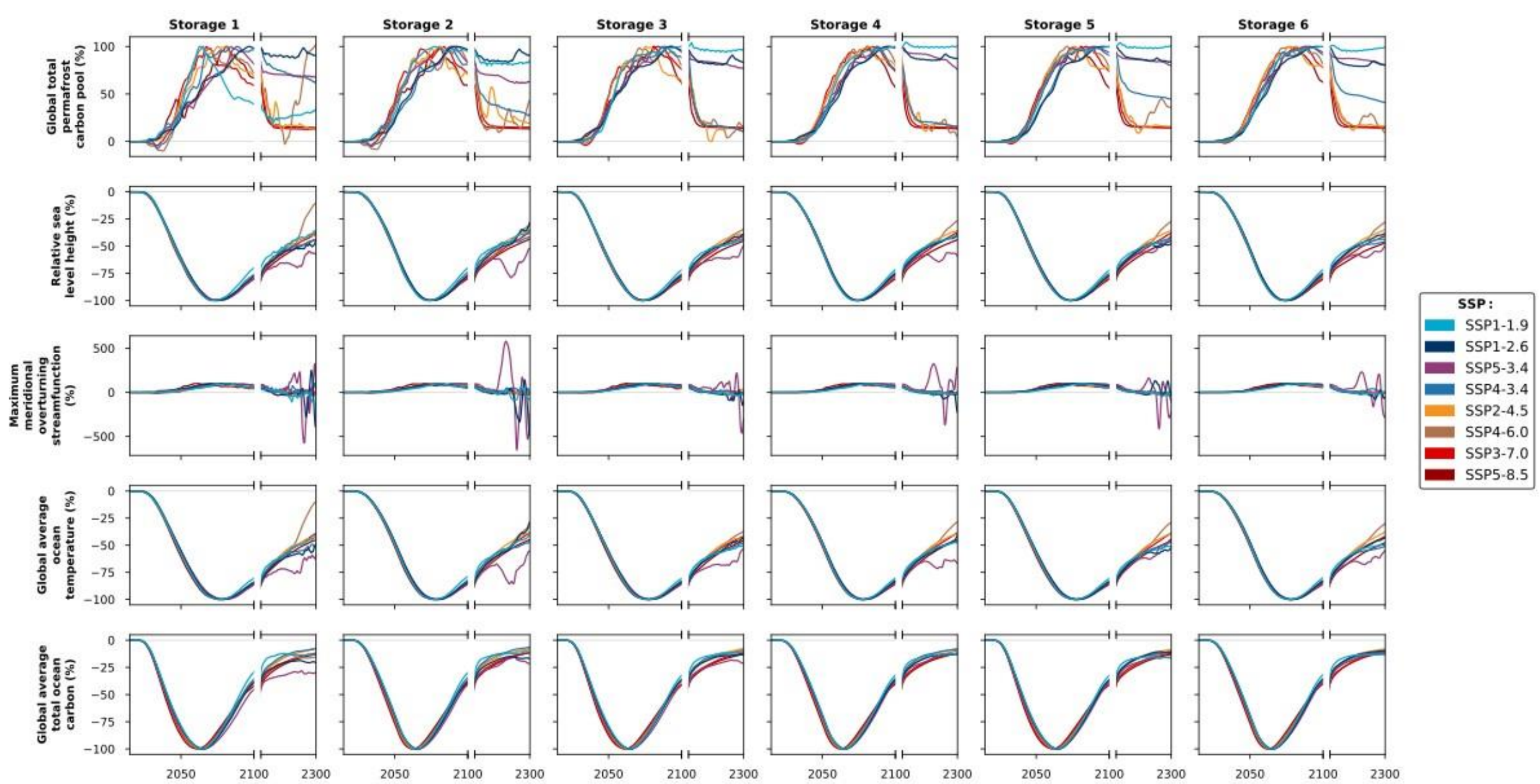

Supplementary Figure 4 – Normalized effect of temporary storage between storage and reference SSPs to 2300*. Each subplot shows the effect of temporary carbon storage relative to reference SSPs for a single slow-responding variable under one storage pathway. The effect of temporary carbon storage in each subplot is normalized to the maximum effect of storage occurring in the 21st century. Rows correspond to climate variables (listed on the left), and columns correspond to the six time-limited carbon storage pathways applied to each reference SSP (listed above). Coloured lines in each subplot indicate the storage SSPs. Values decreasing toward 0% indicate the erosion of the maximum benefit of temporary carbon storage seen in the 21st century. Permafrost carbon (top row) shows the highest retention of normalized benefit by 2300 in peak-and-decline scenarios, while AMOC (middle row) displays high scenario-dependent variability.*
