# Supplementary material for: Temporary carbon storage can mitigate slow-responding climate changes
Source: Nat Commun. 2026 Oct 16;17:11671. doi: 10.1038/s41467-026-78620-8 (PMC13759493; doi:10.1038/s41467-026-78620-8)
Supplement: Supplementary file 2 [file 41467_2026_78620_MOESM2_ESM.pdf]

nature portfolio

Peer Review File

Temporary carbon storage can mitigate slow-responding climate changes

Corresponding Author: Dr Mitchell Dickau

This manuscript has been previously reviewed at another journal that is not operating a transparent peer review scheme. The manuscript was considered suitable for publication without further review at Nature Communications.

This file contains all reviewer reports in order by version, followed by all author rebuttals in order by version.

Version 0:

Reviewer comments:

Reviewer #1

(Remarks to the Author)
Review of
Temporary land carbon storage can avoid slow-responding climate changes
By
Mitchell Dickau and H. Damon Matthews

Summary of noteworthy value:

This modelling study investigates the impact of temporary carbon storage on slow Earth system dynamics like meridional overturning ocean circulation, permafrost, ocean heat and carbon content. This is an important avenue of research relevant to a broad audience across Earth sciences and any discipline of relevance for understanding and informing global climate change and action. The key innovation is the idealized scenario-building that implements carbon removal entirely transiently, i.e. cumulative emissions in the carbon storage scenarios converge to the base scenarios within the 21st century. Additionally, studying such a large set of scenarios with an intermediate complexity model is helpful as it provides computational efficiency while more physical tractability then simple climate models. The key finding of long-term Earth system changes mitigated by temporary carbon storage is well-founded in analysis. However, the paper tends to be overly confident (e.g. “can avoid” rather than “can mitigate” in the title, see general and line-by-line comments for more) and lacks the necessary discussion of weaknesses in methodology and conclusion. In the current version, these prohibit publication, but given the valuable study question and design are worth to be revised and then reconsidered. The methodology is sound and meets expected standards. However, in a revised version, the authors either need to provide significantly more information for their general claims or need to retract their wording to more precise and particular claims.

General comments:

Centre the paper’s attention on the scientific novelty and strength of the modelling setup

In my opinion, the innovation of this study is not only to assume carbon storage to be temporary but to integrate to time horizons that are appropriate for these slow-moving subsystems. I understand the desire to carve out the significance for 21st century for communication to the broader public, but scientifically, the long-term dynamics are central. I therefor propose to centre the entire article around multi-century dynamics and to highlight impacts until 2100 as one aspect, inverting the current emphasis distribution.
Additional simulations and analysis or much more careful statement of findings
Decide, either provide additional simulations with different temporary storage length or no general statement about storage-year linearity. Actually so far, you only test this in the dimension of storage amount, not in the dimension of storage time (i.e. yielding equivalent sotrage-years with different storage amount and durations, i.e. different lengths of storage scenarios). I

understand that you already provide different storage scenarios with slightly different weight to early or late storage. But their variability does not suffice to show this across a significant range of length scales. As long as tested only along the magnitude dimension, I believe a general claim about storage-years proportionality is a unjustified, because too weak inductive conclusion. Please complement the existing simulations with setups that allow for the exploration of the storage time dimension if you want to convey the claim in this generality. Otherwise, please clarify that so far, you are only testing storage-AMOUNT to effect proportionality.
Furthermore, to connect to existing debate and assumptions, please add simulations with permanent or long-term storage as additional reference scenario. In some sense this is the limit scenario for longer and longer temporary storage. This will allow you to compute highly relevant metrics: the benefit overestimation under the faulty assumption of permanence.

Necessary discussion of limitations

The only dimension of limitation discussed at satisfying depth is scenario consistency.
However, the following lack completely or are non-exhaustive.

- Model suitability, discuss the robustness and reliability of each finding against the model choice and its weaknesses. There is a range of simulations with carbon storage assumptions in the literature. A comparison with results from these, e.g. with more sophisticated models, is completely lacking.

- A discussion of the general limitation of the storage-years proportionality framework. Why does it work here for many variables? How dependent is this on the model? What about scenarios with different storage length?

- What about internal variability (IntV)? Please discuss how much it is a limitation of this work to omit it. Infering from modeling studies covering IntV, what do you expect for these scenarios? Where could changes in variability lead to time asymmetries, more scenario dependence, and altered conclusions, where is it certainly negligible?

Line-by-line comments:

Title: “avoid” is an overstatement, use “mitigate” to avoid unscientific exaggeration.
Line 1: Why “nature-based” carbon storage? Land-based maybe? Or just not specify at all? In the modelling, it is idealized and non-specific.
Line 52-55: Include introductory statement about the limitations of this. E.g. on which models / analysis thus is rely?
Fig. 1: For me, a difference plot like D, but for emission rates, not only cumulative emissions would be very helpful to clearly see the timing of the 6 storage scenarios.
Line 91/92: this statement in my opinion is important enough to be included in the abstract. Actually, below in the discussion, you state it clearer “Our results also demonstrate that the climate benefits of temporary carbon storage 349 increase when paired with ambitious emissions reductions but diminish substantially in scenarios 350 where emissions continue to rise.” Try to include this statement in high-level sections like abstract and introduction.
Line 110: You only show and mostly discuss global mean effects of Temp carb stor. How about spatially explicit effects? Are there regions where there is disproportionally high benefit of temp carb stor and regions where there is less benefit? Corresponding discussion: How much spatial information can be trusted from UVic simulations?
Line 129: can you say more than this about spatial patterns of cooling, especially during storage? These patterns are crucial to explain the avoided impacts on slow responding Earth system elements and evaluate their credibility. For example, the cooling patterns and other relevant intermediate fields (like P-E in the north atlantic, ...) could be compared to patterns found in more detailed process models.
Fig . 3: For convenience and accesibility of Earth science readership, please convert m^3/s to Sverdrup. I propose to directly, potentially with a non-linear time axis, show time until 2300.
Line 177: actually, you only test this in the dimension of storage amount, not in the dimension of storage time (i.e. yielding equivalent sotrage-years with different storage amount and durations, i.e. different lengths of storage scenarios). As long as tested only along this dimension, I believe a general claim about storage-years proportionality is a unjustified, because too weak inductive conclusion. Please complement the existing simulations with setups that allow for the exploration of the storage time dimension if you want to convey the claim in this generality. Otherwise, please clarify that so far, you are only testing storage-AMOUNT to effect proportionality.
Fig. 5: Consider a vertical log- or symlog or manual non-linear scale to show borth large and small changes clearly.
Line 245: please contextualize this with the ZEC of your model and how you would expect this to play out in other models. Maybe briefly here already, but importantly in the discussion.
Fig. 6: isnt this the same as SM Fig. 5? And given this figure, can't you omit Fig. 4, whose info is contained in here?
Line 264: Fix the wrong caption text “Figure 4)”.
Line 290-292: Could this be because AMOC transitions through an edge state and initial conditions determine more than small variations in forcing, how it evolves? In this light, how reliable are these findings against internal variability?
Line 304-306: omit, that is stated above already.
Line 337-339: this is to be shown with different scenarios of different storage times. Otherwise this is only shown across storage magnitude.
Line 399-401: please make explicit where the model'slimitations lie for this study, which conclusions you expect to be equally drawn from findings reproduced by other models.
Fig. SM 4: In particular, I think SM Fig. shows important features of benefit decay that deserve the attention of the main

manuscript readers to allow for their transparent discussion. Moreover, a figure illustrating the relative longterm share of beneficial effect would help the understanding. E.g. renormalize Fig. SM to the peak effect (typically around 2060), so that e.g. global average total ocean carbon under Storage 6 would decay to 5% toward 2300, ocean temperature to 40-50%.

Reviewer #2

(Remarks to the Author)
I do not believe that the results presented in this paper are novel or noteworthy enough to be published in this journal. Many papers have been published in recent decades discussing the potential impacts of temporary carbon storage. The authors do not clearly explain how their work contributes to existing knowledge. Furthermore, one of the main limitations identified in the literature is that including altered albedo effects could significantly alter the conclusions regarding land carbon storage. The authors chose not to include these effects in their study. Including these effects would have produced more novel and, more importantly, more accurate conclusions. See below for more specific comments.
Figure 1: Adding a horizontal line at y=0 on graphs would help as we could better see when results become negative. Figure 1D is very difficult to read because there are too many curves and several of them overlap. I cannot really tell which set of curves represents storage 1 vs. storage 2, etc. In other words, I do not see if storage 1 is better than storage 2, etc.
Methods – Scenario design: It would be helpful to have a figure showing these six temporary carbon storage pathways. It would also be useful to justify the selection of the two temporal profiles. While maximum rates of sequestration were justified in the introduction, the two distinct pathways described in the Methods section were not.
Methods – Simulation design: “By modulating prescribed land-use CO_2 emissions rather than explicitly including the land-use and land-cover changes that would accompany many forms of nature-based carbon removal, our results reflect the biogeochemical effect of temporary carbon storage but not biogeophysical effects such as altered surface albedo or cloud cover.” This is a significant limitation that invalidates the study’s conclusions. As the literature shows, altered surface albedo can cause global temperature effects that are of the same order of magnitude as stored carbon, but opposite in sign. Therefore, the title of this paper, “Temporary land carbon storage can avoid slow-responding climate changes”, may not be true if this temporary land carbon storage occurs through afforestation projects in snow-covered regions, for example.
Results - Lines 114-147:
As stated by the authors (lines 132-136), the quasi-linearity between storage-years of temporary carbon storage and degree-years of avoided warming has already been demonstrated. These findings are not novel.
“This proportionality indicates that the duration and magnitude of carbon storage directly determine the duration and magnitude of avoided warming.” This is true when storage-years and degree-years are calculated for a time horizon that corresponds to the storage/release period (up until 2100 in this case). But what happens if we go beyond this time horizon? Several papers have been published in the last 25 years regarding the impact of temporary carbon storage. I feel that this section simply repeats what has already been shown without referencing this literature. Furthermore, it fails to show what will happen on a longer timescale after 2100, as temporary carbon storage may simply displace future warming effects.
Results – Lines 152-167:
“Unlike the effects of temporary carbon storage on temperature, slow-responding climate variables show persistent avoided changes that are not reversed once temporary carbon storage is re-emitted by 2100 (Fig. 3).” Since Figure 3 ends at 2100, it is unclear whether these changes are persistent. This conclusion is biased.
Results – Lines 213-226:
What do these results mean? How is this useful to know that?
Discussion – Lines 314-315: “For temperature, temporary storage can lower peak warming when combined with ambitious mitigation, which previous work shows would reduce the risk for triggering climate tipping points.” Since this study does not include altered albedo effects, we do not know. This claim may be incorrect.
Discussion – Lines 330-331: “In scenarios such as SSP1-1.9 and SSP1-2.6, which already rely on significant carbon dioxide removal to meet temperature targets, the addition of further storage may be unrealistic from both physical and socioeconomic perspectives.” This is very important. These conclusions may be useless due to this limitation.
Lines 348-349: “Our results also demonstrate that the climate benefits of temporary carbon storage increase when paired with ambitious emissions reductions but diminish substantially in scenarios where emissions continue to rise.” Since these temporary carbon storage pathways are probably not realistic under the most ambitious scenarios, as discussed in lines 330-331, and their benefits decrease under less ambitious reduction scenarios, as discussed in lines 348-349, the conclusion of the study is incorrect.

Reviewer #3

(Remarks to the Author)
This manuscript examines the climatic implications of temporary land carbon storage, arguing that the time-integrated amount of stored carbon (“carbon storage-years”) scales approximately linearly with avoided changes in several slow-responding climate variables, including ocean heat content, thermosteric sea-level rise, permafrost carbon loss, and AMOC strength. Using a set of idealized perturbation experiments within the UVic Earth System Climate Model, the authors explore how temporary carbon storage affects these slow components of the climate system across several emissions scenarios and storage magnitudes.

The topic is timely and potentially important. Temporary carbon storage is increasingly discussed in the context of land-based mitigation strategies, nature-based solutions, and carbon removal approaches. The manuscript provides an interesting perspective on how temporary carbon storage may influence slow components of the climate system through the

inertia of the carbon–climate response.

However, I have several concerns regarding novelty, framing, methodological scope, and interpretation of results, which should be addressed before the manuscript can be considered for publication.

1. Positioning within the broader literature on temporary carbon storage

The manuscript presents the storage-years framework largely within a relatively recent line of research. However, a broader literature has examined the climatic implications of temporary carbon sequestration and delayed emissions, including tonne-year approaches, storage-year metrics, and accounting methods for non-permanent sequestration.

Relevant earlier work includes IPCC (2000), Moura-Costa and Wilson, Lashof and Hare, Müller-Wenk and colleagues, Clift and colleagues, as well as industrial ecology and life-cycle assessment studies addressing temporary sequestration and delayed emissions (e.g., Brandao et al.). Standards such as PAS 2050 (BSI, 2008) also incorporate approaches to temporary carbon storage in product carbon footprinting.

Engaging more fully with this earlier literature would strengthen the manuscript by clarifying the conceptual lineage of the storage-years concept and by helping identify what is genuinely new in the present contribution. In particular, earlier work has examined the climatic implications of temporary sequestration through concepts such as delayed emissions, tonne-year equivalence, and dynamic carbon accounting. These studies already explored the relationship between temporary storage and time-integrated climate responses, which makes it important to clarify precisely what conceptual advance the present work provides beyond extending these relationships to additional variables and scenarios.

At present, the manuscript risks giving the impression that the core conceptual insight — linking integrated temporary storage to climate response — is largely novel, whereas related ideas have been explored in several contexts for more than two decades.

2. Framing as “temporary land carbon storage”

The manuscript is framed as an analysis of temporary land carbon storage, yet the experiments performed do not explicitly simulate land-use interventions or land-system dynamics. Instead, temporary storage is implemented by subtracting from and later adding to prescribed land-use CO_2 emissions, while land cover remains fixed and biogeophysical effects are excluded.

As a result, the experiments effectively represent idealized atmospheric CO_2 perturbations, rather than explicit land-based carbon storage systems. Real land-based carbon storage involves additional processes such as land-cover change, disturbance regimes, albedo effects, hydrological feedbacks, nutrient constraints, and non-CO_2 emissions.

The current experiments therefore demonstrate a property of the climate–carbon system response to temporary CO_2 perturbations, rather than the full climatic implications of temporary land carbon storage systems. This distinction should be made clearer in the manuscript, and the interpretation of the results should be adjusted accordingly.

3. Interpretation of the quasi-linear scaling

A central result of the manuscript is the quasi-linear relationship between storage-years and avoided changes in several slow-responding climate variables. This relationship is also consistent with the well-established near-proportionality between cumulative CO_2 emissions and global mean temperature change, which implies that time-integrated carbon perturbations will tend to scale with integrated climate responses. While this relationship is interesting, part of the observed proportionality may arise from the underlying relationship between cumulative CO_2 perturbations and global temperature change. Because storage-years effectively integrate the carbon perturbation over time, the scaling relationship may partly reflect the mathematical structure of the climate response to cumulative CO_2 emissions rather than a distinct emergent property specific to temporary carbon storage.

Clarifying this point would help readers better understand the conceptual contribution of the analysis.

4. Sensitivity to storage timing

The climatic implications of temporary carbon storage depend strongly on when carbon is removed and when it is re-emitted relative to the trajectory of atmospheric CO_2 concentrations and peak warming. In the present experiments, storage pathways appear to follow relatively structured uptake and re-emission patterns. It would be useful to clarify how sensitive the reported scaling relationships are to the assumed timing of storage and release, particularly relative to peak warming and net-zero transitions. Exploring alternative timing assumptions could help determine whether the observed scaling remains robust under different temporal structures of temporary storage.

5. Robustness of the scaling across climate variables

While the scaling relationship appears relatively consistent for some variables (e.g., thermosteric sea-level rise and ocean temperature), it appears less robust for others.

In particular:
- Permafrost carbon responses show noticeable scenario dependence.
- AMOC responses appear highly variable, especially over longer time horizons.

These results suggest that the proportionality between storage-years and climate responses may not be universally robust across all slow components of the climate system. The manuscript would benefit from a more nuanced discussion of where the scaling appears robust and where it becomes less reliable.

6. Dependence on a single Earth system model

All simulations are performed using UVic-ESCM, an intermediate-complexity Earth system model. While such models are valuable for conceptual experiments, conclusions regarding general properties of the climate system would ideally be supported by additional model evidence or uncertainty analysis.

At minimum, the manuscript should acknowledge more explicitly that the results represent model-based evidence from a single modelling framework, rather than a robust emergent property demonstrated across multiple climate models.

7. Interpretation for climate mitigation policy

The manuscript draws implications for the climate value of temporary carbon storage in mitigation portfolios and nature-based solutions. However, given the simplified experimental design and the absence of explicit land-system processes, the policy implications appear somewhat stronger than warranted by the analysis.

A more cautious discussion of the implications for real-world land-based mitigation strategies would improve the manuscript.

8. Minor comments
- There are several minor typographical issues in the manuscript (e.g., “DISSCUSSION”).
- Some figure captions would benefit from clearer descriptions of the storage pathways and scenario assumptions.
- The statistical characterization of the quasi-linear relationships could be strengthened by reporting regression slopes and uncertainty ranges in addition to R2 values. In addition, it would be helpful to indicate the range of storage magnitudes and scenarios over which the linear approximation holds. Providing confidence intervals for the fitted relationships or residual diagnostics would allow readers to better assess the robustness of the proposed scaling.

Version 1:

Reviewer comments:

Reviewer #1

(Remarks to the Author)
Review of
Temporary carbon storage can mitigate slow-responding climate changes
Mitchell Dickau and H. Damon Matthews

General comments:
The authors have largely taken my comments into account and resolved most critical aspects. The manuscript has improved in terms of breadth of audience and rigor. However, the manuscript does not seem publication-ready to me. Firstly, I strongly suggest to tone down the claims on the generality of the storage-years – avoided changes linearity even further. The authors have not provided additional evidence for its validity across a relevantly broad range of time scales. Additionally, the increased attention to the evolution until 2300 (that I appreciate) reveals its limits on longer time scales irrespective of different storage durations. Secondly, the value of the new modelling protocol concerning the use of SSP3-7.0 land use CO2 emissions as a baseline for all scenarios seems to be overstated with this giving a “neutral” baseline. Thirdly, figure design should be improved to optimize accessibility and interpretability. Finally, I suggest to improve the so far jargon-heavy language given the broad readership of the journal.
Line-by-line comments:

Line 19.21: The wording with doubled “that” is bulky, please improve the flow of this sentence.
Line 21-24: Qualify/detail this with the main limit to this linearity you find.
Line 87: “baseline simulation for each of the SSPs” can be confusing for scholars in the pathway modeling community as they use "baseline" for the SSP scenarios without RCP constraints. Moreover, the jargonized term "SSPs" typically is used to refer to the class of all scenarios sharing the same SSP, irrespective of RCP. Try rephrasing to "and eight reference simulations following the SSP-RCP scenarios established in CMIP6 using SSP1-1.9, ..., and SSP5-8.5 to generate...”
Line 90: “baseline SSPs” as detailed above, jargon that is different from the community-specific language and therefor confusing for large parts of the pathway modeling community. Adapt in the entire manuscript please.
Line 90, Methods reference: given your frequent relevance justification with land-based CDR and such being defined in the SSP-RCP forcings, you need to clarify your handling of land use already here to not confuse the readers concerning potential double accounting you actually avoid.
Line 92 “in both 2100 and 2300”: you actually present their evolution over time. You do more than just looking at these two

moments in time.
Line 94: consider starting a new sentence after "(AMOC)".
Line 105: If understand your interpretation right, you do not expect this to appear similarly in other models. If this is true, why not declare it with a wording like "...2100, model-specific ocean dynamics remove this proportionality by 2300".
Line 110-113: Are you able to include a number of typical relative overestimation like in lines 238-240?
Line 147: "see temporary storage" complicated language (if even correct, I am not a native speaker). Please simplify.
Line 149 "net-negative CO2": add "emissions"? This is a typical instance where the wording is overly shortened and jargonized. Please also work on other, similar instances.
Line 156-158: I appreciate the note on regional temperature patterns. In my original comment on spatially explicit results, I was referring to impacts like permafrost carbon and ocean carbon. I understand that a detailed study of impact patterns is beyond the scope of this study that focuses on richness in scenario space. However, one or two sentences on spatial features that explain some time series behaviour would help (thinking of permafrost carbon stock variations in SSP2-4.5 and SP1-2.6). I also appreciate a negative statement about spatial features or, better to say, a statement about the homogeneity of the responses in UVic-ESCM.
Line 204/205: this sounds as if it still exists, just with scenario-dependent slopes. But Fig. 2C sems to suggest the relationship to break down in some scenarios (although the vizualization makes it hard to tell). Please clarify and/or correct me should I have misunderstood the figure.
Line 210 "persistent": persistance sounds more stable than most elements of the climate system you assess seem to be. I think "non-zero" would be a careful wording but maybe "continued but declining" would improve accuracy overall.
Line 259-261: I believe already here, it needs to be made clear that your analysis only allows to infer this for varying magnitudes, not for varying durations.
Line 265 "further from when": improve the wording. at least "further from the moment in time, when"?
Line 445-449: make sure to discuss in comparison to the closely related

Steinert, N.J., Schwinger, J., Burke, E. et al. Permafrost carbon release scales linearly with overshoot warming mediated by AMOC tipping. Nat Commun 17, 6395 (2026). https://doi.org/10.1038/s41467-026-73612-0

Line 452-457: how do you see your results depending on the degree of TCRE reversibility (cooling per removal as a separate phenomenon to ZEC)?

Line 462-464: here, again consider Steinert et al., 2026, https://doi.org/10.1038/s41467-026-73612-0

Line 494 " avoided change": clarify BGC only again in this sentence. Your measure does not account for the net avoided change.

Line 542-543: this makes a lot of sense, but choosing SSP3-7.0 land use CO2 emissions and not zero emissions is a surprising choice. You argue that "adding large further 545 storage perturbations to these SSPs would likely produce carbon removal pathways that are quite unrealistic from a technical, social, and/or economic sense" but how should the combination of continued deforestation in most of Africa be more consistent or realistic in combination with your removal pathways (especially the high-removal volume ones)? Why not choose a no Land use change reference case that allows for a cas much closer to what you claim to be "neutral"? Like this, you increased comparability among scenarios but did not increase realism in my opinion. Consider revising the modeling protocol or tone down your claims about "neutrality".

(Remarks on code availability)

Reviewer #2

(Remarks to the Author)
I thank the authors for their thorough revisions in response to the reviewers' comments. They have fully addressed all of my concerns. I recommend the manuscript for publication in its current form.

(Remarks on code availability)

Reviewer #3

(Remarks to the Author)
I thank the authors for their careful and thorough response to the reviewers' comments. The manuscript has been substantially improved. As a minor suggestion, the authors may also wish to cite one or two more recent comparative studies on climate metrics for temporary carbon storage and land carbon fluxes to complement the historical overview, e.g. Brandao et al., 2019, 2024.

(Remarks on code availability)

Version 2:

Reviewer comments:

Reviewer #1

(Remarks to the Author)
The authors have successfully responded to my remaining concerns from last round and clarified text and figures. The paper seems ready for publication.

(Remarks on code availability)

Open Access This Peer Review File is licensed under a Creative Commons Attribution 4.0 International License, which permits use, sharing, adaptation, distribution and reproduction in any medium or format, as long as you give appropriate credit to the original author(s) and the source, provide a link to the Creative Commons license, and indicate if changes were made.
In cases where reviewers are anonymous, credit should be given to 'Anonymous Referee' and the source.
The images or other third party material in this Peer Review File are included in the article's Creative Commons license, unless indicated otherwise in a credit line to the material. If material is not included in the article's Creative Commons license and your intended use is not permitted by statutory regulation or exceeds the permitted use, you will need to obtain permission directly from the copyright holder.
To view a copy of this license, visit https://creativecommons.org/licenses/by/4.0/

Review of "*Temporary land carbon storage can avoid slow-responding climate changes*"

By Mitchell Dickau and H. Damon Matthews

Reviewer comments in bold. Author responses italicized and un-bolded.

Reviewer #1 (Remarks to the Author):

Summary of noteworthy value:

This modelling study investigates the impact of temporary carbon storage on slow Earth system dynamics like meridional overturning ocean circulation, permafrost, ocean heat and carbon content. This is an important avenue of research relevant to a broad audience across Earth sciences and any discipline of relevance for understanding and informing global climate change and action. The key innovation is the idealized scenario-building that implements carbon removal entirely transiently, i.e. cumulative emissions in the carbon storage scenarios converge to the base scenarios within the 21st century. Additionally, studying such a large set of scenarios with an intermediate complexity model is helpful as it provides computational efficiency while more physical tractability then simple climate models. The key finding of long-term Earth system changes mitigated by temporary carbon storage is well-founded in analysis. However, the paper tends to be overly confident (e.g. "can avoid" rather than "can mitigate" in the title, see general and line-by-line comments for more) and lacks the necessary discussion of weaknesses in methodology and conclusion. In the current version, these prohibit publication, but given the valuable study question and design are worth to be revised and then reconsidered. The methodology is sound and meets expected standards. However, in a revised version, the authors either need to provide significantly more information for their general claims or need to retract their wording to more precise and particular claims.

We thank Reviewer 1 for their assessment and for acknowledging the research's importance to a broad audience. We appreciate their recognition of the value in using a model with more physical tractability than simple climate models and for investigating the climate impacts of temporary carbon storage in a large scenario ensemble. We have addressed concerns regarding terminology and transparency through the revisions detailed below. We believe these adjustments resolve the criticisms and improve the work's rigor. Point-by-point responses are provided below.

General comments:

Centre the paper's attention on the scientific novelty and strength of the modelling setup

In my opinion, the innovation of this study is not only to assume carbon storage to be temporary but to integrate to time horizons that are appropriate for these slow-moving subsystems. I understand the desire to carve out the significance for 21st century for communication to the broader public, but scientifically, the long-term dynamics are central. I therefor propose to centre the entire article around multi-century dynamics and to highlight impacts until 2100 as one aspect, inverting the current emphasis distribution.

We thank the reviewer for highlighting the significance of multi-century timelines when evaluating slow-responding climate subsystems. We agree that a robust assessment requires a comprehensive view of long-term dynamics. However, we believe that the 2100 and 2300 time

horizons are equally important given the more direct policy relevance of this century in relation to the goals of the Paris Agreement. To reflect this balanced perspective, we have restructured the manuscript to discuss the 2100 and 2300 results in tandem throughout the narrative, and we have extended all figures to include the 2100 and 2300 results in the main manuscript rather than in the supplementary information. Consequently, we have removed the standalone section titled "MULTI-CENTURY RESPONSE TO TEMPORARY CARBON STORAGE" and integrated its text directly into the previous sections. This restructuring ensures that both timelines receive equal emphasis.

Additional simulations and analysis or much more careful statement of findings

Decide, either provide additional simulations with different temporary storage length or no general statement about storage-year linearity. Actually so far, you only test this in the dimension of storage amount, not in the dimension of storage time (i.e. yielding equivalent storage-years with different storage amount and durations, i.e. different lengths of storage scenarios). I understand that you already provide different storage scenarios with slightly different weight to early or late storage. But their variability does not suffice to show this across a significant range of length scales. As long as tested only along the magnitude dimension, I believe a general claim about storage-years proportionality is a unjustified, because too weak inductive conclusion. Please complement the existing simulations with setups that allow for the exploration of the storage time dimension if you want to convey the claim in this generality. Otherwise, please clarify that so far, you are only testing storage-AMOUNT to effect proportionality.

We acknowledge Reviewer 1's highly relevant point regarding the importance of the time dimension in the storage-years framework. While exploring varied storage durations would be a valuable contribution to the literature, the reviewer is correct that we are primarily testing the proportionality here between storage-year amounts and climate changes at particular points in time. In fact, we would expect proportionality to be less robust with widely varying storage duration, since all of the climate variables we highlight here do show some reversibility, and hence are more likely to diverge from a proportional relationship with storage-years if different scenarios had longer or short periods of time from the end of storage to the year 2100 or 2300. We have therefore moderated our claims to specify that the proportionality was tested only along the storage-amount dimension. We have added text to the Discussion clarifying that further research is required to confirm the robustness of the storage-years metric across diverse different timescales of storage and re-emission.

> *(Added text to the discussion): Furthermore, the storage rates applied here are highly idealized, which is an important consideration given that the efficacy of temporary storage appears to be partially contingent on the timing of sequestration and re-emission relative to peak warming. For instance, if temporary storage is re-emitted prior to net-zero CO2, as opposed to after net-zero CO2 as in this study for the peak-and-decline SSPs, storage may merely shift the peak's timing without reducing its magnitude. Therefore, while this work confirms that the proportionality between storage-years and avoided inertial climate changes holds across a wide range of storage magnitudes, a wider variety of temporal profiles of temporary carbon storage, in addition to the two*

temporal profiles simulated here, would have to be used to explore how well this proportionality holds across varying durations of temporary carbon storage.

Furthermore, to connect to existing debate and assumptions, please add simulations with permanent or long-term storage as additional reference scenario. In some sense this is the limit scenario for longer and longer temporary storage. This will allow you to compute highly relevant metrics: the benefit overestimation under the faulty assumption of permanence.

Following Reviewer 1's suggestion, we have added three permanent storage pathways that are applied to each SSP baseline (24 additional simulations). These simulations follow temporary storage pathways 2, 4, and 6 for the carbon storage phase, but this stored carbon remains sequestered until 2300. Using these simulations, we quantify the "benefit overestimation" that occurs when permanence is incorrectly assumed, as Reviewer 1 has suggested. See the following changes:

(Added text to CONTEXTUALIZING THE CLIMATE BENEFITS OF TEMPORARY CARBON STORAGE section in the RESULTS):

To estimate the difference in the climate benefit of non-permanent sequestration relative to permanent carbon removal, we compare three of our temporary storage pathways against three removal pathways where the removed carbon is stored permanently until 2300. These removal pathways follow storage pathways two, four, and six in the storage phase, but sequestered carbon remains sequestered until 2300 rather than being re-emitted in the second half of the 21st century (see Methods). By taking the difference of slow-responding variables between permanent removal and temporary storage pathways, we can quantify the potential benefit overestimation that occurs when carbon storage is incorrectly assumed to be permanent.

Unsurprisingly, permanent removal pathways result in more avoided change than the temporary storage pathways for all SSPs and all variables. For thermosteric sea-level rise, ocean temperature, and total ocean carbon (Fig. 8), permanent removal has an increasing benefit over time relative to non-permanent storage. The added benefit of permanent removal has a clear scenario dependence – the SSPs with the highest end-of-century warming (i.e., SSP5-8.5, SSP3-7.0) see the climate benefit of permanent removal accumulate more slowly. For permafrost carbon, permanent removal avoids more permafrost carbon loss relative to temporary storage. However, peak-and-decline SSPs see avoided permafrost carbon loss accumulate throughout the simulations, while high-emissions SSPs see the benefit of permanent removal decrease briefly as temperatures stabilize before continuing to accumulate (Fig. 8, A, B, & C). Finally, the AMOC response to permanent removal pathways sees brief periods (years to decades) of less climate benefit than temporary pathways due to high AMOC variability in SSP1-1.9, SSP1-2.6, SSP5-3.4, and SSP4-6.0 (Fig. 8, D, E, & F), but otherwise permanent removal pathways lead to less AMOC slowdown relative to temporary carbon storage pathways.

(New Figure 8 showing benefit overestimation):

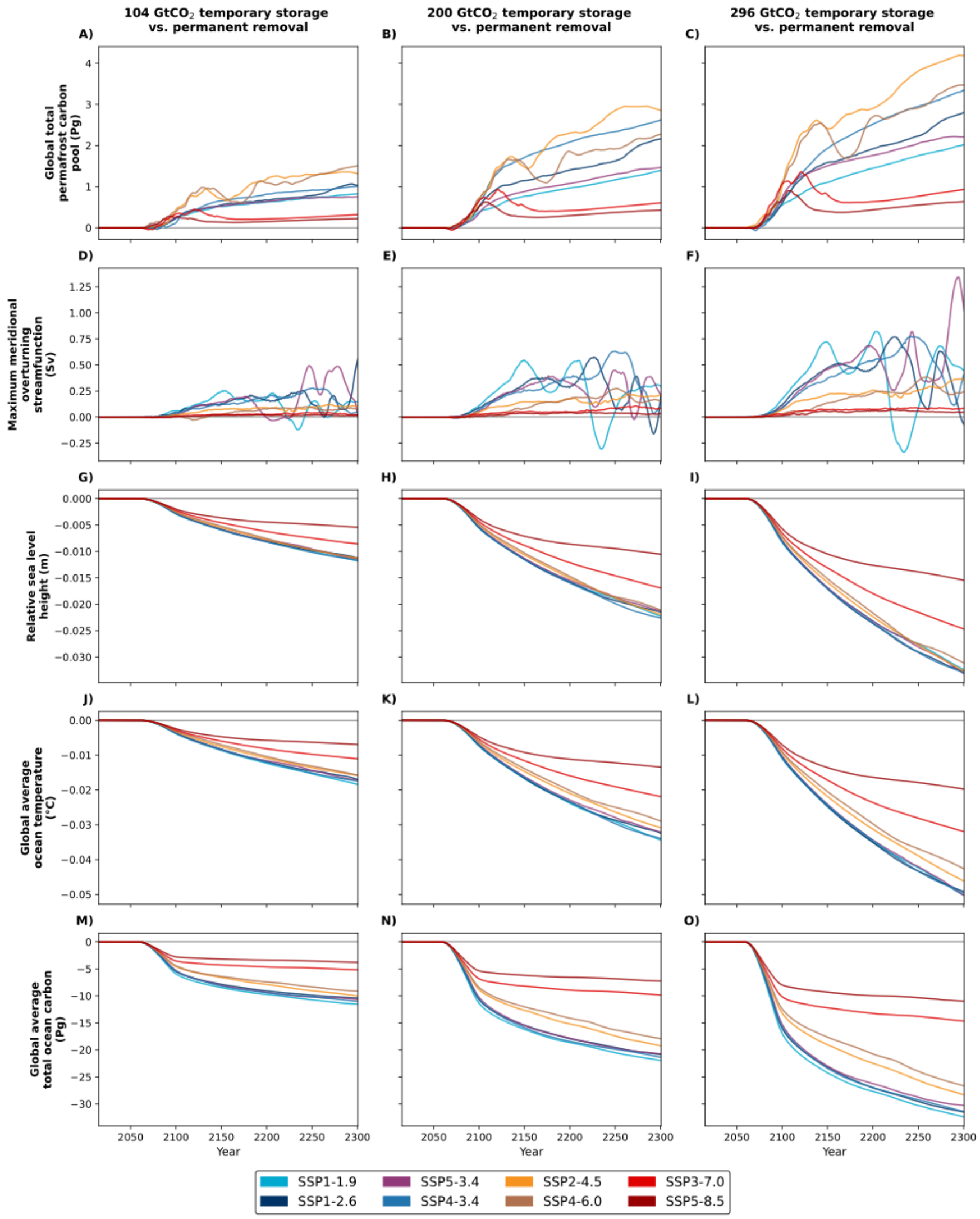

Necessary discussion of limitations

The only dimension of limitation discussed at satisfying depth is scenario consistency. However, the following lack completely or are non-exhaustive.

- Model suitability, discuss the robustness and reliability of each finding against the model choice and its weaknesses. There is a range of simulations with carbon storage assumptions in the literature. A comparison with results from these, e.g. with more sophisticated models, is completely lacking.

- A discussion of the general limitation of the storage-years proportionality framework. Why does it work here for many variables? How dependent is this on the model? What about scenarios with different storage length?

- What about internal variability (IntV)? Please discuss how much it is a limitation of this work to omit it. Inferring from modeling studies covering IntV, what do you expect for these scenarios? Where could changes in variability lead to time asymmetries, more scenario dependence, and altered conclusions, where is it certainly negligible?

We agree with Reviewer 1 that the discussion would benefit from a more detailed discussion of the potential limitations of this framework. To address this concern, we have added the following paragraph to the discussion:

> *(Added text to the discussion): The proportionality between storage-years and the impact of temporary carbon storage found here is subject to the physical characteristics of the variables examined. Here, this framework generally holds for slow-responding globally averaged stock variables because these components possess high physical inertia. Aside from the AMOC, which is the only flux variable in our analysis, these variables act as low-pass filters that integrate the cumulative radiative forcing anomaly over time, smoothing out variations in the precise timing of carbon sequestration and release. This proportionality may also be subject to the specific characteristics of the UVic-ESCM. A key structural characteristic of the UVic-ESCM is its single-layer atmosphere and prescribed atmospheric circulation, which prevents the model from generating significant interannual atmospheric variability. The response of a full-complexity model would be additionally affected by unforced decadal atmospheric variability which could introduce greater scenario dependence. Another consideration is that the proportionality found here is a function of the UVic-ESCM's TCRE given that the fundamental proportionality between carbon storage-years and degree-years of avoided warming arises directly from TCRE linearity; a model with a different TCRE value should simulate a different magnitude of proportionality during the period of active storage-years (i.e., up to 2100). Additionally, differences in ZEC values among models would change the relationship between storage-years and changes occurring after storage-years stop accumulating (i.e., from 2100 to 2300). We expect that our conclusion of proportionality at the year 2100 would hold across other models given the consistent linearity of the TCRE relationship among models, but that changes after 2100 would be more sensitive to differences in ZEC across models. Future multi-model intercomparisons would be an important next step to verify whether this storage-year proportionality remains robust across different climate model structures and complexities.*
>
> .

Line-by-line comments:

Title: "avoid" is an overstatement, use "mitigate" to avoid unscientific exaggeration.

We have replaced "avoid" with "mitigate" in the manuscript title.

Line 1: Why "nature-based" carbon storage? Land-based maybe? Or just not specify at all? In the modelling, it is idealized and non-specific.

We have added text clarify that our study design tests solely the biogeochemical effects of temporary carbon storage, making it applicable to a variety of CDR methods rather than specific to nature-based or land-based carbon removal and storage. However, we have decided to keep part of the discussion of nature-based CDR in the introduction because its prominence in climate mitigation portfolios and because many forms of nature-based CDR may only provide temporary carbon storage. In our view, these characteristics of nature-based CDR are one of the main reasons why understanding the climate outcomes of temporary carbon storage is crucial.

(Additions and revisions):

- *We have revised the title to be "Temporary land carbon storage…." To "Temporary carbon storage…"*
- *In the introduction, we have replaced seven mentions of "nature-based carbon storage" with simply "carbon storage", "temporary carbon storage"*

Line 52-55: Include introductory statement about the limitations of this. E.g. on which models / analysis thus is rely?

We agree with the reviewer that explicitly stating the underlying models/analysis of earlier studies improves clarity. In response to this suggestion, we have added the following text to the introduction:

> *(Added text): Existing evaluations have quantified these relationships using analytical accounting formulations, localized ecosystem carbon-cycle models, and an intermediate-complexity climate model.*

Fig. 1: For me, a difference plot like D, but for emission rates, not only cumulative emissions would be very helpful to clearly see the timing of the 6 storage scenarios.

We have revised Figure 1 to include difference plots for cumulative and annual CO_2 emissions in response to Reviewer 1's insightful suggestion, and we have extended these subfigures to 2300 in response to Reviewer 1's earlier suggestion to place more emphasis on the 2300 results.

> *(Revised Figure 1):*

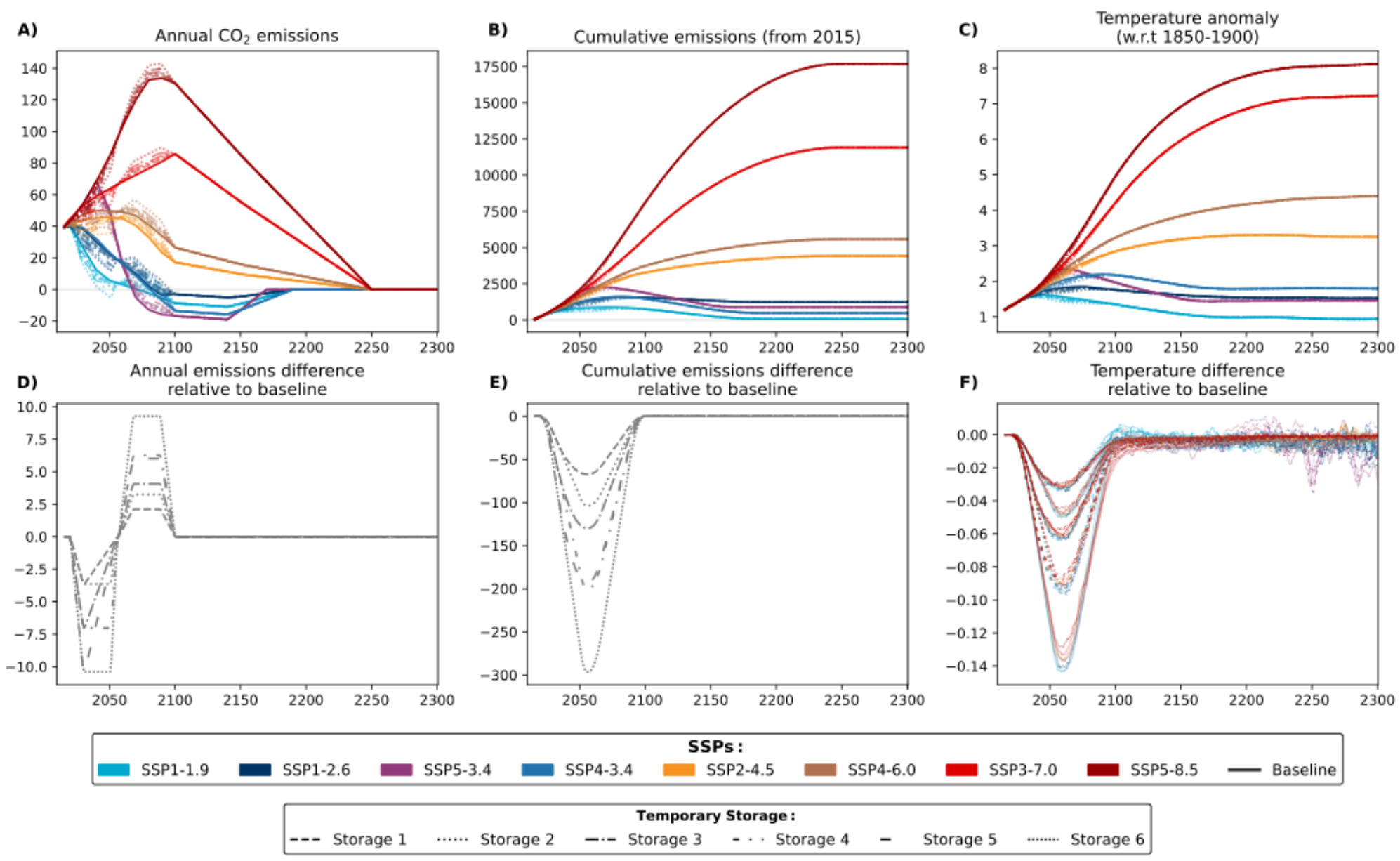

As a result of showing the results of the simulations until 2300 rather than 2100 as we did in the original manuscript, it is difficult to see differences in peak temperature between storage and baseline SSPs in Figure 1 A. To account for this, we have added a new figure to the manuscript showing differences in peak temperature in storage SSPs relative to their baselines.

(Added figure – new Figure 3):

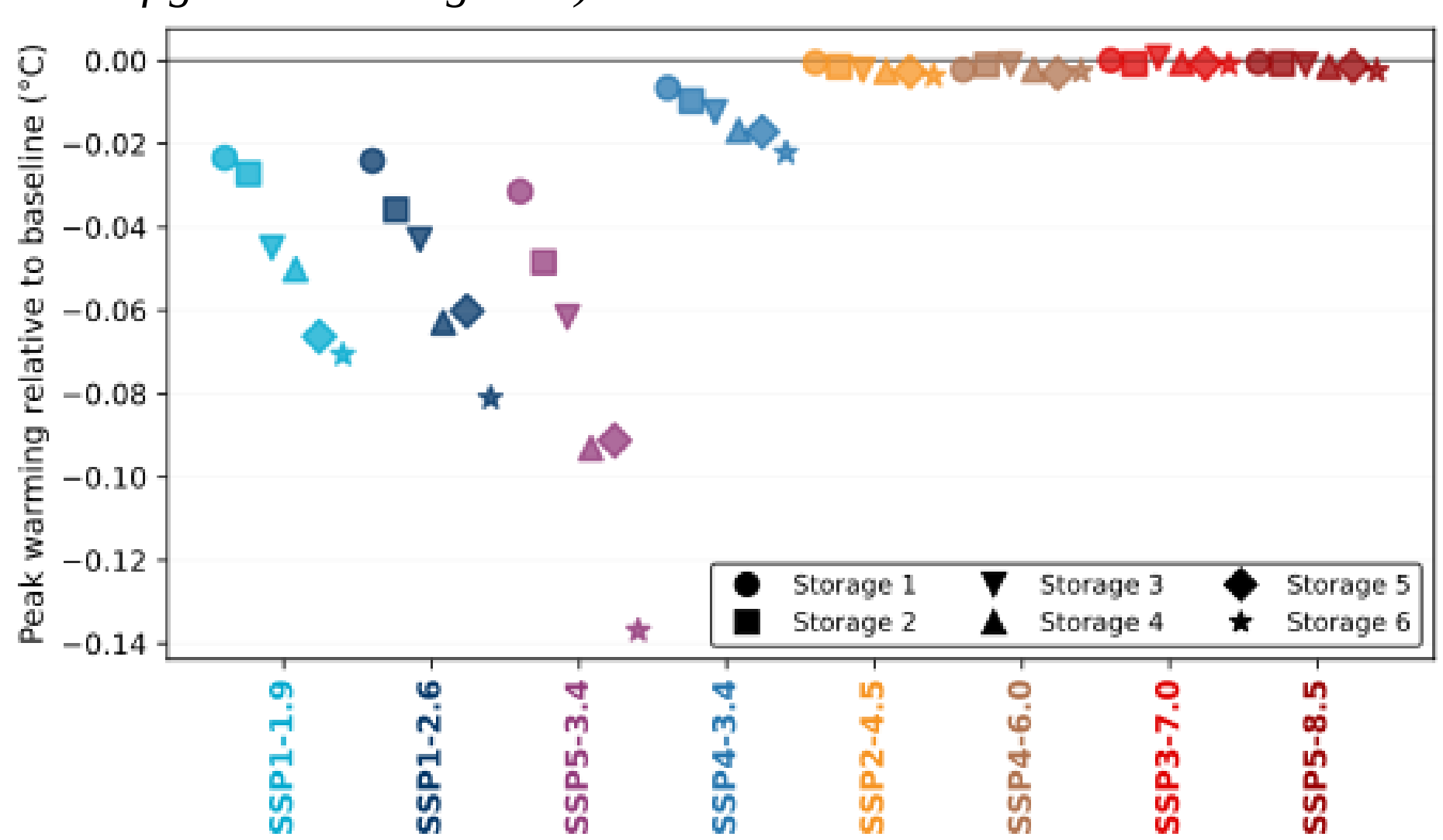

Line 91/92: this statement in my opinion is important enough to be included in the abstract. Actually, below in the discussion, you state it clearer "Our results also demonstrate that the climate benefits of temporary carbon storage 349 increase when paired with ambitious emissions reductions but diminish substantially in scenarios 350 where emissions continue to rise." Try to include this statement in high-level sections like abstract and introduction.

We thank Reviewer 1 for the recognition of the importance of this finding and have added this statement to the abstract:

> *(Added text to the abstract): These climate benefits are greatest when temporary carbon storage is paired with ambitious mitigation.*

We have also stated this finding in more detail in the introduction:

> *(Original): On multi-century timescales, retained benefits are smaller in high-emission stabilization scenarios, while peak-and-decline scenarios retain persistent legacy effects, particularly for avoided permafrost carbon loss.*

> *(Revised): On multi-century timescales, retained benefits are smaller in high-emissions scenarios, while peak-and-decline scenarios retain persistent legacy effects, particularly for avoided permafrost carbon loss, indicating that the climate benefit of temporary carbon storage is greater when paired with more ambitious mitigation.*

Line 110: You only show and mostly discuss global mean effects of Temp carb stor. How about spatially explicit effects? Are there regions where there is disproportionally high benefit of temp carb stor and regions where there is less benefit? Corresponding discussion: How much spatial information can be trusted from UVic simulations?

We thank the reviewer for raising this important point regarding the spatial distribution of temporary carbon storage impacts. To provide regional context, we have added a spatial analysis of multi-century surface air temperature (SAT) changes in the Supplementary Material (Supplementary Figure 2) to accompany the 2100 spatial SAT analysis included in the original supplementary material (Supplementary Figure 1). In addition to the original discussion of the regional SAT response in 2100, we have added the following discussion of the regional variation in the benefit of temporary carbon storage in 2300 to the results:

> *(Added text to the Results): By 2300, most of the regional differences in temperature between storage and baseline SSPs is erased for high-emissions SSPs that do not reach net-negative CO2 (Supplementary Fig. 2). However, in peak-and-decline SSPs, temporary storage continues to cause regional temperature differences relative to baseline SSPs (Supplementary Fig. 2). These regional temperature differences of both cooling and warming in 2300 are concentrated in the northern high latitudes, particularly the North Atlantic, and are likely driven by increased AMOC variability seen in peak-and-decline SSPs (discussed below).*

(Added figure to Supplementary Information):

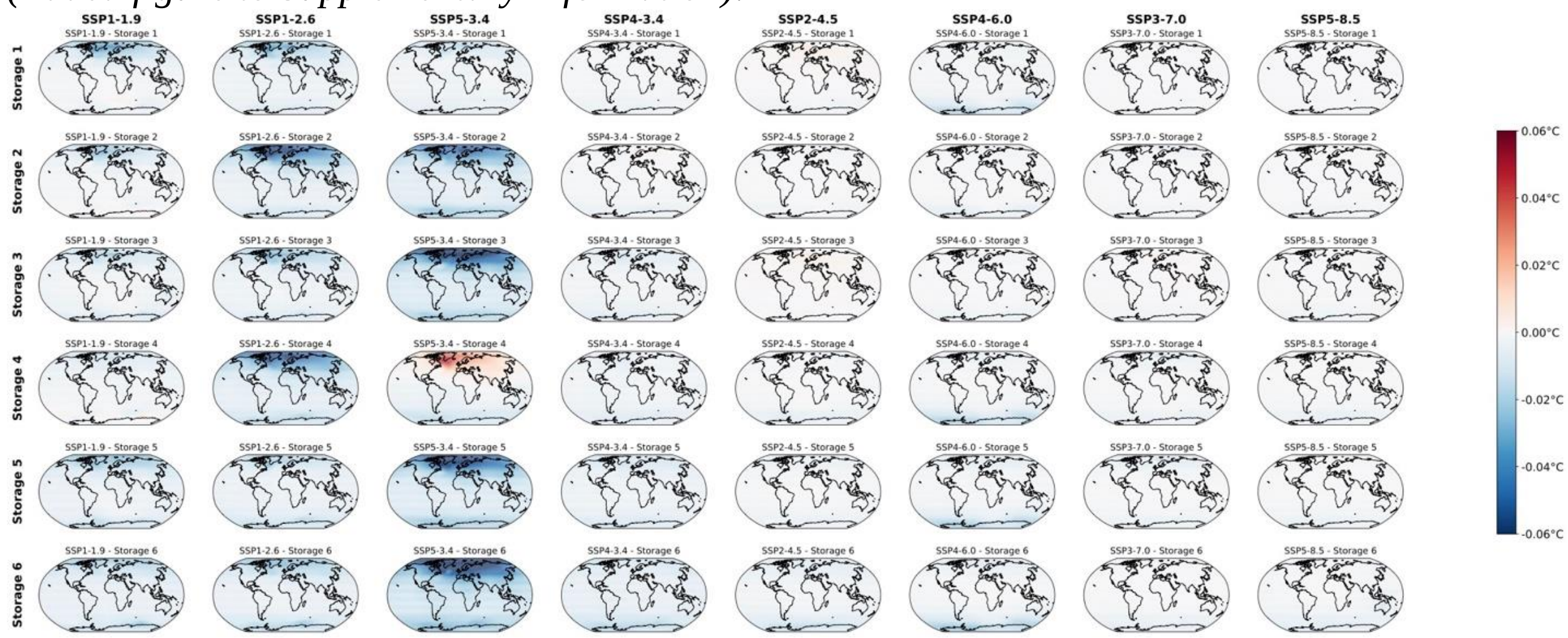

Regarding the trustworthiness of spatial patterns within our intermediate-complexity framework, the UVic-ESCM effectively simulates large-scale spatial temperature changes and deep ocean dynamics, but simulates localized processes governed by precipitation patterns or complex atmospheric circulation more simply due to its single-layer atmosphere and prescribed winds. Because the primary objective of this paper is to establish the global-scale physical properties and quasi-linear scaling boundaries of the storage-years metric, we have intentionally focused our primary analysis on global macro-indicators rather than a descriptive regional analysis of every individual subsystem.

Line 129: can you say more than this about spatial patterns of cooling, especially during storage? These patterns are crucial to explain the avoided impacts on slow responding Earth system elements and evaluate their credibility. For example, the cooling patterns and other relevant intermediate fields (like P-E in the north atlantic, ...) could be compared to patterns found in more detailed process models.

We thank the reviewer for the suggestion to provide a more detailed spatial evaluation of cooling patterns and intermediate environmental fields during the carbon storage phase. While we agree that evaluating highly specific regional indicators or comparing these local dynamics against detailed models, we believe a comprehensive spatial analysis is outside the primary scope of this paper. The core objective of our study is to investigate the global-scale physical properties established by the storage-years framework. Introducing extensive regional climate dynamics and intermediate atmospheric diagnostics would add significant complexity that risks distracting from these central global indicators. However, to provide additional spatial context without obscuring our primary global focus, we have retained the 2100 surface air temperature anomaly maps and expanded the multi-century regional patterns up to the year 2300 within the Supplementary Information (Supplementary Figures 1 and 2).

Fig . 3: For convenience and accessibility of Earth science readership, please convert m^3/s to Sverdrup. I propose to directly, potentially with a non-linear time axis, show time until 2300.

We have revised all figures with the maximum meridional overturning circulation to be in Sv rather than m^3/s, and we have made Figure 3 run to 2300 with a broken axis following Reviewer 1's helpful suggestions.

(Revised Figure 3 (now figure 4)):

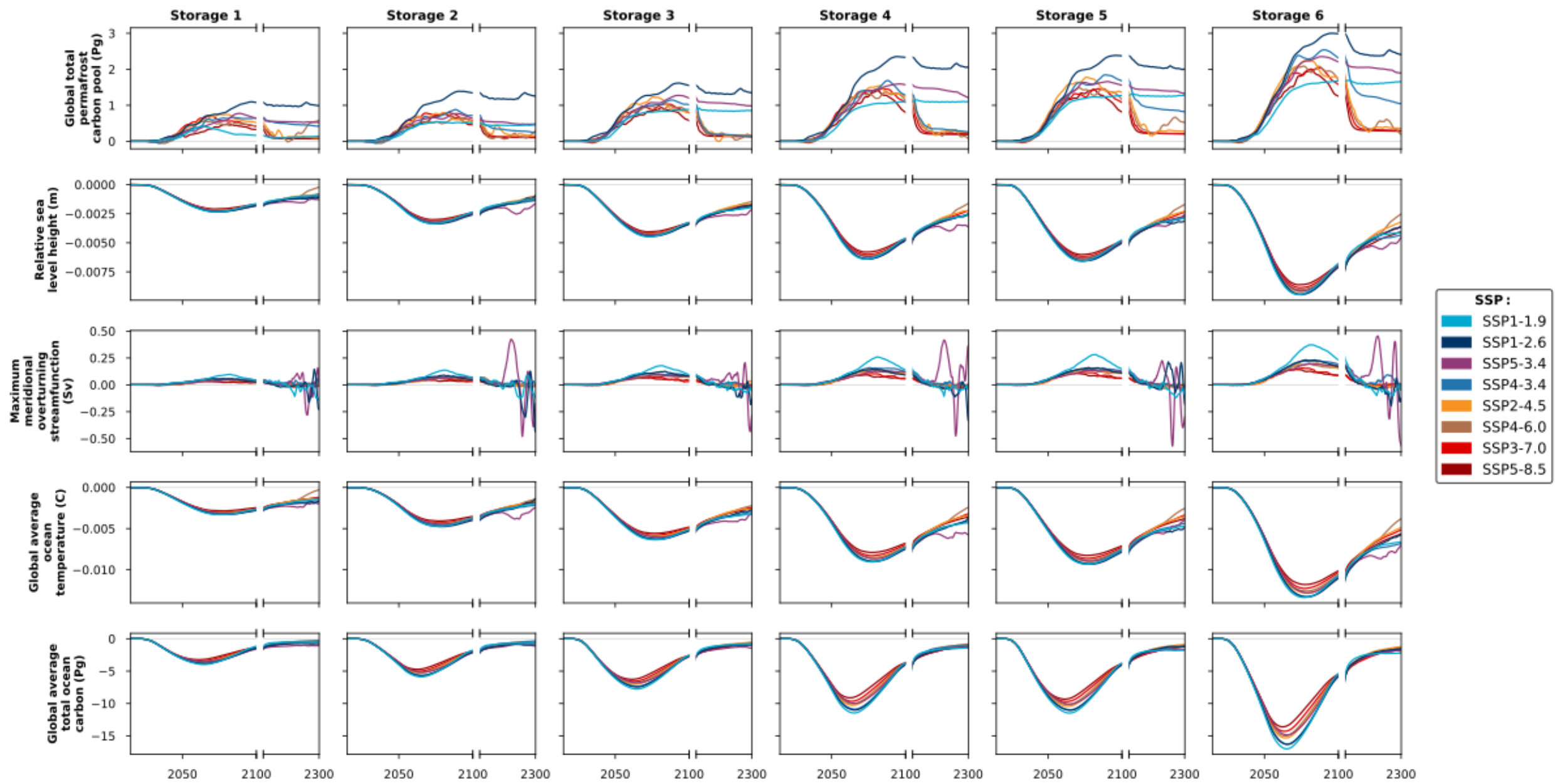

Line 177: actually, you only test this in the dimension of storage amount, not in the dimension of storage time (i.e. yielding equivalent sotrage-years with different storage amount and durations, i.e. different lengths of storage scenarios). As long as tested only along this dimension, I believe a general claim about storage-years proportionality is a unjustified, because too weak inductive conclusion. Please complement the existing simulations with setups that allow for the exploration of the storage time dimension if you want to convey the claim in this generality. Otherwise, please clarify that so far, you are only testing storage-AMOUNT to effect proportionality.

We agree with the reviewer that exploring the independent effect of storage duration is an important piece for fully validating the generality of the framework. As noted in our previous response, a comprehensive exploration of this storage time dimension represents an added level complexity that warrants a dedicated standalone investigation. To address your concern and avoid an overgeneralized inductive conclusion, we have added a clear statement to the Discussion outlining this boundary condition, highlighting the need for future research to explore how varied storage timescales and release profiles impact the robustness of the storage-years proportionality.

> *(Added text to the Discussion): Therefore, while this work confirms that the proportionality between storage-years and avoided inertial climate changes holds across a wide range of storage magnitudes, a wider variety of temporal profiles of temporary carbon storage, in addition to the two temporal profiles simulated here, would have to be*

used to explore how well this proportionality holds across varying durations of temporary carbon storage.

Fig. 5: Consider a vertical log- or symlog or manual non-linear scale to show borth large and small changes clearly.

We have revised Figure 5 to have a symlog scale as suggested by Reviewer 1.

(Revised Figure 5 – Figure 7 in the revised manuscript):

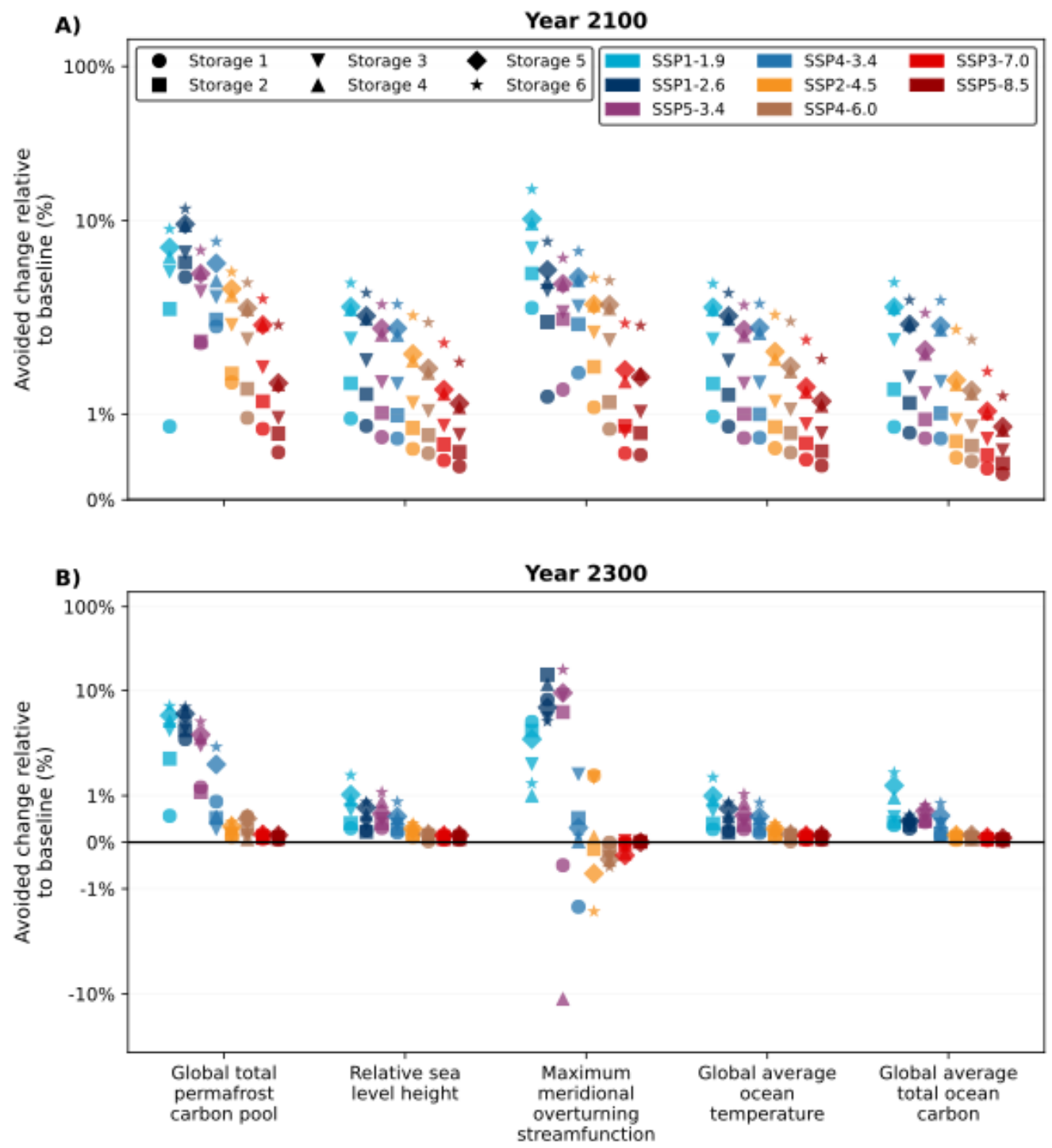

Line 245: please contextualize this with the ZEC of your model and how you would expect this to play out in other models. Maybe briefly here already, but importantly in the discussion.

We have added the following brief contextualization of this result:

(Added text to Results): This accumulation of degree-years is driven in part by the zero emissions commitment (ZEC). The simulated ZEC of our model is close to zero during the first century following the cessation of positive emissions but increases to cause slow warming over multi-century timescales [40]. For pathways with reduced or negative emissions relative to a baseline, we would expect that the model's positive multi-century ZEC value would cause sustained cooling and the accumulation of degree-years observed here in the storage SSPs. This multi-century accumulation of degree-years is likely caused by reduced permafrost carbon release in the storage SSPs, which is also the mechanism behind the model's positive multi-century ZEC [40]. Evidence for this

permafrost mechanism can be seen in the accumulation of degree-years of avoided warming being generally more pronounced in peak-and-decline SSPs (Fig. 2 B & C), which also have more long-term avoided permafrost carbon loss compared to the higher-emission SSP scenarios (discussed below).

In the Discussion, we have added the following text:

(Added text to the Discussion): Another consideration is that the proportionality found here is a function of the UVic-ESCM's TCRE given that the fundamental proportionality between carbon storage-years and degree-years of avoided warming arises directly from TCRE linearity; a model with a different TCRE value should simulate a different magnitude of proportionality during the period of active storage-years (i.e., up to 2100). Additionally, differences in ZEC values among models would change the relationship between storage-years and changes occurring after storage-years stop accumulating (i.e., from 2100 to 2300). We expect that our conclusion of proportionality at the year 2100 would hold across other models given the consistent linearity of the TCRE relationship among models, but that changes after 2100 would be more sensitive to differences in ZEC across models. Future multi-model intercomparisons would be an important next step to verify whether this storage-year proportionality remains robust across different climate model structures and complexities.

Fig. 6: isnt this the same as SM Fig. 5? And given this figure, can't you omit Fig. 4, whose info is contained in here?

We thank Reviewer 1 for pointing out that we had mistakenly had the same figure in both the SM and the main manuscript – we have corrected this by removing SM Fig. 5.

Reviewer 1 has pointed out that the Fig. 6 and Fig. 4 are similar, and they have suggested that because of this similarity it would make sense to remove Fig. 4 altogether. We would argue that keep the information from both figures is important to show how the proportional relationships change from 2100 to 2300. However, to make the changes between 2100 and 2300 more comparable, we have removed the original versions of Fig. 4 and Fig. 6 from the manuscript. In response to this comment and to other comments by other reviewers, we have also separated the original contents of Fig. 4 and 6 based on which variables demonstrate scenario dependent proportionality (AMOC and permafrost carbon) vs. scenario independent proportionality (ocean temperature, total carbon, and relative sea-level height).

(Revised Figures 4 and 6 – Figures 5 and 6 in the revised manuscript):

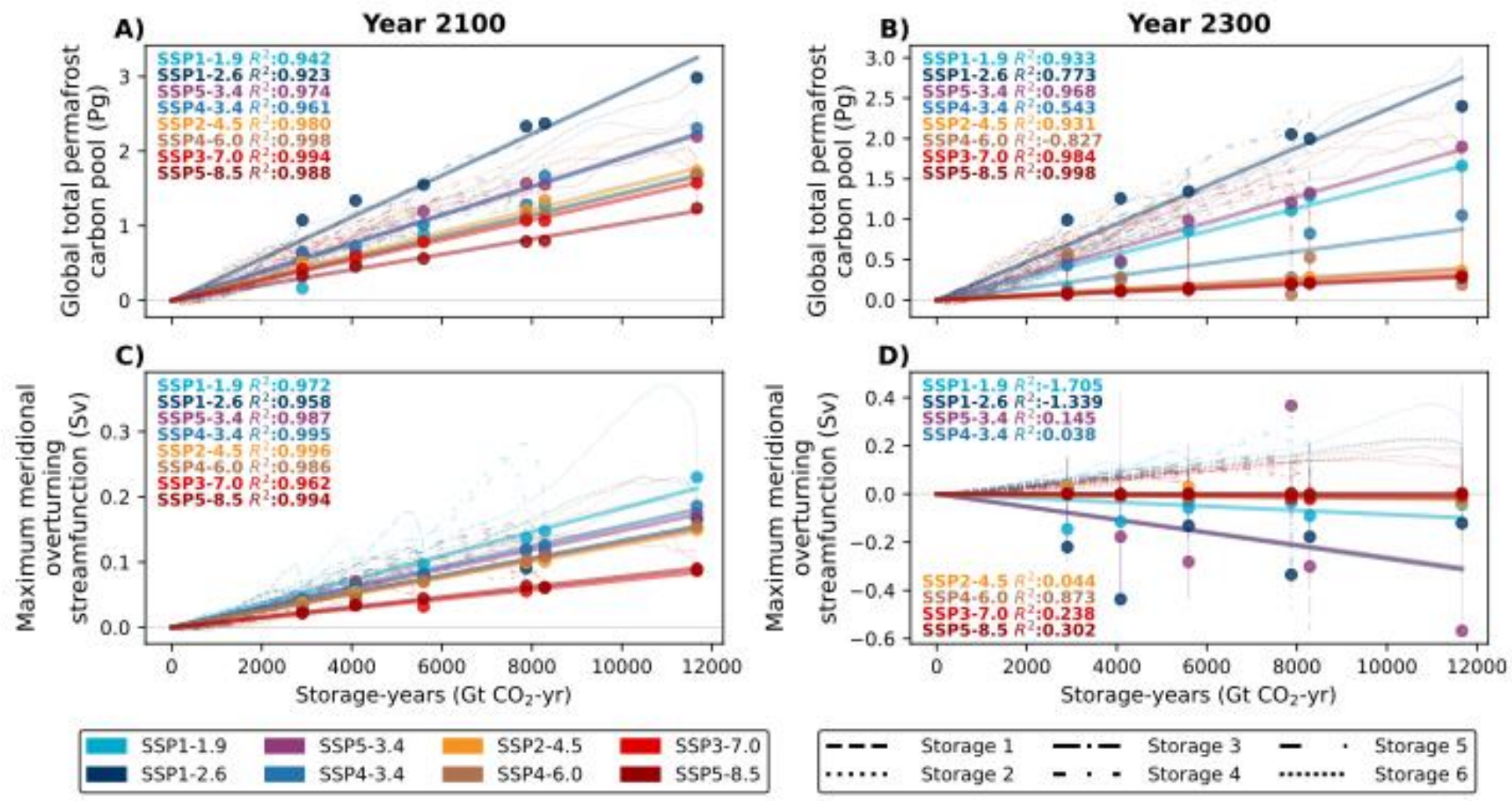

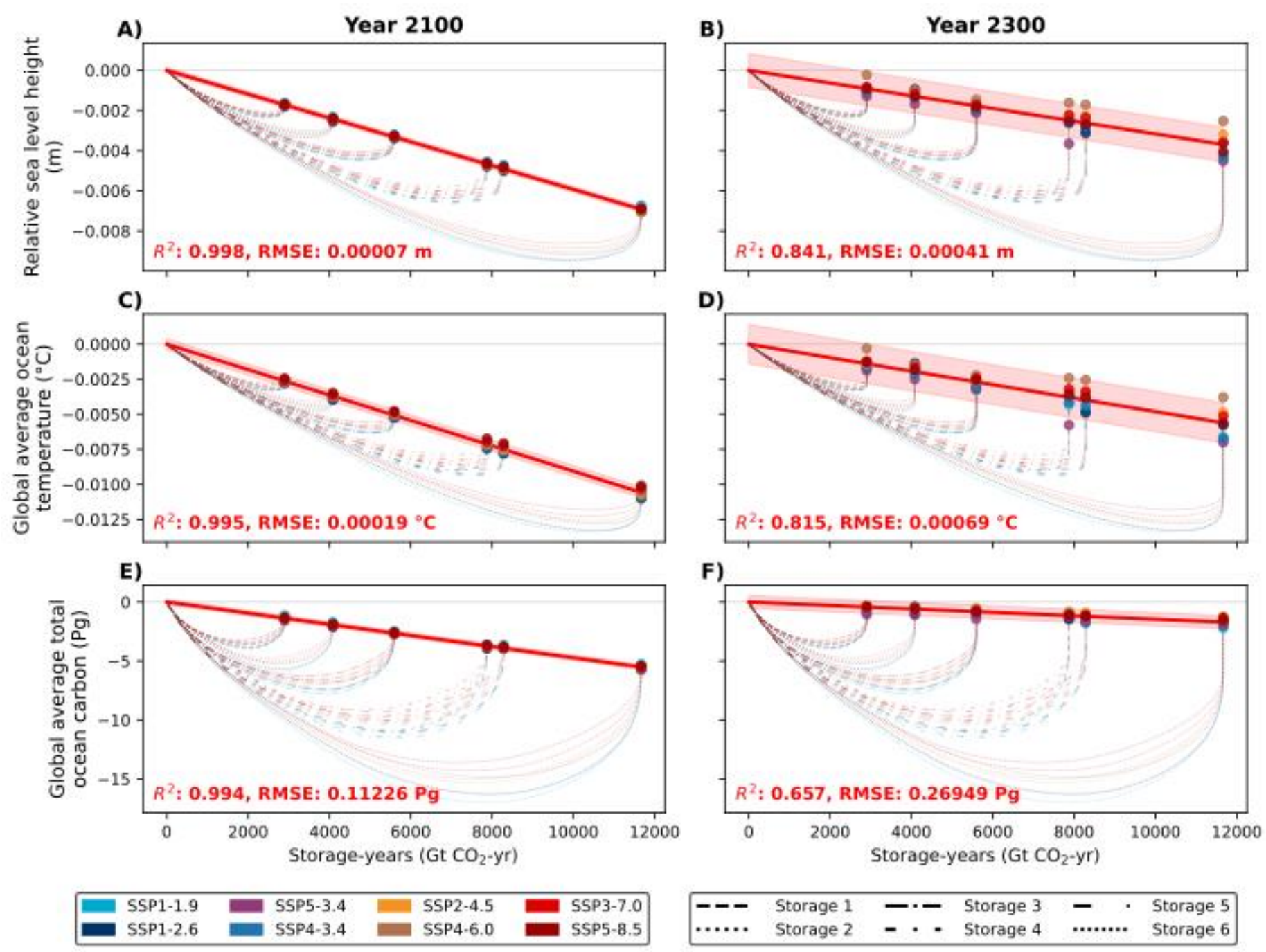

Line 264: Fix the wrong caption text "Figure 4)".

This typo has been corrected.

Line 290-292: Could this be because AMOC transitions through an edge state and initial conditions determine more than small variations in forcing, how it evolves? In this light, how reliable are these findings against internal variability?

We thank the reviewer for this insightful comment. The AMOC response in the UVic-ESCM is not stochastic, so a simulation with the same forcings, initial conditions, and model setup would reproduce the same AMOC response. The highly variable and divergent multi-century AMOC

response, characterized by both positive and negative anomalies under peak-and-decline pathways, is thus driven by a high sensitivity to small scenario-specific variations and internal variability. This behavioral characteristic directly explains why the storage-year scaling approximation breaks down for the long-term AMOC response, whereas it remains robust for slow-responding stock variables examined here. We have updated the manuscript text corresponding to explicitly acknowledge this sensitivity to internal variability.

> *(Original Lines 290-292): As the only flux variable considered here, the difference in the multi-century responses of the AMOC between storage and baseline SSPs are more uncertain than the other stock variables that we included in our analysis.*
>
> *(Revised): As a dynamic flux variable, the AMOC is sensitive to internal variability, which likely drives this scenario divergence and results in greater long-term uncertainty relative to the stock variables examined here.*

Line 304-306: omit, that is stated above already.

We would like to clarify that these two passages address distinct points: the earlier text discusses the physical breakdown of proportionality between storage-years and the multi-century AMOC response, whereas this section explains how that breakdown specifically impacts the relative mitigated change metric, causing it to fluctuate and become difficult to interpret. To clear up any perceived redundancy and make this connection explicit, we have revised the text to directly link the metric's variability to the underlying breakdown of proportionality:

> *(Original): For the AMOC, the relative avoided change in peak-and-decline SSPs by 2300 is highly variable, exhibiting both positive and negative responses change across scenarios, making it difficult to interpret (SM Fig. 6).*
>
> *(Revised): For the AMOC, the relative avoided change in peak-and-decline SSPs by 2300 is highly variable, exhibiting both positive and negative responses across scenarios (Fig. 7 B). This divergence reflects the breakdown of proportionality between storage-years and the multi-century AMOC response, driven by high flux variability by 2300.*

Line 337-339: this is to be shown with different scenarios of different storage times. Otherwise this is only shown across storage magnitude.

We have revised these lines to clarify that these results are specific to our simulations:

> *(Original): Despite these complexities, the robust relationship we identify between carbon storage-years and avoided changes in inertial variables provides a clear metric for valuing non-permanent sequestration.*
>
> *(Revised): Despite these complexities, the robust relationship we identify between carbon storage-years and avoided changes in some inertial variables provides a potential mechanism to value non-permanent sequestration <u>across the storage magnitudes we explore here.</u>*

Line 399-401: please make explicit where the model's limitations lie for this study, which conclusions you expect to be equally drawn from findings reproduced by other models.

We thank the reviewer for this insightful comment. We have revised the Discussion to make the conclusions we expect to be reproduced in other models more explicit. Because the core quasi-linear scaling of our framework is physically rooted in the robust near-proportionality between cumulative emissions and global temperature change, which is found across models, we fully expect the qualitative property of storage-year proportionality to be reproduced across other Earth system models. Conversely, the slope of the proportionality and the multi-century recovery pathways are inherently model-dependent limitations bounded in part by the specific TCRE and ZEC characteristics of our particular framework. We also discuss how the results in a full-complexity climate model may change as a result of additional internal variability compared to what is simulated in the UVic-ESCM. This added context has been integrated into the Discussion to provide a more transparent and balanced boundary for our conclusions.

> *(Added text to the Discussion): This proportionality may also be subject to the specific characteristics of the UVic-ESCM. A key structural characteristic of the UVic-ESCM is its single-layer atmosphere and prescribed atmospheric circulation, which prevents the model from generating significant interannual atmospheric variability. The response of a full-complexity model would be additionally affected by unforced decadal atmospheric variability which could introduce greater scenario dependence. Another consideration is that the proportionality found here is a function of the UVic-ESCM's TCRE given that the fundamental proportionality between carbon storage-years and degree-years of avoided warming arises directly from TCRE linearity; a model with a different TCRE value should simulate a different magnitude of proportionality during the period of active storage-years (i.e., up to 2100). Additionally, differences in ZEC values among models would change the relationship between storage-years and changes occurring after storage-years stop accumulating (i.e., from 2100 to 2300). We expect that our conclusion of proportionality at the year 2100 would hold across other models given the consistent linearity of the TCRE relationship among models, but that changes after 2100 would be more sensitive to differences in ZEC across models. Future multi-model intercomparisons would be an important next step to verify whether this storage-year proportionality remains robust across different climate model structures and complexities.*

Fig. SM 4: In particular, I think SM Fig. 4 shows important features of benefit decay that deserve the attention of the main manuscript readers to allow for their transparent discussion. Moreover, a figure illustrating the relative longterm share of beneficial effect would help the understanding. E.g. renormalize Fig. SM 4 to the peak effect (typically around 2060), so that e.g. global average total ocean carbon under Storage 6 would decay to 5% toward 2300, ocean temperature to 40-50%.

Following Reviewer 1's earlier suggestions, we have used a broken axis to show the evolution of the effect of temporary storage up until 2300 in the main manuscript in Fig. 4 to highlight the

benefit decay on a centennial timescale. We have also made an additional supplementary figure showing the normalized effect of temporary storage, with this effect in variable being normalized to the maximum difference between each storage and baseline SSP pair observed in the 21st century. We have also added a brief discussion of the figure in the results.

> *(Added text to the Results): A corresponding version of Figure 4 that is normalized to the maximum difference between storage SSPs and their relative baselines in the 21st century is provided in the Supplementary Information (Supplementary Fig. 3). This normalization demonstrates that the climate benefit of temporary storage by 2300 generally lower than its peak benefit in the 21st century. Ocean temperature and sea-level height retain ~50% of their benefits, whereas total ocean carbon retains only ~10-25%. For permafrost carbon, peak-and-decline SSPs generally retain most of the benefit of temporary storage, while high-emissions SSPs generally see decreased benefit over time. AMOC changes after 2100 are highly variable, with no consistent relationship with storage-years at the year 2300.*

> *(New Supplementary Figure 3)*

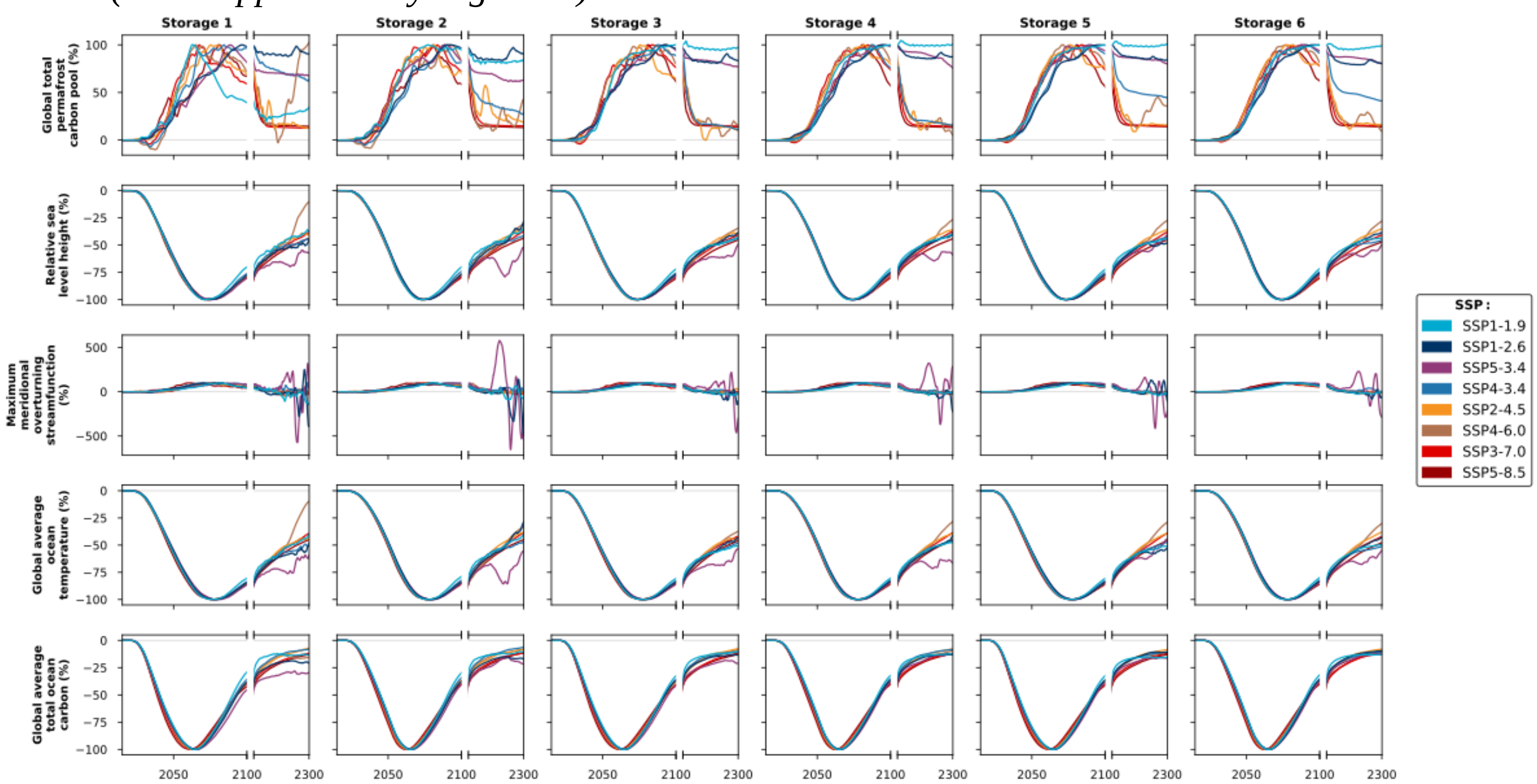

Reviewer #2 (Remarks to the Author):

I do not believe that the results presented in this paper are novel or noteworthy enough to be published in this journal. Many papers have been published in recent decades discussing the potential impacts of temporary carbon storage. The authors do not clearly explain how their work contributes to existing knowledge. Furthermore, one of the main limitations identified in the literature is that including altered albedo effects could significantly alter the conclusions regarding land carbon storage. The authors chose not to include these effects in their study. Including these effects would have produced more novel and, more importantly, more accurate conclusions. See below for more specific comments.

We thank Reviewer 2 for their overview and critical assessment of our manuscript. While we recognize that previous literature has discussed the general implications of temporary carbon storage on global mean temperature, our study offers a distinct contribution by establishing a quasi-linear relationship between storage-years and the multi-century mitigation of highly inertial, slow-responding Earth system components.

We agree that biogeophysical feedbacks, such as altered surface albedo, are essential for evaluating localized land-management projects. However, our study was explicitly designed to isolate the fundamental biogeochemical response of the climate-carbon system to temporary CO_2 perturbations. To clarify this boundary and ensure our results are not overgeneralized, we have revised the title, standardized our terminology around "temporary carbon storage" rather than specific land-based solutions, and fundamentally restructured our methodology using a common "removal-neutral" baseline (SSP3-7.0 land-use emissions).

We believe these substantial revisions significantly strengthen the clarity, realism, and robustness of our findings, and we have provided detailed responses to your individual points addressed below.

Figure 1: Adding a horizontal line at y=0 on graphs would help as we could better see when results become negative. Figure 1D is very difficult to read because there are too many curves and several of them overlap. I cannot really tell which set of curves represents storage 1 vs. storage 2, etc. In other words, I do not see if storage 1 is better than storage 2, etc.

We have added horizontal lines at y=0 to subplots A and B in Figure 1 to more clearly distinguish between positive and negative climate responses. In subplot F (formerly subplot D), we have reduced the line thickness to improve legibility. We argue, however, that the overlapping lines in this subplot do not hinder the interpretation of the results, as the main illustrating point is that temperature returns to within a tenth of a degree of the baseline SSPs for almost all storage SSPs, with the main exception of the high-overshoot SSP5-3.4OS scenario. This finding remains clearly discernible across the ensemble regardless of the overlapping curves.

Here is the revised Figure 1:

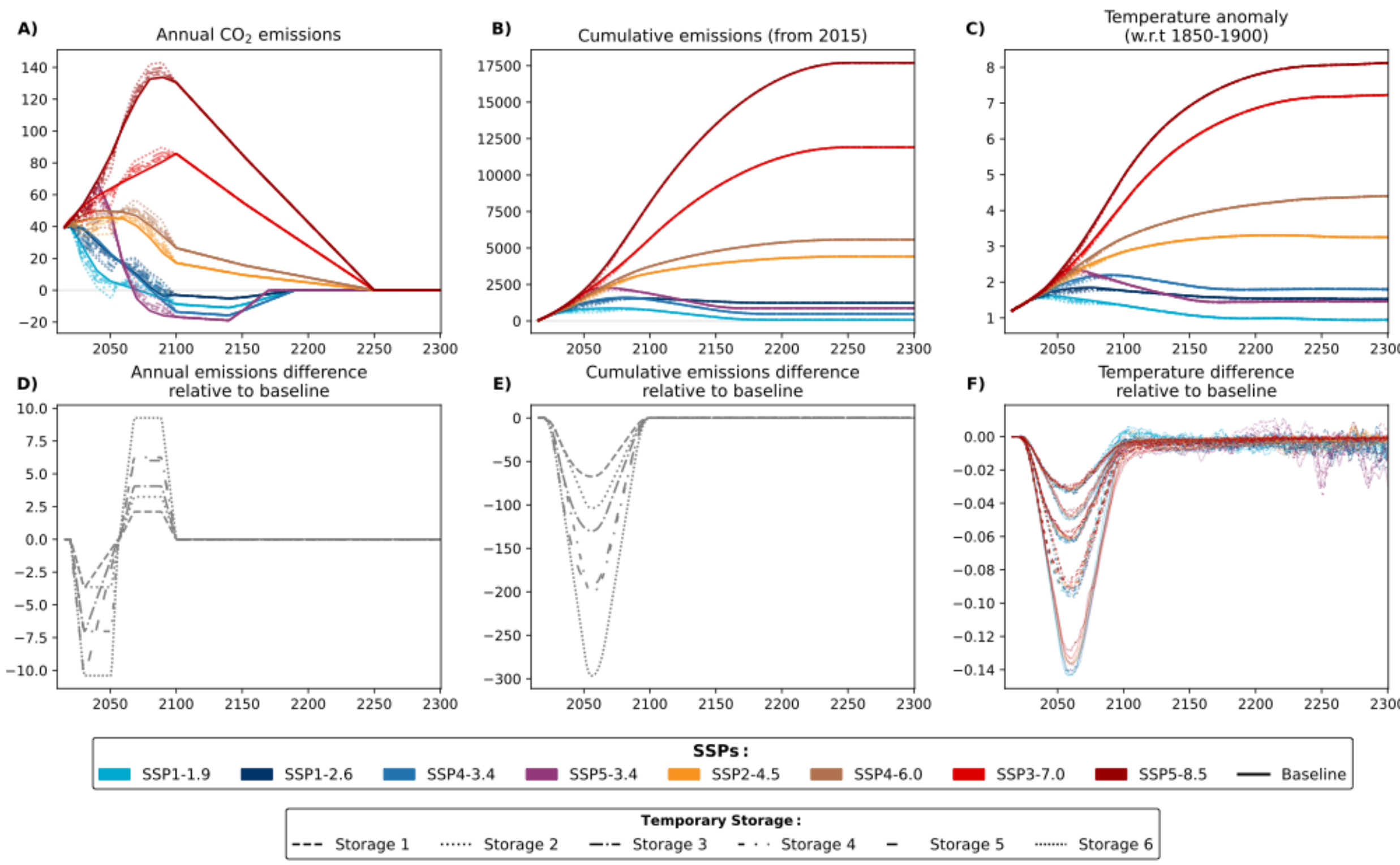

Methods – Scenario design: It would be helpful to have a figure showing these six temporary carbon storage pathways. It would also be useful to justify the selection of the two temporal profiles. While maximum rates of sequestration were justified in the introduction, the two distinct pathways described in the Methods section were not.

We agree with Reviewer 2 and have added subplot D to Figure 1 showing the six temporary carbon storage pathways – see revised Figure 1 pasted above.

We have also added the following text justifying our use of two temporal profiles in the Methods:

> *(Text added to Methods): Using two distinct temporal trajectories – one with a linear ramp and one with sustained maximum removal – allows us to test the robustness of the storage-year proportionality framework across pathways with different timing of sequestration and re-emission.*

Methods – Simulation design: "By modulating prescribed land-use CO2 emissions rather than explicitly including the land-use and land-cover changes that would accompany many forms of nature-based carbon removal, our results reflect the biogeochemical effect of temporary carbon storage but not biogeophysical effects such as altered surface albedo or cloud cover." This is a significant limitation that invalidates the study's conclusions. As the literature shows, altered surface albedo can cause global temperature effects that are of the same order of magnitude as stored carbon, but opposite in sign. Therefore, the title of this paper, "Temporary land carbon storage can avoid slow-responding climate changes", may

not be true if this temporary land carbon storage occurs through afforestation projects in snow-covered regions, for example.

We acknowledge Reviewer 2's assertion that biogeophysical effects like albedo are significant, as noted in the original manuscript:

> *(Lines 326-329 of the original manuscript) Any real-world interpretation of these findings must consider the simulation design of our study. Most notably, our simulations isolate the biogeochemical response to temporary storage and do not account for the additional biogeophysical, non-local, or non-CO_2 effects that can be associated with carbon removal.*

However, we disagree that excluding these effects invalidates our conclusions. Standard scientific practice involves isolating specific mechanisms to evaluate their individual contributions. This study isolates the biogeochemical response to characterize the relationship between temporary carbon storage and slow-responding variables. This approach ensures the findings remain applicable to various carbon dioxide removal methods. We have revised the title to "Temporary carbon storage can mitigate slow-responding climate changes" – i.e., removing "land" from "Temporary land carbon storage…" to reflect this broader scope. Additionally, per Reviewer 1's suggestion, we have also replaced most instances of "nature-based" in the introduction with "temporary carbon storage" to focus on carbon-cycle physics rather than specific land-system interventions.

Additionally, we have added to the following example of how the inclusion of the biogeophysical effects may change the results of our study:

> *(Added text to the Discussion): For instance, if temporary storage is achieved via high-latitude reforestation or afforestation, localized biophysical feedbacks, such as reduced surface albedo, could counteract biogeochemical cooling and lower the slope of the storage-years relationship with avoided inertial climate changes*

Results - Lines 114-147:
As stated by the authors (lines 132-136), the quasi-linearity between storage-years of temporary carbon storage and degree-years of avoided warming has already been demonstrated. These findings are not novel.

While previous research established a quasi-linear relationship between temporary storage and climate response, that evidence was limited to two mitigation scenarios (SSP1-1.9 and SSP1-2.6) and two temporary storage pathways[1]. Our study demonstrates that this quasi-linearity remains consistent across the eight baseline SSP scenarios, representing a much wider range of end-of-century warming, and across six distinct temporary carbon removal profiles. By confirming these relationships across a significantly broader ensemble of emissions intensities and sequestration trajectories, our results establish the storage-year framework as a more robust and generalizable property of the Earth system.

In the original manuscript, we explicitly state the difference between our finding of quasi-linearity between storage-years of temporary carbon storage and degree-years of avoided warming and that of previous work:

> *(Original manuscript lines 133-136): Consistent with previous work, we find a quasi-linear relationship between storage-years of temporary carbon removal and degree-years of avoided warming in our simulations (Fig. 2 B), across a wider range of SSPs and temporary storage pathways as compared to previous work.*

[1] Matthews, H.D., Zickfeld, K., Koch, A. et al. Accounting for the climate benefit of temporary carbon storage in nature. Nat Commun 14, 5485 (2023). https://doi.org/10.1038/s41467-023-41242-5

"This proportionality indicates that the duration and magnitude of carbon storage directly determine the duration and magnitude of avoided warming." This is true when storage-years and degree-years are calculated for a time horizon that corresponds to the storage/release period (up until 2100 in this case). But what happens if we go beyond this time horizon?

Several papers have been published in the last 25 years regarding the impact of temporary carbon storage. I feel that this section simply repeats what has already been shown without referencing this literature. Furthermore, it fails to show what will happen on a longer timescale after 2100, as temporary carbon storage may simply displace future warming effects.

We agree that evaluating the climate response beyond the 2100 time horizon is essential for understanding the long-term impacts of temporary carbon storage. Our original manuscript included simulations and analysis of the storage-year vs. degree-year relationship up to the year 2300 in the section "MULTI-CENTURY RESPONSE TO TEMPORARY CARBON STORAGE" lines 242-249, as well as in SM Figure 3 in the original submission. In response to this feedback, and in response to the suggestion from Reviewer 1 to place a more equal emphasis on the 2100 and 2300 results, we have restructured the manuscript to balance the 2100 and 2300 results more equally. A revised Figure 2 has been updated to include the relationship between storage-years and degree-years through 2300 in the main manuscript rather than just in the SM as in the original submission, providing a clear visualization of how these benefits persist or evolve on multi-century timescales.

The revised Figure 2 showing the relationship between storage-years and degree-years until 2300:

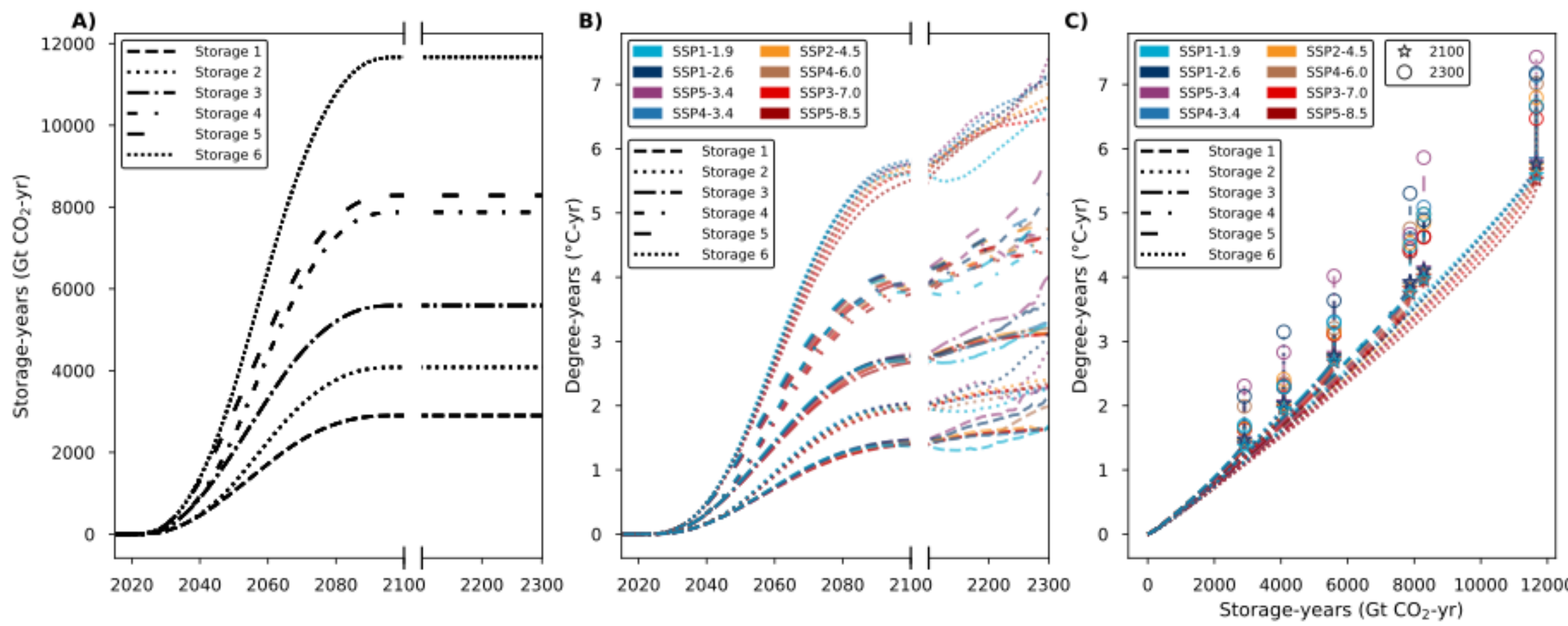

Results – Lines 152-167:
"Unlike the effects of temporary carbon storage on temperature, slow-responding climate variables show persistent avoided changes that are not reversed once temporary carbon storage is re-emitted by 2100 (Fig. 3)." Since Figure 3 ends at 2100, it is unclear whether these changes are persistent. This conclusion is biased.

We thank Reviewer 2 for pointing out that the temporal scope of the original Figure 3 may have obscured the persistence of the mitigation signal. The original statement was intended to highlight that, unlike global temperature, which returns to baseline levels almost immediately upon re-emission, slow-responding variables retain a measurable benefit at the year 2100 – the point at which all temporarily stored carbon has already been returned to the atmosphere in our simulations.

However, we would like to point out that the extended version of Figure 3 (showing changes until 2300) was included in the original supplementary information as Figure 4 and was discussed in the results section "MULTI-CENTURY RESPONSE TO TEMPORARY CARBON STORAGE" (lines 250-260). Furthermore, the original Figure 3 demonstrated that a persistent effect exists in 2100, and the original SM Figure 4 showed that this effect persists until 2300 for all variables except for AMOC.

To address Reviewer 2's concern regarding bias, we have extended Figure 3 to the year 2300 so that this data is visualized in the main manuscript rather than just in the supplementary information. We have also modified the sentence in question to more clearly highlight that we are discussing the climate response in 2100:

> *(Original): Unlike the effects of temporary carbon storage on temperature, slow-responding climate variables show persistent avoided changes that are not reversed once temporary carbon storage is re-emitted by 2100 (Fig. 3).*

> *(Revised): Unlike the effects of temporary carbon storage on temperature, slow-responding climate variables show persistent avoided changes in 2100 despite temporary carbon storage having already been re-emitted by 2100 (Fig. 4).*

The revised Figure 3 (Now Figure 4) and the updated text, combined with the multi-century analysis already present in our results, provide transparent evidence of how these benefits evolve or decay over three centuries.

Results – Lines 213-226:
What do these results mean? How is this useful to know that?

We thank Reviewer 2 for the opportunity to clarify the purpose and utility of this analysis. While our earlier results establish the physical proportionality between storage-years and avoided climate change, the analysis in this section (represented in the original Figure 5) was designed to contextualize the relative impact of these benefits against the total magnitude of climate change expected in each SSP. This is relevant for climate policy and mitigation planning for two reasons:

1) *Assessing Mitigation Efficacy: It demonstrates that the relative benefit of temporary carbon storage is significantly higher when paired with ambitious mitigation (e.g., up to ~10% avoided permafrost carbon loss in SSP1-1.9) than in high-emission scenarios where its relative contribution is negligible (0% to 2.5%).*
2) *Strategic Planning: These results provide a framework for decision-makers to evaluate where temporary carbon storage might offer the most "value". The results reinforce the conclusion that temporary storage is a valuable complement to, but not a substitute for, deep fossil fuel emissions reductions, as its relative efficacy diminishes if background emissions remain high.*

Discussion – Lines 314-315: "For temperature, temporary storage can lower peak warming when combined with ambitious mitigation, which previous work shows would reduce the risk for triggering climate tipping points." Since this study does not include altered albedo effects, we do not know. This claim may be incorrect.

We acknowledge Reviewer 2's point that biogeophysical effects like altered surface albedo can influence the net climate impact of nature-based mitigation. However, as we mention above, we disagree that excluding these effects invalidates our conclusions, as standard scientific practice involves isolating specific mechanisms – in this case, the biogeochemical response – to evaluate their individual contributions to climate dynamics. Our approach ensures that our findings are applicable to the biogeochemical effects of various carbon dioxide removal methods, including but not limited to nature-based methods. To clarify this scope, we have updated the title to "Temporary carbon storage can mitigate slow-responding climate changes," removing the reference to "land" to better reflect the focus on carbon-cycle physics. We have also substituted "nature-based" to "temporary carbon storage" terminology to emphasize the general climate-carbon system response to CO_2 perturbations. As we formerly mention, we would like to highlight that in the Discussion section of the original manuscript explicitly, we state that our results only speak to the biogeochemical response to temporary carbon storage:

(Lines 326-329 of the original manuscript)" "Any real-world interpretation of these findings must consider the simulation design of our study. Most notably, our simulations isolate the biogeochemical response to temporary storage and do not account for the additional biogeophysical, non-local, or non-CO_2 effects that can be associated with carbon removal."

To further clarify that our study addresses the biogeochemical effect of temporary carbon storage, we have also added text to be more specific about the scope of the study in the Introduction of the manuscript:

(Original): "These perturbations were used to approximate the effect of time-limited carbon storage interventions."
(Revised): "These perturbations were used to approximate the biogeochemical effect of time-limited carbon storage interventions."

We have also added text in the Discussion to emphasize that our results reflect the biogeochemical effect of temporary carbon storage:

(Original): Our results show that temporary carbon storage delivers measurable climate benefits that persist beyond the lifetime of the stored carbon.

(Revised): Our results show that the biogeochemical effect of temporary carbon storage delivers measurable climate benefits that persist beyond the lifetime of the stored carbon.

Finally, we have added an example to the Discussion that demonstrates how the inclusion of biogeophysical impacts of temporary carbon storage may change the results:

(Added text to the Discussion): For instance, if temporary storage is achieved via high-latitude reforestation or afforestation, localized biophysical feedbacks, such as reduced surface albedo, could counteract biogeochemical cooling and lower the slope of the storage-years relationship with avoided inertial climate changes

Finally, previous research evaluating the combined biogeochemical and albedo impacts of non-permanent sequestration from afforestation and reforestation shows that while surface albedo changes can diminish net cooling, the core physical relationship between temporary storage and temperature change is preserved [1]. This indicates that the functional proportionality of the storage-years framework may remain structurally robust, even if localized biophysical feedbacks such as albedo alter the specific magnitude of the regression slopes.

[1] Matthews, H.D., Zickfeld, K., Dickau, M. et al. Temporary nature-based carbon removal can lower peak warming in a well-below 2 °C scenario. Commun Earth Environ 3, 65 (2022). https://doi.org/10.1038/s43247-022-00391-z

Discussion – Lines 330-331: "In scenarios such as SSP1-1.9 and SSP1-2.6, which already rely on significant carbon dioxide removal to meet temperature targets, the addition of

further storage may be unrealistic from both physical and socioeconomic perspectives." This is very important. These conclusions may be useless due to this limitation.

We agree with Reviewer 2 that the physical and socioeconomic realism of carbon storage pathways is a critical factor for the study's broader applicability. In response to this feedback, we have revised our methodology by standardizing the land-use CO_2 emissions baseline across all SSP scenarios to follow land-use emissions from the no-policy scenario SSP3-7.0. In our original submission, stacking temporary storage onto baselines like SSP1-1.9, which already incorporate significant carbon removal, could result in unrealistic rates of removal. By now adopting the land-use emissions of SSP3-7.0 – the only SSP where these emissions remain positive throughout the 21st century – as a common "removal-neutral" baseline, we ensure that our temporary storage perturbations do not exceed plausible sequestration limits. This methodological change confirms that the identified proportionality between storage-years and avoided climate change is a robust property of the climate-carbon system, rather than an artifact of potentially unrealistic removal capacity assumptions. We believe these improvements directly resolve Reviewer 2's concern.

These methodological changes are now discussed in the Methods section:

> *(Added text to the Methods): To ensure carbon storage pathways were applied to a consistent baseline, we standardized land-use CO2 emissions across all SSP scenarios. Ambitious mitigation SSPs (e.g., SSP1-1.9 or SSP1-2.6) already include significant land-based carbon removal. Therefore, adding large further storage perturbations to these SSPs would likely produce carbon removal pathways that are quite unrealistic from a technical, social, and/or economic sense. In recognition of this, we replaced the land-use CO2 emissions of every SSP with values from the SSP3-7.0 scenario because it is the only SSP where land-use CO2 emissions remain positive throughout the 21st century. This provides a neutral baseline for applying temporary and permanent storage pathways. Aside from SSP3-7.0, this standardization alters the total cumulative CO2 emissions and resulting 2100 temperatures for all baseline SSPs. All other forcings (fossil fuel emissions, non-CO2 greenhouse gases, and aerosols) remain specific to their original SSP definitions.*

Lines 348-349: "Our results also demonstrate that the climate benefits of temporary carbon storage increase when paired with ambitious emissions reductions but diminish substantially in scenarios where emissions continue to rise." Since these temporary carbon storage pathways are probably not realistic under the most ambitious scenarios, as discussed in lines 330-331, and their benefits decrease under less ambitious reduction scenarios, as discussed in lines 348-349, the conclusion of the study is incorrect.

As mentioned above, to address the concern regarding scenario realism of the highly ambitious SSPs 1-1.9 and 1-2.6, we have updated our methodology to utilize a "removal-neutral" baseline (SSP3-7.0 land-use emissions across all baseline scenarios), which ensures that experimental storage perturbations remain within physically plausible limits across all mitigation pathways. Our result demonstrating that benefits are maximized under ambitious mitigation remains

unchanged in our new simulations with this improved baseline.

Reviewer #3 (Remarks to the Author):

This manuscript examines the climatic implications of temporary land carbon storage, arguing that the time-integrated amount of stored carbon ("carbon storage-years") scales approximately linearly with avoided changes in several slow-responding climate variables, including ocean heat content, thermosteric sea-level rise, permafrost carbon loss, and AMOC strength. Using a set of idealized perturbation experiments within the UVic Earth System Climate Model, the authors explore how temporary carbon storage affects these slow components of the climate system across several emissions scenarios and storage magnitudes.

The topic is timely and potentially important. Temporary carbon storage is increasingly discussed in the context of land-based mitigation strategies, nature-based solutions, and carbon removal approaches. The manuscript provides an interesting perspective on how temporary carbon storage may influence slow components of the climate system through the inertia of the carbon–climate response.

We thank Reviewer 3 for their close reading of our manuscript and for recognizing that the topic of temporary carbon storage is "timely and potentially important". We appreciate their acknowledgment that our work provides an "interesting perspective" on how this storage influences the slow components of the climate system through the inertia of the carbon–climate response.

However, I have several concerns regarding novelty, framing, methodological scope, and interpretation of results, which should be addressed before the manuscript can be considered for publication.

In the sections below, we provide detailed responses to the reviewer's specific concerns regarding novelty, framing, interpretation of results, and methodological scope, which we believe have significantly strengthened the manuscript and clarified its contributions to the field.

1. Positioning within the broader literature on temporary carbon storage

The manuscript presents the storage-years framework largely within a relatively recent line of research. However, a broader literature has examined the climatic implications of temporary carbon sequestration and delayed emissions, including tonne-year approaches, storage-year metrics, and accounting methods for non-permanent sequestration.

Relevant earlier work includes IPCC (2000), Moura-Costa and Wilson, Lashof and Hare, Müller-Wenk and colleagues, Clift and colleagues, as well as industrial ecology and life-cycle assessment studies addressing temporary sequestration and delayed emissions (e.g., Brandao et al.). Standards such as PAS 2050 (BSI, 2008) also incorporate approaches to temporary carbon storage in product carbon footprinting.

Engaging more fully with this earlier literature would strengthen the manuscript by clarifying the conceptual lineage of the storage-years concept and by helping identify what

is genuinely new in the present contribution. In particular, earlier work has examined the climatic implications of temporary sequestration through concepts such as delayed emissions, tonne-year equivalence, and dynamic carbon accounting. These studies already explored the relationship between temporary storage and time-integrated climate responses, which makes it important to clarify precisely what conceptual advance the present work provides beyond extending these relationships to additional variables and scenarios.

At present, the manuscript risks giving the impression that the core conceptual insight — linking integrated temporary storage to climate response — is largely novel, whereas related ideas have been explored in several contexts for more than two decades.

We thank the reviewer for highlighting the broader literature on tonne-year approaches and dynamic carbon accounting. We agree that grounding our study within this previous literature strengthens the manuscript by clarifying the conceptual history of time-integrated metrics. To address this, we have revised the Introduction to explicitly incorporate these foundational studies and standards (including IPCC, 2000; Lashof & Hare, 1999, Moura-Costa & Wilson, 2000; BSI, 2008; Müller-Wenk & Brandão, 2010; Levasseur et al., 2012; Brandão et al., 2013), drawing a clear line from early equivalence concepts to contemporary applications. Furthermore, we have expanded our text to clarify our precise conceptual advance: our study expands this framework into full Earth system physics, which allows us to go beyond previous globally-averaged and conceptual analyses of time-integrated climate response to quantify the climate benefit of temporary storage with respect to its mitigation of specific impact-relevant slow-moving climate subsystems (such as permafrost carbon pools, thermosteric sea-level rise, and ocean circulation) across a wide array of future emission pathways.

Here are the relevant revisions:

> *(Original text in Introduction): "Efforts to formalize this contribution have demonstrated a consistent relationship between the magnitude and duration of temporary carbon storage and the effect of this storage on temperature, showing that time-integrated measures of storage capture this relationship more effectively than simple stock measures. Matthews et al. showed that temporary storage-years (a.k.a. tonne-years) of carbon, which represent the time integrated amount of annual additional carbon storage, are approximately proportional to degree-years of avoided warming, which represent the time-integrated amount of avoided warming.*
>
> *(Revised): "Efforts to formalize the effect of temporary carbon storage on temperature date back more than two decades, when storage-year (a.k.a. tonne-year) and equivalence-time metrics were first proposed to compare permanent and non-permanent carbon storage11–13. These concepts were subsequently adopted within industrial ecology and life-cycle assessment methodologies to develop dynamic carbon accounting frameworks14–17. More recent work has demonstrated a consistent relationship between the magnitude and duration of temporary carbon storage and the effect of this storage on temperature, and has reinforced that time-integrated measures of storage capture this relationship more effectively than simple stock measures18–23. Existing evaluations have*

quantified these relationships using analytical accounting formulations, localized ecosystem carbon-cycle models, and an intermediate-complexity climate model 18–23. Matthews et al.22 showed that temporary storage-years of carbon, which represent the time integrated amount of annual additional carbon storage, are approximately proportional to degree-years of avoided warming, which represent the time-integrated amount of avoided warming.

2. Framing as "temporary land carbon storage"

The manuscript is framed as an analysis of temporary land carbon storage, yet the experiments performed do not explicitly simulate land-use interventions or land-system dynamics. Instead, temporary storage is implemented by subtracting from and later adding to prescribed land-use CO2 emissions, while land cover remains fixed and biogeophysical effects are excluded.

As a result, the experiments effectively represent idealized atmospheric CO2 perturbations, rather than explicit land-based carbon storage systems. Real land-based carbon storage involves additional processes such as land-cover change, disturbance regimes, albedo effects, hydrological feedbacks, nutrient constraints, and non-CO2 emissions.

The current experiments therefore demonstrate a property of the climate–carbon system response to temporary CO2 perturbations, rather than the full climatic implications of temporary land carbon storage systems. This distinction should be made clearer in the manuscript, and the interpretation of the results should be adjusted accordingly.

We agree with Reviewer 3 that our experiments effectively represent idealized CO_2 emissions perturbations designed to isolate the biogeochemical response of the climate-carbon system. While we recognize that real-world nature-based storage or other CDR methods can involve complex second order effects, our study's intent was to characterize the biogeochemical relationship between temporary sequestration and slow-responding climate variables.

To address your concerns and clarify this distinction, we have made several key revisions:

- *Title Change: We updated the title to "Temporary carbon storage can mitigate slow-responding climate changes," removing the word "land" to better reflect the focus on carbon-cycle dynamics.*
- *Terminology Updates: We replaced numerous instances of "nature-based" and "land-based" with "temporary carbon storage" or "carbon storage" to emphasize that the results represent a general system response to CO_2 perturbations that are CDR method-agnostic (lines 65-75).*
- *Discussion of Scope: We would like to point out that in the Discussion section of the original submission we state that:*

  > *(Lines 326-329of the original manuscript) "Any real-world interpretation of these findings must consider the simulation design of our study. Most notably, our simulations isolate the biogeochemical response to temporary storage and do not*

account for the additional biogeophysical, non-local, or non-CO_2 effects that can be associated with carbon removal."

However, to further clarify the study's scope, we have also added text to be more specific about the scope of the study in the introduction of the manuscript:

(Original): "These perturbations were used to approximate the effect of time-limited carbon storage interventions."
(Revised – line 92): "These perturbations were used to approximate the biogeochemical effect of time-limited carbon storage interventions."

As mentioned above, we have also added text in the Discussion to emphasize that our results reflect the biogeochemical effect of temporary carbon storage:

(Original): Our results show that temporary carbon storage delivers measurable climate benefits that persist beyond the lifetime of the stored carbon.
(Revised): Our results show that the biogeochemical effect of temporary carbon storage can deliver measurable climate benefits that persist beyond the lifetime of the stored carbon

Finally, we have added the following text to the Discussion to give an example of how including the biogeophysical effects of temporary carbon storage could change our results:

(Added text to Discussion): For instance, if temporary storage is achieved via high-latitude reforestation or afforestation, localized biophysical feedbacks, such as reduced surface albedo, could counteract biogeochemical cooling and lower the slope of the storage-years relationship with avoided inertial climate changes.

We hope these adjustments clearly frame the work as an analysis of the climate-carbon system's response to temporary CO_2 changes rather than a comprehensive evaluation of specific nature-based mitigation systems.

3. Interpretation of the quasi-linear scaling

A central result of the manuscript is the quasi-linear relationship between storage-years and avoided changes in several slow-responding climate variables. This relationship is also consistent with the well-established near-proportionality between cumulative CO2 emissions and global mean temperature change, which implies that time-integrated carbon perturbations will tend to scale with integrated climate responses. While this relationship is interesting, part of the observed proportionality may arise from the underlying relationship between cumulative CO2 perturbations and global temperature change. Because storage-years effectively integrate the carbon perturbation over time, the scaling relationship may partly reflect the mathematical structure of the climate response to cumulative CO2 emissions rather than a distinct emergent property specific to temporary carbon storage.

Clarifying this point would help readers better understand the conceptual contribution of the analysis.

We agree with Reviewer 3 that the quasi-linear relationship between storage-years and mitigated climate changes is fundamentally consistent with the well-established near-proportionality between cumulative CO_2 emissions and global temperature change. Indeed, because storage-years represent the time-integrated carbon perturbation, the observed proportionality is rooted in the structure of the climate-carbon system's response to cumulative emissions. Reviewer 1 also raised this point in relation to how inter-model differences in TCRE and the Zero Emissions Commitment (ZEC) could affect our conclusions, and we have added additional discussion also in response to this reviewer comment.

We would like to point out that in the original submission we address how proportionality between degree-years of avoided warming and storage-years arises from the linearity of the TCRE:

> *(Original submission lines 138-147): This proportionality is grounded in the linear relationship between global mean temperature increase and cumulative CO_2 emissions, as described by the transient climate response to cumulative carbon emissions (TCRE). The linearity in the TCRE arises because each tonne of CO_2 produces a similar amount of warming regardless of when it is emitted or the background climate state, and because warming from CO2 persists for thousands of years. Consequently, a CO2 removal (or additional avoided emission) and an additional emission exert nearly equal but opposite effects on global temperature regardless of the timing of emissions or the background concentration of CO2. When time-integrated, this TCRE relationship extends to a proportionality between carbon storage-years and degree-years of avoided warming (Fig. 2 B).*

To further clarify this point, we have added the following text to the Discussion:

> *(Added text to the discussion): Another consideration is that the proportionality found here is a function of the UVic-ESCM's TCRE given that the fundamental proportionality between carbon storage-years and degree-years of avoided warming arises directly from TCRE linearity; a model with a different TCRE value should simulate a different magnitude of proportionality during the period of active storage-years (i.e., up to 2100). Additionally, differences in ZEC values among models would change the relationship between storage-years and changes occurring after storage-years stop accumulating (i.e., from 2100 to 2300). We expect that our conclusion of proportionality at the year 2100 would hold across other models given the consistent linearity of the TCRE relationship among models, but that changes after 2100 would be more sensitive to differences in ZEC across models. Future multi-model intercomparisons would be an important next step to verify whether this storage-year proportionality remains robust across different climate model structures and complexities.*

4. Sensitivity to storage timing

The climatic implications of temporary carbon storage depend strongly on when carbon is removed and when it is re-emitted relative to the trajectory of atmospheric CO2 concentrations and peak warming. In the present experiments, storage pathways appear to follow relatively structured uptake and re-emission patterns. It would be useful to clarify how sensitive the reported scaling relationships are to the assumed timing of storage and release, particularly relative to peak warming and net-zero transitions. Exploring alternative timing assumptions could help determine whether the observed scaling remains robust under different temporal structures of temporary storage.

We agree with Reviewer 3 that testing the temporal structure of carbon uptake and release is important for assessing the overall robustness of our findings. Our experimental design addresses this by evaluating two structurally distinct temporal profiles, and our results indicate that the long-term climate responses are not sensitive to the differences between these two pathways. However, in alignment with feedback from Reviewer 1, we have toned down our broader assertions regarding linearity (i.e., the linearity between storage-years and degree-years or the impact of temporary storage on slow responding variables) to clarify that our framework primarily evaluates quasi-linear scaling against the magnitude of carbon storage rather than against both the magnitude and duration of storage. While the consistency between our two tested pathways is encouraging, evaluating whether this linearity holds robustly across a wide variety of highly complex temporal patterns would warrant a comprehensive, standalone study on its own. We have updated the Discussion to more precisely delineate these boundaries of our scaling framework.

> *(Added text to the discussion): Furthermore, the storage rates applied here are highly idealized, which is an important consideration given that the efficacy of temporary storage appears to be partially contingent on the timing of sequestration and re-emission relative to peak warming. For instance, if temporary storage is re-emitted prior to net-zero CO2, as opposed to after net-zero CO2 as in this study for the peak-and-decline SSPs, storage may merely shift the peak's timing without reducing its magnitude. Therefore, while this work confirms that the proportionality between storage-years and avoided inertial climate changes holds across a wide range of storage magnitudes, a wider variety of temporal profiles of temporary carbon storage, in addition to the two temporal profiles simulated here, would have to be used to explore how well this proportionality holds across varying durations of temporary carbon storage.*

5. Robustness of the scaling across climate variables

While the scaling relationship appears relatively consistent for some variables (e.g., thermosteric sea-level rise and ocean temperature), it appears less robust for others.

In particular:
- Permafrost carbon responses show noticeable scenario dependence.
- AMOC responses appear highly variable, especially over longer time horizons.

These results suggest that the proportionality between storage-years and climate responses may not be universally robust across all slow components of the climate system. The manuscript would benefit from a more nuanced discussion of where the scaling appears robust and where it becomes less reliable.

We agree with Reviewer 3 that the robustness of the storage-year proportionality varies across different components of the climate system, reflecting their distinct physical response mechanisms. For example, while variables like ocean temperature and thermosteric sea-level rise scale reliably, permafrost carbon and AMOC responses exhibit notable scenario dependence. Specifically, the effect of temporary carbon storage on permafrost carbon loss is sensitive to path-dependent peak temperatures governed by the background emission pathway, and for the AMOC, the multi-century response is highly variable and there is less of a clear pattern.

In the original manuscript we did highlight how permafrost carbon and AMOC exhibit different responses in comparison to other variables:

> *(Lines 186-198 in the original manuscript): Permafrost carbon and the AMOC also scale proportionally with storage-years, but only within each SSP ensemble, with a different proportionality relationship for different baseline SSP scenarios (Fig. 4 A & C). This indicates that the relationship between storage-years and variable response is scenario dependent. For the AMOC, this dependence is closely linked to end-of-century warming: while the AMOC remains stronger relative to SSP baselines in all storage scenarios, the strength of the storage-year response declines in SSP ensembles with higher end-of-century warming (Fig. 4 C). For permafrost carbon, SSPs with greater warming generally show less benefit per storage-year of temporary storage, although this dependence is not explained by end-of-century warming alone. For example, SSP2-4.5 storage scenarios yield less permafrost carbon savings per storage-year than SSP4-6.0, while SSP4-3.4 and SSP5-3.4 yield more benefit per storage-year than SSP1-1.9 (Fig. 4 A). Instead, the scenario dependence of the permafrost carbon response is likely also influenced by the shape of the temperature pathway, such as differences in peak warming or the rate of warming leading up to 2100.*
>
> *(Lines 272-275 of the original manuscript): Despite this increasing scenario dependence by 2300, some proportionality between storage-years and avoided changes remains for thermosteric sea-level rise, ocean temperature, total ocean carbon, and permafrost carbon, the latter of which retains ensemble-level proportionality rather than proportionality across all SSPs (SM Fig. 5).*
>
> *(Lines 286-287 of the original manuscript): Multi-century changes in the AMOC behave differently from the other variable, with little proportionality between avoided change and storage years retained by 2300 (SM Fig. 5 C).*
>
> *(Lines 316-323 of the original manuscript): For the slow-responding variables examined here, temporary carbon storage reduces impacts by the end-of-century, with avoided changes by 2100 scaling proportionally with the magnitude and duration of storage,*

expressed in storage-years. For thermosteric sea level rise, ocean temperature, and total ocean carbon, this proportionality is independent of the SSP baseline. In contrast, the proportionality between storage-years and avoided change in the AMOC and permafrost carbon depends strongly on the baseline scenario, with more ambitious SSPs generally showing the largest avoided changes per storage-year.

To further clarify the difference in the responses of variables to temporary carbon storage (i.e., where this proportionality remains robust and breaks down), we have added the following text to the Discussion.

(Added text to the Discussion): Furthermore, the proportionality between storage-years and the climate response weakens by 2300, exhibiting clear divergence among slow-responding variables. Thermosteric sea-level rise, ocean temperature, and total ocean carbon retain scenario-independent proportionality to storage-years, whereas permafrost carbon only retains proportionality across temporary storage amounts within individual SSP groups (scenario-dependent proportionality). AMOC changes show high multi-century flux variability and therefore do not retain their proportionality with storage-year amounts on multi-centennial timescales.

In addition to this, we have added the following Discussion to help distinguish between the scenario independent proportionality and the scenario dependent proportionality found in the AMOC and permafrost carbon:

(Added text to Discussion): Because these scenario-dependent proportionalities are calculated within each SSP ensemble, each regression relies on only six data points. The proportionality relationships for AMOC and permafrost carbon are therefore inherently less robust than the scenario-independent proportionality derived from the fully aggregated multi-scenario dataset.

We have also restructured Figures 4 and 6 from the original manuscript showing the proportionality between storage-years and avoided change in 2100 and 2300. Now, the new restructured Figures (now number 5 and 6), are separated by variable type – variables that show scenario independent proportionality and scenario dependent proportionality.

(Restructured figures):

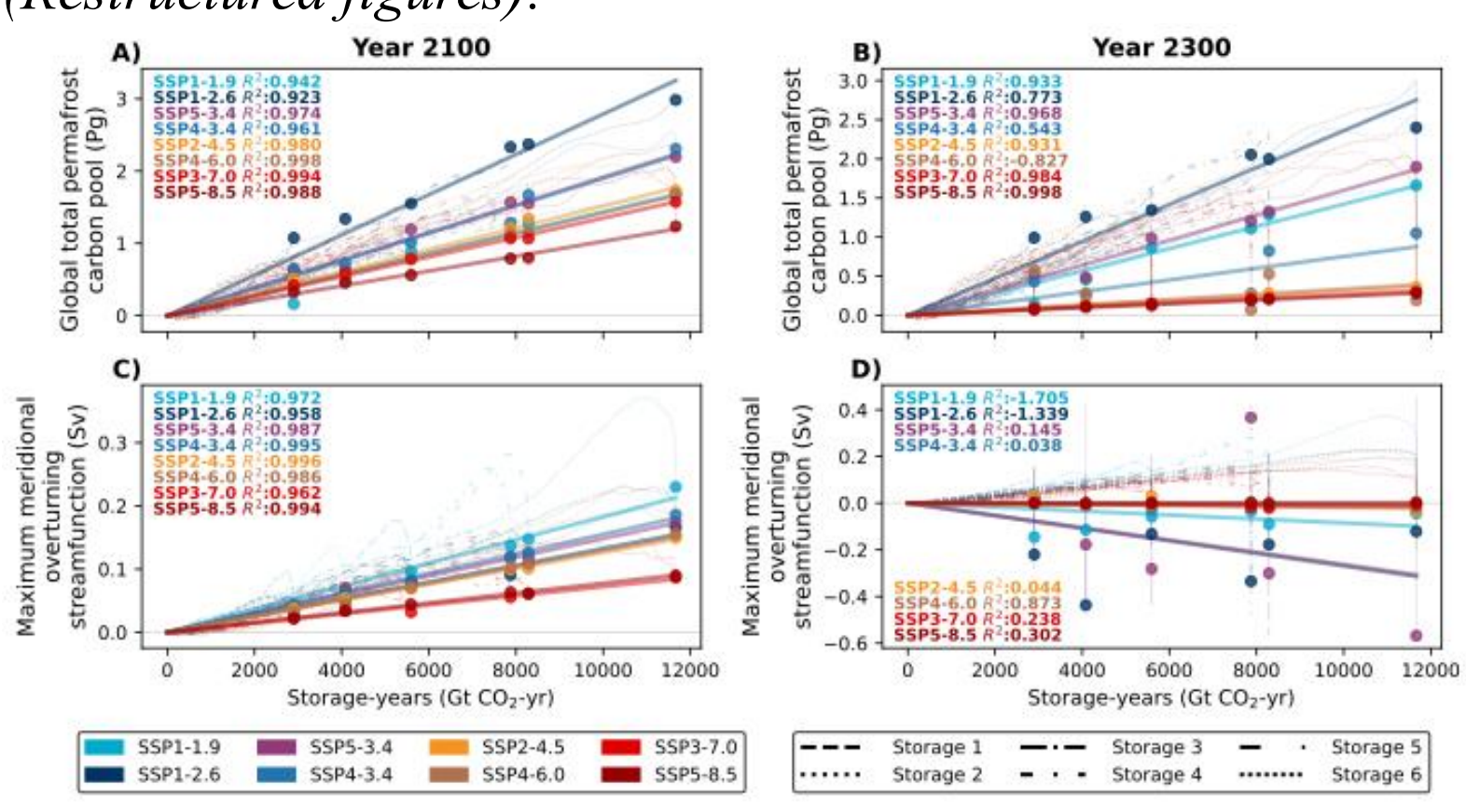

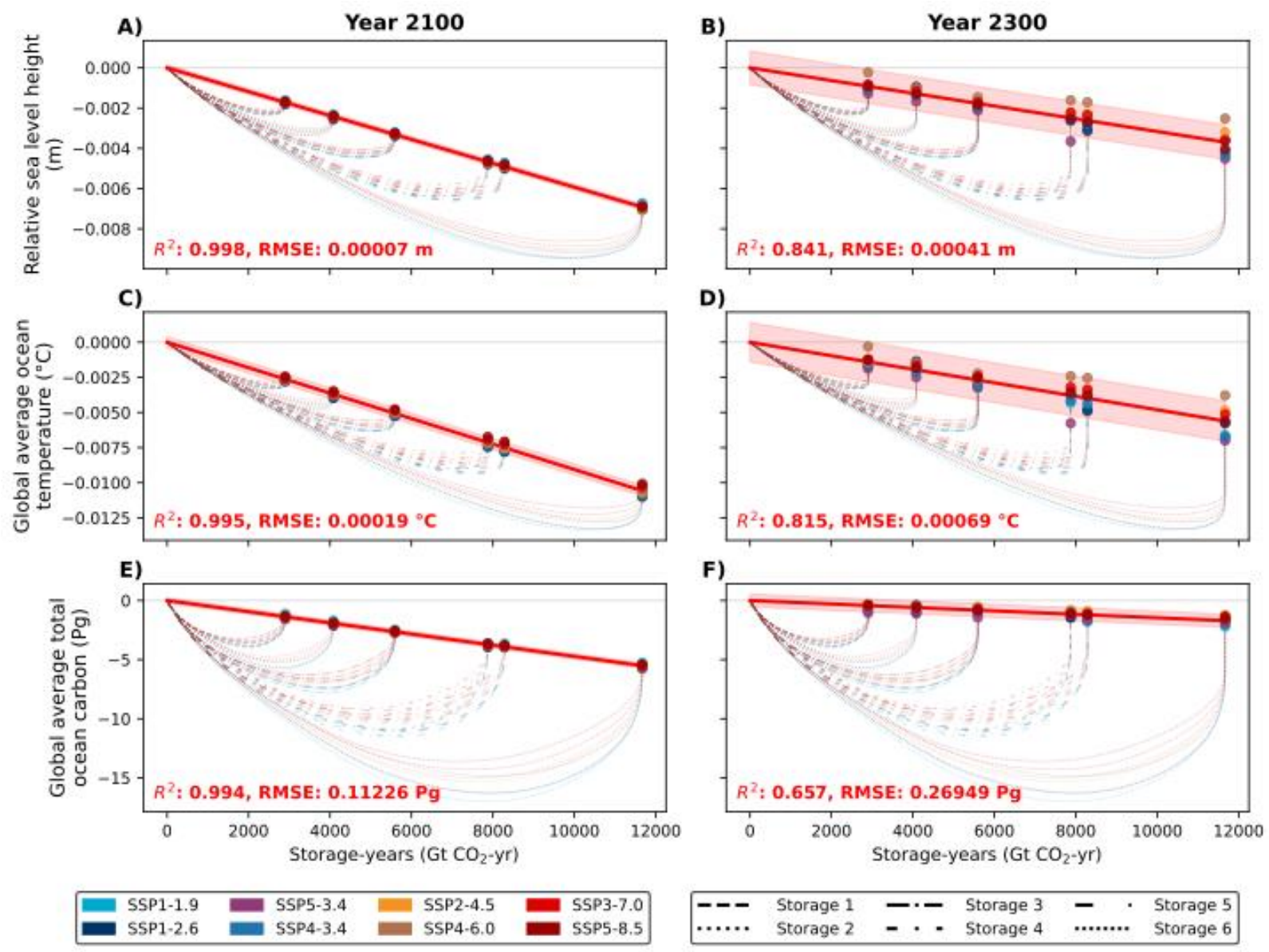

We believe these changes help clarify how AMOC and permafrost carbon behave differently than the globally averaged ocean variables examined here.

6. Dependence on a single Earth system model

All simulations are performed using UVic-ESCM, an intermediate-complexity Earth system model. While such models are valuable for conceptual experiments, conclusions regarding general properties of the climate system would ideally be supported by additional model evidence or uncertainty analysis.

At minimum, the manuscript should acknowledge more explicitly that the results represent model-based evidence from a single modelling framework, rather than a robust emergent property demonstrated across multiple climate models.

We agree with Reviewer 3 that we should be careful to over-generalize our results to the broader climate system because we use a single Earth system model of intermediate complexity (EMIC). The choice of an EMIC was a deliberate decision, as its computational efficiency was uniquely required to simulate a scenario ensemble of this scale and track multi-century responses up to the year 2300 across multiple emission pathways.

We accept that the proportionality found here represents model-based evidence within this specific framework rather than a universally proven multi-model emergent property. However, we note that the fundamental driver of this proportionality (the TCRE) is well established as a robust emergent property across climate models, and so we do expect our conclusions here to hold across other Earth-system models, subject to differences in the value of the TCRE across models. To address this, we have explicitly updated the Discussion section to acknowledge our reliance on a single modeling framework as a limitation, and we have added text emphasizing

that future multi-model intercomparisons are a necessary next step to verify the robustness of this proportionality across different model structures.

> *(Added text to the Dicsussion): This proportionality may also be subject to the specific characteristics of the UVic-ESCM. A key structural characteristic of the UVic-ESCM is its single-layer atmosphere and prescribed atmospheric circulation, which prevents the model from generating significant interannual atmospheric variability. The response of a full-complexity model would be additionally affected by unforced decadal atmospheric variability which could introduce greater scenario dependence. Another consideration is that the proportionality found here is a function of the UVic-ESCM's TCRE given that the fundamental proportionality between carbon storage-years and degree-years of avoided warming arises directly from TCRE linearity; a model with a different TCRE value should simulate a different magnitude of proportionality during the period of active storage-years (i.e., up to 2100). Additionally, differences in ZEC values among models would change the relationship between storage-years and changes occurring after storage-years stop accumulating (i.e., from 2100 to 2300). We expect that our conclusion of proportionality at the year 2100 would hold across other models given the consistent linearity of the TCRE relationship among models, but that changes after 2100 would be more sensitive to differences in ZEC across models. Future multi-model intercomparisons would be an important next step to verify whether this storage-year proportionality remains robust across different climate model structures and complexities.*

7. Interpretation for climate mitigation policy

The manuscript draws implications for the climate value of temporary carbon storage in mitigation portfolios and nature-based solutions. However, given the simplified experimental design and the absence of explicit land-system processes, the policy implications appear somewhat stronger than warranted by the analysis.

A more cautious discussion of the implications for real-world land-based mitigation strategies would improve the manuscript.

We agree with Reviewer 3 that our highly idealized experimental design warrants a more cautious and nuanced discussion regarding its translation to real-world policy and land-based mitigation strategies. To ensure that our policy implications are not overstated, we have added/revised text in the Discussion to emphasize that our results only speak to the biogeochemical effect of temporary carbon storage, and to reclassify storage-years as "a potential mechanism to value non-permanent sequestration" rather than "a clear metric for valuing non-permanent sequestration". The relevant revisions are listed here:

> *(Original): "Our results show that temporary carbon storage delivers measurable climate benefits that persist beyond the lifetime of the stored carbon."*
> *(Revised Discussion): "Our results show that <u>the biogeochemical effect of</u> temporary carbon storage can deliver measurable climate benefits that persist beyond the lifetime of the stored carbon."*

(Original): "Despite these complexities, the robust relationship we identify between carbon storage-years and avoided changes in inertial variables provides a clear metric for valuing non-permanent sequestration."
(Revised Discussion): "Despite these complexities, the robust relationship we identify between carbon storage-years and avoided changes in <u>some</u> inertial variables provides a <u>potential mechanism to</u> value non-permanent sequestration <u>across the storage magnitudes we explore here.</u>"

(Original): "Our results indicate that if these pledges are fulfilled, but the carbon is only temporarily stored, there would still be measurable twenty-first century climate benefits."
(Revised Discussion): "Our results indicate that if these pledges are fulfilled, but the carbon is only temporarily stored, there would still be measurable twenty-first century climate benefits from the <u>biogeochemical impacts</u> of carbon storage. <u>However, the actual net benefit of these real-world pledges, particularly those involving nature-based means, remains highly contingent on biogeophysical effects which may counteract this biogeochemical cooling effect."</u>

8. Minor comments
- There are several minor typographical issues in the manuscript (e.g., "DISSCUSSION").

Edited

- Some figure captions would benefit from clearer descriptions of the storage pathways and scenario assumptions.

We thank Reviewer 3 for bringing this to our attention. We have reviewed an edited all figure captions for clarity.

- The statistical characterization of the quasi-linear relationships could be strengthened by reporting regression slopes and uncertainty ranges in addition to R2 values. In addition, it would be helpful to indicate the range of storage magnitudes and scenarios over which the linear approximation holds. Providing confidence intervals for the fitted relationships or residual diagnostics would allow readers to better assess the robustness of the proposed scaling.

We thank the reviewer for this constructive suggestion to enhance the statistical rigor of our scaling framework. In response, we have strengthened our statistical characterization by adding 95% confidence intervals to our proportionality plots and reporting the RMSE alongside the R2 values for the proportionality plots that see scenario independent proportionality. Furthermore, we have compiled a summary table (Supplementary Table 1) in the Supplementary Information that details the calculated regression slopes, R2 values, RMSE values, and their corresponding uncertainty ranges to provide readers with full transparency. We have also updated the manuscript text to explicitly define the operational domain of our framework, clarifying the exact

range of simulated storage magnitudes over which the quasi-linear approximation holds robustly without triggering threshold feedbacks:

> *(Added text to the Results): "For globally averaged ocean variables examined here, the quasi-linear scaling relationship between storage-years and mitigated climate changes holds across the full range of temporary storage magnitudes simulated here, apart from total ocean carbon in 2300, where there is less proportionality with storage-years (see Supplementary Table 1).*

> *(Added table to Supplementary Information):*

time	variable	R2	m	ci	RMSE
2100.5	Relative sea level height	0.998	-5.93E-07	[-5.96e-07, -5.9e-07]	7.31E-05
2100.5	Global average ocean temperature	0.995	-9.06E-07	[-9.14e-07, -8.99e-07]	0.00019
2100.5	Global average total ocean carbon	0.994	-0.000473	[-0.000477, -0.000468]	0.112
2300.5	Relative sea level height	0.841	-3.17E-07	[-3.33e-07, -3e-07]	0.000412
2300.5	Global average ocean temperature	0.815	-4.81E-07	[-5.09e-07, -4.53e-07]	0.000692
2300.5	Global average total ocean carbon	0.657	-0.000146	[-0.000157, -0.000135]	0.269

*Supplementary Table 1 – **Regression summary table.** This table shows the R2, regression slope (m), 95% confidence intervals (ci), and RMSE for the regressions of variables with scenario independent proportionality with storage- years found in Figure 5 of the main manuscript.*

> *(Revised Figure 5 showing 95% confidence intervals and RMSE):*

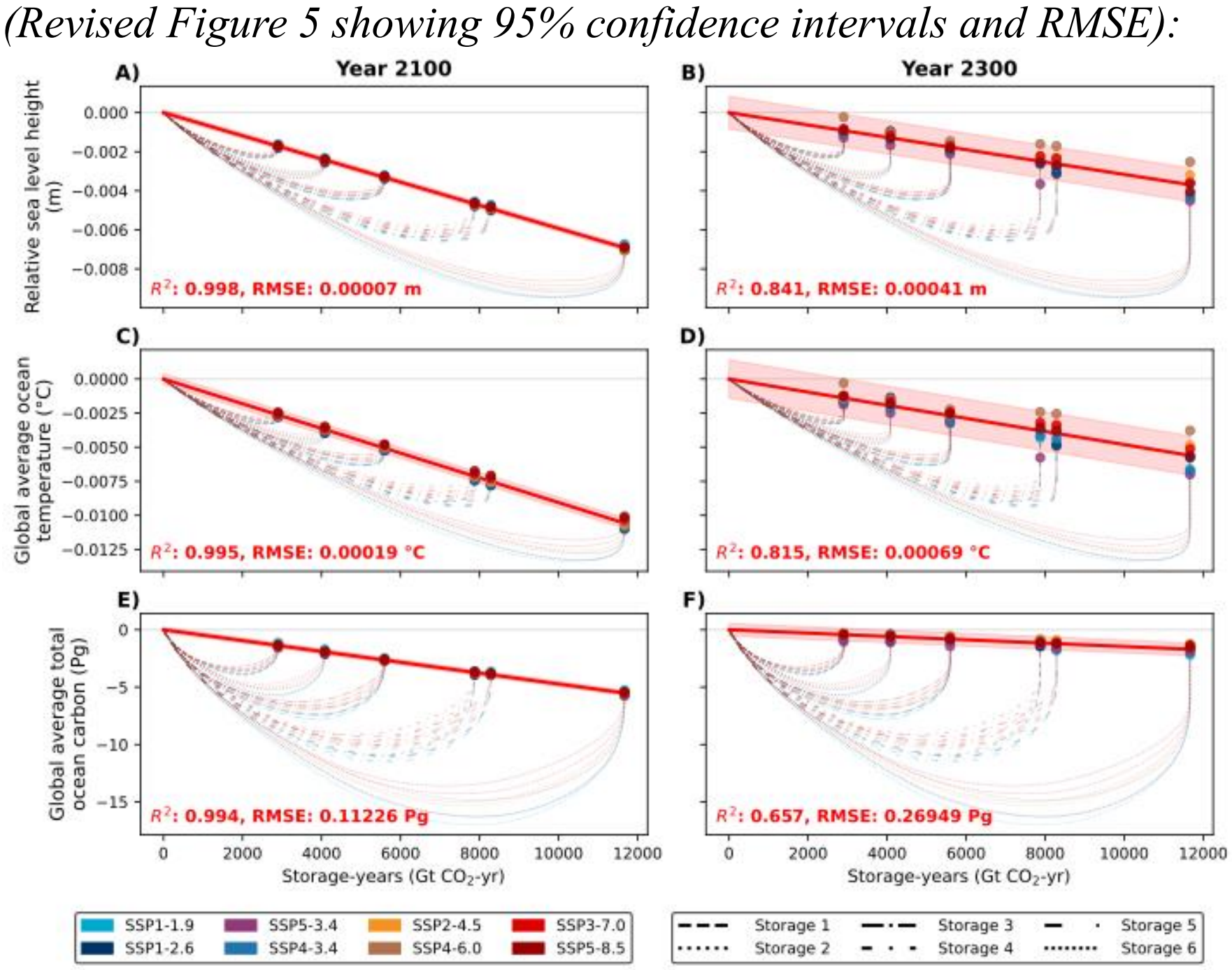

For the individual, scenario-dependent variables (i.e., AMOC and permafrost carbon), we have chosen not to provide these residual diagnostics because each regression line relies on only six data points. Providing complex statistical parameters on a sample size this small can be misleading. To clarify this point, we have added the following caveat to the Description of the regression table in the supplementary information:

> *(Added text to the regression table caption in the Supplementary Information): "AMOC and permafrost carbon are not included because their scenario-dependent proportionalities are calculated within each SSP ensemble, thus each regression relies on only six data points. These relationships are therefore inherently less robust than the scenario-independent proportionality derived from the fully aggregated multi-scenario dataset.."*

We believe these additions directly resolve your request and significantly improve the transparency and robust bounding of our findings.

Review of "Temporary carbon storage can mitigate slow-responding climate changes"

By Mitchell Dickau and H. Damon Matthews

*****Reviewer comments in bold. Author responses italicized and un-bolded.*****

REVIEWER COMMENTS

Reviewer #1 (Remarks to the Author):

General comments:
The authors have largely taken my comments into account and resolved most critical aspects. The manuscript has improved in terms of breadth of audience and rigor. However, the manuscript does not seem publication-ready to me. Firstly, I strongly suggest to tone down the claims on the generality of the storage-years – avoided changes linearity even further. The authors have not provided additional evidence for its validity across a relevantly broad range of time scales. Additionally, the increased attention to the evolution until 2300 (that I appreciate) reveals its limits on longer time scales irrespective of different storage durations. Secondly, the value of the new modelling protocol concerning the use of SSP3-7.0 land use CO_2 emissions as a baseline for all scenarios seems to be overstated with this giving a "neutral" baseline. Thirdly, figure design should be improved to optimize accessibility and interpretability. Finally, I suggest to improve the so far jargon-heavy language given the broad readership of the journal.

We thank Reviewer 1 for their constructive feedback and recognition of the improvements to our manuscript. In this revision, we have further toned-down claims regarding the generality of the storage-years framework by explicitly detailing how we do not investigate the effect of storage duration, as well as detailing the heightened uncertainty and scenario dependence that emerge by 2300. We have also reframed the SSP3-7.0 land-use baseline strictly as an experimental protocol to test perturbations without pre-existing carbon removal rather than an inherently "neutral" real-world narrative. Additionally, we have added a figure to help illustrate how the linearity between storage-years and degree-years changes from 2100 to 2300, and we have simplified technical phrasing across the text to improve readability for the journal's broad audience. Responses to each specific comment are provided below.

Line-by-line comments:

Line 19.21: The wording with doubled "that" is bulky, please improve the flow of this sentence.

We agree with Reviewer 1 and have edited this part of the abstract to improve the flow.

> *(Original): Here we show that temporary carbon storage results in avoided changes in inertial climate variables that are proportional to carbon storage-years across a wide range of removal amounts and emissions scenarios. Carbon storage-years scale quasi-linearly with avoided changes in permafrost carbon, ocean carbon, AMOC, ocean temperature, and thermosteric sea-level rise, with avoided changes in some variables that persist for several centuries.*
>
> *(Revised): Here we show that temporary carbon storage results in avoided changes in inertial climate variables. Avoided changes in 2100 scale quasi-linearly to carbon storage-years across a wide range of storage amounts and emissions scenarios for permafrost carbon, ocean carbon, AMOC, ocean temperature, and thermosteric sea-level*

rise. By 2300, proportionality between avoided changes and storage years only persists in some inertial climate variables.

Line 21-24: Qualify/detail this with the main limit to this linearity you find.

In agreement with Reviewer 1, we have added more detail to the abstract to highlight the limits of the avoided changes vs. storage year proportionality.

(Original): Carbon storage-years scale quasi-linearly with avoided changes in permafrost carbon, ocean carbon, AMOC, ocean temperature, and thermosteric sea-level rise, with avoided changes in some variables that persist for several centuries.

(Revised): Avoided changes in 2100 scale quasi-linearly with carbon storage-years across a wide range of storage amounts and emissions scenarios for permafrost carbon, ocean carbon, AMOC, ocean temperature, and thermosteric sea-level rise. By 2300, proportionality between avoided changes and storage years persists in some of these inertial climate variables, notably sea-level rise, ocean temperature and ocean carbon.

Line 87: "baseline simulation for each of the SSPs" can be confusing for scholars in the pathway modeling community as they use "baseline" for the SSP scenarios without RCP constraints. Moreover, the jargonized term "SSPs" typically is used to refer to the class of all scenarios sharing the same SSP, irrespective of RCP. Try rephrasing to "and eight reference simulations following the SSP-RCP scenarios established in CMIP6 using SSP1-1.9, ..., and SSP5-8.5 to generate..."

We agree with Reviewer 1 and have followed their suggestion to improve clarity.

(Original): Our simulations include six temporary storage pathways and one baseline simulation for each of the SSPs – SSP1-1.9, SSP1-2.6, SSP4-3.4, SSP5-3.4OS, SSP2-4.5, SSP4-6.0, SSP 3-7.0, and SSP5-8.5 – to generate an ensemble of 48 temporary storage simulations (hereinafter storage SSPs) and eight baseline simulations (hereinafter baseline SSPs) (Fig. 1).

(Revised): Our simulations include eight reference simulations following the SSP-RCP scenarios established in CMIP6 – SSP1-1.9, SSP1-2.6, SSP4-3.4, SSP5-3.4OS, SSP2-4.5, SSP4-6.0, SSP 3-7.0, and SSP5-8.5 – and six temporary storage pathways applied to each SSP-RCP reference simulation to generate an ensemble of 48 temporary storage simulations (hereinafter storage SSPs) and eight reference simulations (hereinafter reference SSPs) (Fig. 1).

Line 90: "baseline SSPs" as detailed above, jargon that is different from the community-specific language and therefor confusing for large parts of the pathway modeling community. Adapt in the entire manuscript please.

We have changed the terminology throughout the manuscript and supplementary information from "baseline SSPs" to "reference SSPs" in response to Reviewer 1's helpful comment.

Line 90, Methods reference: given your frequent relevance justification with land-based CDR and such being defined in the SSP-RCP forcings, you need to clarify your handling of land use already here to not confuse the readers concerning potential double accounting you actually avoid.

We appreciate Reviewer 1 pointing out this omission. We have added the following text to help clarify our methods:

> *(Added text): To avoid producing unrealistic carbon dioxide removal pathways by adding temporary storage onto reference SSPs that already include ambitious removal, we adjust land use change CO2 emissions in reference SSPs to match those from SSP3-7.0, which is the only reference SSP in which land use emissions remaining positive throughout the 21st century. See Methods for more details.*

Line 92 "in both 2100 and 2300": you actually present their evolution over time. You do more than just looking at these two moments in time.

Agreed. We have edited this sentence to reflect this.

> *(Original): We then quantify the effect of this temporary carbon storage on changes in key slow-responding climate system variables in both 2100 and 2300.*
>
> *(Revised): We then quantify the effect of this temporary carbon storage on changes in key slow-responding climate system variables <u>up to 2300</u>.*

Line 94: consider starting a new sentence after "(AMOC)".

We have revised this sentence in accordance with Reviewer 1's comment:

> *(Original): We focus here on changes in permafrost carbon, ocean temperature, total ocean carbon, thermosteric sea level rise, and maximum Atlantic meridional overturning circulation (AMOC), which represent climate variables in the UVic-ESCM that include a high inertial component in their response to changing climate forcing.*
>
> *(Revised): We focus here on changes in permafrost carbon, ocean temperature, total ocean carbon, thermosteric sea level rise, and maximum Atlantic meridional overturning circulation (AMOC). These variables represent climate variables in the UVic-ESCM that include a high inertial component in their response to changing climate forcing.*

Line 105: If understand your interpretation right, you do not expect this to appear similarly in other models. If this is true, why not declare it with a wording like "...2100, model-specific ocean dynamics remove this proportionality by 2300".

We thank Reviewer 1 for raising this point and giving us the opportunity to clarify our meaning. We did not intend to suggest that this behavior is an artifact unique to our specific model.

Because multi-model intercomparison of emissions driven experiments spanning these extended timescales are limited, we cannot definitively state whether this response is unique to this model

or a robust feature across Earth System Models (ESMs). Characterizing it as "model-specific ocean dynamics" might prematurely dismiss a potential physical mechanism as a model artifact.

To clarify that this result stems from physical ocean processes while remaining neutral regarding multi-model agreement, we have revised the text as follows:

> *(Original): Conversely, while the AMOC exhibits a similar scenario-dependent relationship in 2100, this proportionality breaks down completely by 2300.*
>
> *(Revised): Conversely, while the AMOC exhibits a similar scenario-dependent relationship in 2100, this proportionality breaks down completely by 2300 due to decadal-scale ocean dynamics that affect the AMOC behaviour after the year 2100.*

Line 110-113: Are you able to include a number of typical relative overestimation like in lines 238-240?

We have decided to keep the original wording of the introduction where we describe the benefit overestimation results qualitatively. We've chosen this because it is difficult to succinctly summarize these results using quantitative markers given that the benefit overestimation is quantified in different units for each variable.

However, in recognition of Reviewer 1's comment, we have added text to the Results "Contextualizing the Climate Benefits of Temporary Carbon Storage" section. Here, to accompany the more qualitative description of the benefit overestimation results in the submitted manuscript, we have added the following quantitative assessment.

> *(Added text): The maximum added benefits from permanent removal by 2300 are 3.3cm of avoided thermosteric sea-level rise, 0.05 °C of avoided ocean heating, and 32 Pg C of avoided ocean carbon uptake (Fig. 8 I, L, O).*
>
> *(Added text): By 2300, permanent carbon removal can avoid up to 4.2 Pg C permafrost carbon loss relative to temporary carbon removal (Fig. 8 C).*

Line 147: "see temporary storage" complicated language (if even correct, I am not a native speaker). Please simplify.

We have modified the sentence in question to improve clarity.

> *(Original): After 2100, SSPs with peak-and-decline temperature trajectories see temporary storage cause larger temperature differences and increased variability relative to their baselines in comparison to high-emissions SSPs that do not reach net-negative CO2 throughout the simulations (Fig. 1 F).*
>
> *(Revised): After 2100, the temperature differences and increased variability caused by temporary storage are greater in SSPs with peak-and-decline temperature trajectories than in high-emissions SSPs that do not reach net-negative CO2 emissions throughout the simulations (Fig. 1 F).*

Line 149 "net-negative CO2": add "emissions"? This is a typical instance where the wording is overly shortened and jargonized. Please also work on other, similar instances.

Corrected.

Line 156-158: I appreciate the note on regional temperature patterns. In my original comment on spatially explicit results, I was referring to impacts like permafrost carbon and ocean carbon. I understand that a detailed study of impact patterns is beyond the scope of this study that focuses on richness in scenario space. However, one or two sentences on spatial features that explain some time series behaviour would help (thinking of permafrost carbon stock variations in SSP2-4.5 and SP1-2.6). I also appreciate a negative statement about spatial features or, better to say, a statement about the homogeneity of the responses in UVic-ESCM.

We thank Reviewer 1 for clarifying the intent of their original comment in the first round of reviews.

In response to Reviewer 1's comment, we have added the following text to the Discussion to acknowledge that future spatial analysis is an important next step.

> *(Added text): Interdependencies among these variables can also shape long-term outcomes, as demonstrated by recent evidence that AMOC weakening can partially offset permafrost carbon loss in overshoot scenarios via AMOC driven regional cooling in the North Atlantic [43]. While our analysis focuses on global-scale trends across a broad scenario space, these AMOC-permafrost interdependencies underscore the importance of future spatial analyses, which may help explain the increasing scenario dependence observed toward 2300.*

We've also revised/added text to the Discussion highlighting the spatial homogeneity of the UVic-ESCM.

> *(Original): Future multi-model intercomparisons would be an important next step to verify whether this storage-year proportionality remains robust for other variables across different climate model structures and complexities.*
>
> *(Added text): Future multi-model intercomparisons <u>are</u> an important next step to verify whether this storage-year proportionality remains robust for other variables across different climate model structures and complexities. <u>Additionally, exploring the spatial patterns of these responses in other models is also an important priority, especially since the UVic-ESCM's single-layer atmosphere and prescribed circulation limit its ability to draw detailed spatially-explicit conclusions.</u>*

Line 204/205: this sounds as if it still exists, just with scenario-dependent slopes. But Fig. 2C sems to suggest the relationship to break down in some scenarios (although the vizualization makes it hard to tell). Please clarify and/or correct me should I have misunderstood the figure.

We thank Reviewer 1 for raising this point. We appreciate the opportunity to clarify the multi-century behavior shown in Figure 2C.

We do not observe a complete collapse or breakdown of the relationship between storage years and degree years. Rather, a clear linear pattern persists, both across SSP ensembles and within SSP ensembles. However, this linearity is accompanied by higher uncertainty and increased divergence across scenarios by 2300. We have produced an additional figure in the supplementary information to highlight this finding.

> *(New Supplementary Figure 3):*

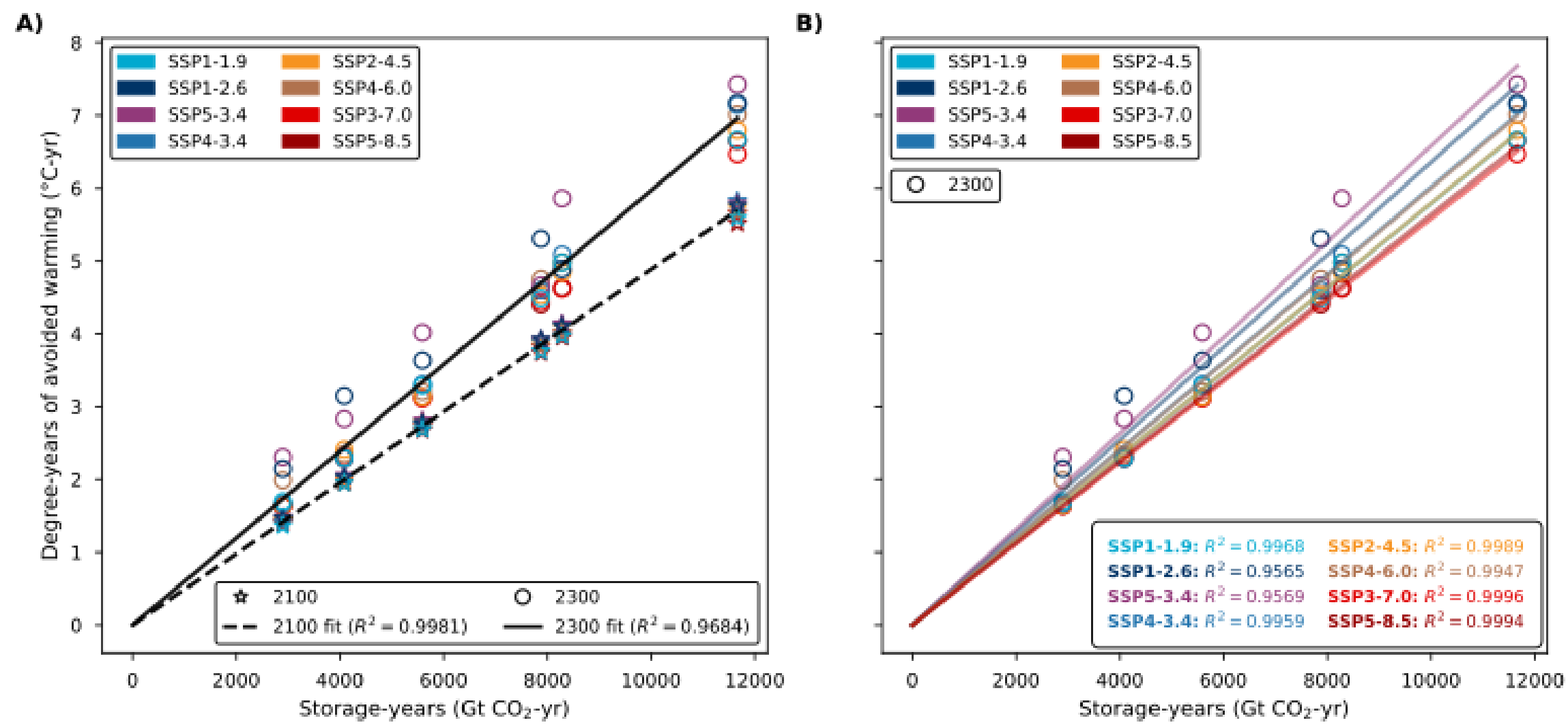

The across SSP ensemble 2300 R2 is 0.968, and the within SSP ensembles do not have R2s lower than 0.957.

To articulate both the persistence of the overall relationship and the increased scenario-dependent uncertainty, we have revised lines 204–205 as follows:

> *(Original): Consequently, the quasi-linear relationship between storage-years and degree-years becomes more scenario dependent by 2300 (Fig. 2 C).*
>
> *(Revised): Consequently, while a clear linear pattern between storage-years and degree-years persists through 2300 across ensembles (R2 0.968), it is accompanied by stronger scenario dependence and has a different slope relative to that in 2100 (Fig. 2 C, Supplementary Fig. 3).*

Line 210 "persistent": persistance sounds more stable than most elements of the climate system you assess seem to be. I think "non-zero" would be a careful wording but maybe "continued but declining" would improve accuracy overall.

We have adopted Reviewer 1's suggestion.

> *(Original): Unlike the effects of temporary carbon storage on temperature, slow-responding climate variables show persistent avoided changes in 2100 despite temporary carbon storage having already been re-emitted by 2100 (Fig. 4).*

(Revised): Unlike the effects of temporary carbon storage on temperature, slow-responding climate variables show continued but declining avoided changes in 2100 despite temporary carbon storage having already been re-emitted by 2100 (Fig. 4).

Line 259-261: I believe already here, it needs to be made clear that your analysis only allows to infer this for varying magnitudes, not for varying durations.

We thank Reviewer 1 for this suggestion and have taken the opportunity to clarify this finding:

(Original): This suggests that avoided end-of-century changes in these variables can be inferred directly from the magnitude and duration of carbon storage measured using storage-years, regardless of the background emissions scenario.

(Revised): This suggests that, across the durations of storage explored here, avoided end-of-century changes in these variables can be inferred directly from the magnitude of carbon storage measured using storage-years, regardless of the background emissions scenario.

Line 265 "further from when": improve the wording. at least "further from the moment in time, when"?

Agreed. We have revised the text as follows.

(Original): The strength of the linear relationships between storage-years and avoided change are also weaker in 2300 than in 2100 (Fig. 5), indicating that there is more scenario dependence further from when temporary storage occurs for these globally averaged ocean variables.

(Revised): The strength of the linear relationships between storage-years and avoided change are also weaker in 2300 than in 2100 (Fig. 5), indicating that scenario dependence increases as more time elapses following temporary storage for these globally averaged ocean variables.

Line 445-449: make sure to discuss in comparison to the closely related

Steinert, N.J., Schwinger, J., Burke, E. et al. Permafrost carbon release scales linearly with overshoot warming mediated by AMOC tipping. Nat Commun 17, 6395 (2026). https://doi.org/10.1038/s41467-026-73612-0

Agreed. We have added the following text to the Discussion in response to Reviewer 1's comment:

(Added text): Interdependencies among these variables can also shape long-term outcomes, as demonstrated by recent evidence that AMOC weakening can partially offset permafrost carbon loss in overshoot scenarios via AMOC driven regional cooling in the North Atlantic [43].

[43]Steinert et al., 2026

Line 452-457: how do you see your results depending on the degree of TCRE reversibility (cooling per removal as a separate phenomenon to ZEC)?

We thank Reviewer 1 for highlighting the role of TCRE reversibility. We have added the following text to the Discussion to underscore that TCRE reversibility/asymmetry, which is closely related to ZEC, would change the storage-year proportionality found in our study.

> *(Original text): We expect that our conclusion of proportionality at the year 2100 would hold across other models given the consistent linearity of the TCRE relationship among models, but that changes after 2100 would be more sensitive to differences in ZEC across models.*
>
> *(Added text): We expect that our conclusion of proportionality at the year 2100 would hold given the consistent linearity of the TCRE relationship among models. Beyond 2100, however, climate responses would become more sensitive to model-dependent ZEC values and to the possibility of model-dependent TCRE asymmetry between emissions and removals, the latter being relevant primarily in scenarios reaching net-negative CO2.*

Line 462-464: here, again consider Steinert et al., 2026, https://doi.org/10.1038/s41467-026-73612-0

Agreed. We have added a sentence highlighting that proportionality between time-integrated warming and permafrost carbon loss has been demonstrated in other studies/models

> *(Original): Future multi-model intercomparisons would be an important next step to verify whether this storage-year proportionality remains robust across different climate model structures and complexities.*
>
> *(Revised): Despite these potential inter-model differences, elements of the proportionality found here are consistent with existing studies. For instance, a proportional relationship between permafrost carbon loss and time-integrated warming has been shown in a full Earth system model, intermediate-complexity models, and a land surface model [25,43]. Future multi-model intercomparisons are an important next step to verify whether this storage-year proportionality remains robust for other variables across different climate model structures and complexities.*
>
> *[43] Steinert et al., 2026*
>
> *[25] Dickau et al., 2026*

Line 494 " avoided change": clarify BGC only again in this sentence. Your measure does not account for the net avoided change.

We have taken Reviewer 1's suggestion and clarified that we are only referring to avoided change caused by the biogeochemical effect

> *(Original): By providing a measure of avoided change for climate outcomes in slow-responding variables, the metric of storage-years offers a framework to weigh these*

benefits of temporary storage against the benefits of more permanent mitigation options, such as sustained reductions of fossil CO2 emissions or durable geologic storage.

(Revised): By providing a measure of avoided biogeochemically driven change in slow-responding variables, the metric of storage-years offers a framework to weigh these benefits of temporary storage against the benefits of more permanent mitigation options, such as sustained reductions of fossil CO2 emissions or durable geologic storage.

Line 542-543: this makes a lot of sense, but choosing SSP3-7.0 land use CO2 emissions and not zero emissions is a surprising choice. You argue that "adding large further 545 storage perturbations to these SSPs would likely produce carbon removal pathways that are quite unrealistic from a technical, social, and/or economic sense" but how should the combination of continued deforestation in most of Africa be more consistent or realistic in combination with your removal pathways (especially the high-removal volume ones)? Why not choose a no Land use change reference case that allows for a cas much closer to what you claim to be "neutral"? Like this, you increased comparability among scenarios but did not increase realism in my opinion. Consider revising the modeling protocol or tone down your claims about "neutrality".

We thank the reviewer for the opportunity to clarify the rationale behind using SSP3-7.0 land-use emissions.

A key aspect of our experimental design is that our simulations do not model spatially explicit land-use change, regional land allocation, or localized land competition (such as regional deforestation in Africa paired with afforestation elsewhere). Instead, land cover is prescribed and held fixed throughout all simulations, and the experiments are entirely emissions-driven. The temporary carbon storage perturbations are implemented by modulating global prescribed emissions time series to isolate the climate-carbon cycle response to distinct global atmospheric CO_2 *trajectories.*

In this context, adopting SSP3-7.0 land-use emissions is not intended to represent an explicit regional land-use policy narrative, but rather to establish a dynamic, positive emissions trajectory from the standard CMIP6 ensemble. Choosing SSP3-7.0 provides an established, time-evolving reference scenario where land-use emissions remain positive throughout the 21st century, ensuring that the applied perturbations represent the sole carbon removal intervention.

We also considered a zero land-use emissions baseline case. However, we would argue that imposing zero land-use change emissions across all SSPs is an equally idealized assumption given that it would also be inconsistent with the evolution of other specific SSP variables modelled by integrated assessment models.

In response with the Reviewer's comment, rather than altering the modeling protocol, we have toned down our claims regarding "realism" and "neutrality." We have revised the Methods section to make explicit that this standardization operates strictly at the level of global emissions pathways without simulating spatial land-use dynamics.

(Original): In recognition of this, we replaced the land-use CO2 emissions of every SSP with values from the SSP3-7.0 scenario because it is the only SSP where land-use CO2

emissions remain positive throughout the 21st century. This provides a neutral baseline for applying temporary and permanent storage pathways.

(Revised):"In recognition of this, we replaced the land-use CO_2 emissions of every SSP with values from the SSP3-7.0 scenario, as it is the only SSP where land-use CO_2 emissions remain positive throughout the 21st century. This provides a <u>*standardized reference scenario free of pre-existing carbon dioxide removal, ensuring that experimental storage pathways are not stacked onto already ambitious land-sink assumptions. Because our simulations are emissions-driven and keep land cover fixed rather than modeling spatially explicit land-use change, this standardization serves purely to explore global emissions pathways against an established positive land-use emissions background without pre-existing carbon removal."*</u>

Reviewer #2 (Remarks to the Author):

I thank the authors for their thorough revisions in response to the reviewers' comments. They have fully addressed all of my concerns. I recommend the manuscript for publication in its current form.

We thank Reviewer 2 for their helpful reviews and for their recommendation for publication.

Reviewer #3 (Remarks to the Author):

I thank the authors for their careful and thorough response to the reviewers' comments. The manuscript has been substantially improved. As a minor suggestion, the authors may also wish to cite one or two more recent comparative studies on climate metrics for temporary carbon storage and land carbon fluxes to complement the historical overview, e.g. Brandao et al., 2019, 2024.

We would like to thank Reviewer 3 for their helpful reviews.

In response to Reviewer 3's minor suggestion, we have added the Brandao et al. 2019 citation in the Introduction.
